# Specificity of the osmotic stress response in *Candida albicans* highlighted by quantitative proteomics

**DOI:** 10.1038/s41598-018-32792-6

**Published:** 2018-09-27

**Authors:** Mette D. Jacobsen, Robert J. Beynon, Lee A. Gethings, Amy J. Claydon, James I. Langridge, Johannes P. C. Vissers, Alistair J. P. Brown, Dean E. Hammond

**Affiliations:** 1 Medical Research Council Centre for Medical Mycology at the University of Aberdeen, Aberdeen Fungal Group, Institute of Medical Sciences, Foresterhill, Aberdeen AB25 2ZD United Kingdom; 2 Centre for Proteome Research, Institute of Integrative Biology, University of Liverpool, Crown Street, Liverpool, L697ZB United Kingdom; 3 Waters Corporation, Stamford Avenue, Altrincham Road, Wilmslow, SK9 4AX United Kingdom; 4 Cellular and Molecular Physiology, Institute of Translational Medicine, University of Liverpool, Liverpool, L69 3BX United Kingdom

## Abstract

Stress adaptation is critical for the survival of microbes in dynamic environments, and in particular, for fungal pathogens to survive in and colonise host niches. Proteomic analyses have the potential to significantly enhance our understanding of these adaptive responses by providing insight into post-transcriptional regulatory mechanisms that contribute to the outputs, as well as testing presumptions about the regulation of protein levels based on transcript profiling. Here, we used label-free, quantitative mass spectrometry to re-examine the response of the major fungal pathogen of humans, *Candida albicans*, to osmotic stress. Of the 1,262 proteins that were identified, 84 were down-regulated in response to 1M NaCl, reflecting the decrease in ribosome biogenesis and translation that often accompanies stress. The 64 up-regulated proteins included central metabolic enzymes required for glycerol synthesis, a key osmolyte for this yeast, as well as proteins with functions during stress. These data reinforce the view that adaptation to salt stress involves a transient reduction in ribosome biogenesis and translation together with the accumulation of the osmolyte, glycerol. The specificity of the response to salt stress is highlighted by the small proportion of quantified *C. albicans* proteins (5%) whose relative elevated abundances were statistically significant.

## Introduction

*Candida albican*s is most commonly found as a commensal of healthy humans but can colonise diverse niches within the human body. For example, this fungus colonises the gastrointestinal tract of most individuals, and is a frequent cause of mucosal infection in otherwise healthy people (oral thrush and vaginitis). Furthermore, *C. albicans* is common cause of life-threatening systemic disease in immunocompromised patients, these infections having high mortality rates even following antifungal therapy^1^. The mechanisms by which *C. albicans* transitions from a relatively harmless commensal to an aggressive pathogen are not fully understood, but relate to the activation of a battery of fungal virulence factors, a perturbation of the local microbiota that permits fungal outgrowth, and/or the weakening of host immunity^2–5^. In addition, a set of fungal fitness attributes, which include rapid and effective nutrient and stress adaptation, promote the virulence of *C. albicans*^6,7^. The ability to quickly adapt to host-imposed stresses in various environments contributes significantly to the survival of the fungus in host niches, and its ability to cause infection^8^. In particular, host defence mechanisms are perceived as stresses by the pathogen. Myeloid cells attempt to kill fungal cells using a range of toxic reactive oxygen and nitrogen species^9^, and it was recently shown that a combination of cationic (NaCl) and oxidative stresses are particularly lethal for pathogenic *Candida* species^10,11^. Therefore, an understanding of stress response mechanisms could potentially lead to therapeutic approaches that augment host defence.

*C. albicans* adapts to different stress inputs via specific signalling pathways. For example, adaptation to heat shock is mediated by the transcription factor Hsf1^12^, nitrosative stress adaptation is dependent on the transcription factor Cta4^13^, and resistance to oxidative stress is dependent upon the AP-1-like transcription factor Cap1, the response regulator Skn7, and the Hog1 Stress Activated Protein Kinase^14–17^. Hog1 is also crucial for resistance to osmotic and cationic stress in *C. albicans*^17,18^. Many studies have focused on the transcriptional response of *C. albicans* cells to different environmental stressors, *e.g*.^10,19–22^. Accurate assessments of these stress responses at the protein level are less common^23,24^.

Although there is a broad correlation between the response of transcript and protein, there is uncertainty in the degree to which one can infer changes to the proteome on the basis of transcriptomic analysis^25^. Whilst the increases in protein abundance observed in this study did correlate better with reported increases in transcript levels^20^ compared with previous studies^24^, a reduction in transcripts did not lead to equivalent changes in protein amounts. Thus, studies implying a decrease in protein abundance based on decreased levels of mRNA should be treated with caution. It is reasonable to suggest that, subject to translational regulation^26,27^, an increase in transcript level will often lead to a commensurate increase in protein abundance. However, the degree to which a reduction in transcript level will lead to a decline in the corresponding protein is dependent on the inherent intracellular stability of that protein. A protein with a low rate of degradation will not respond quickly, even following a dramatic decrease in the abundance of its transcript. There may be less of a disconnect when cells are dividing rapidly, as the rapid expansion of total cellular volume can dilute ‘surplus’ protein quickly. In addition, post-translational regulation plays an important role in stress adaptation. For example, while the mitogen activated protein kinase (Hog1) is important for oxidative stress resistance in *C. albicans*^17^, it does not make a major contribution to the transcriptional response to oxidative stress^20^. Therefore, to gain a thorough understanding of stress responses, they must also be studied at the proteomic level.

In this investigation, we have conducted a detailed comparative analysis of proteomic changes between *C. albicans* cells grown under normal conditions and those experiencing high osmolarity salt-stress. Previous studies^20^ have compared the transcriptome of *C. albicans* under these two conditions, and our previous analysis of the osmotic stress proteome in *C. albicans* exploited two-dimensional gel electrophoresis technologies that could not achieve the same proteome depth^24^. Therefore, in this study, we examined the osmotic stress proteome of *C. albicans* in depth using high-throughput, label-free mass spectrometry-based approaches. The relatively small proportion of proteins that were significantly up-regulated, together with the focus upon certain central carbon metabolic pathways, suggests a high degree of specificity for the osmotic stress response and highlights the significance of arabitol synthesis, as well as glycerol production, for the requisite osmolyte accumulation during cellular adaptation to this stress.

## Experimental

### Cell culture

A single *C. albicans* colony was cultured overnight at 30 °C in shaking incubator in 5 mL CAI4-CIp10 in YPD-T, pH 7.4 (Tris buffered YPD containing 2% w/v glucose, 2% w/v mycological peptone (Oxoid, Hampshire, United Kingdom), 1% w/v yeast extract (Oxoid), 100 mM Tris-HCl, pH 7.4)^28^. The following day 500 µL culture was inoculated into 50 mL YPD-T and allowed to grow overnight. Fresh medium was then inoculated with culture to OD_600_ = 0.2 and the cells were grown to OD_600_ = 0.8. At this point the culture was divided into two, diluted with medium to OD_600_ of 0.2 and NaCl added to one flask (final concentration 1M), the other serving as the control. The triplicate analysis (three high salt, three controls) were harvested after 1 h of further growth and the cell pellets were flash frozen in liquid nitrogen. Three biological replicates per treatment group were produced in this way.

### Sample preparation

Cell pellets were thawed on ice and washed in cold H_2_O. Each cell pellet was re-suspended in 250 µL H_2_O and an equal volume of acid-washed glass beads (425–600 μm, Sigma-Aldrich, St. Louis, MO) was added. The cells were disrupted using a Mini bead beater 8 (BioSpec Products, Bartlesville, OK) by 5 cycles of 10 × 20 s with 1 min on ice between each cycle. After each cycle the samples were spun down, the supernatant containing the protein extract removed, and fresh cold H_2_O added before repeating the bead beating. Finally, fractions from each sample were pooled before further analysis. High levels of cell breakage were confirmed by SDS-PAGE of different fractions.

### Proteomics

Three biological replicate samples containing a broken cell preparation equivalent to 100 µg protein were dissolved in 100 µL 25 mM ammonium bicarbonate and analysed for each condition. The proteins were denatured with 10 µL of 1% (w/v) RapiGest (Waters Corporation, Milford, MA) in 25 mM ammonium bicarbonate and incubated at 80 °C for 10 min. Next, the samples were reduced by the addition of 10 µL of 60 mM DTT followed by incubation for 10 min at 65 °C and alkylated by the addition of 10 µL of 180 mM iodoacetamide and incubation at room temperature for 30 min in the dark. Trypsin (Roche Diagnostics Ltd, West Sussex, United Kingdom) was reconstituted in 50 mM acetic acid to a concentration of 0.2 µg/µL and digestion performed by the addition of 20 µL of the trypsin solution to the sample followed by incubation at 37 °C. After 4.5 h, an additional 10 µL of trypsin solution was added and incubation continued overnight. At the end of the digestion, trifluoroacetic acid (final concentration 1% v/v) was added to inactivate and precipitate the RapiGest detergent. After centrifugation at 14,000 g for 15 min, the supernatant fraction, containing tryptic peptides, was used directly for liquid chromatography - mass spectrometry (LC-MS) analyses.

### LC-MS configuration

Nanoscale LC separation of tryptic peptides was performed with a NanoAcquity system (Waters Corporation), equipped with a Symmetry C18 5 µm, 2 cm × 180 µm precolumn and an HSS T3 C18 1.7 µm, 25 cm × 75 µm analytical reversed phase column (Waters Corporation). The samples, 1 µL (100 ng), were transferred with aqueous 0.1% (v/v) formic acid to the precolumn at a flow rate of 5 µL/min for 5 min. Mobile phase A was water containing 0.1% (v/v) formic acid, whilst mobile phase B was acetonitrile containing 0.1% (v/v) formic acid. After desalting and preconcentration, the peptides were eluted from the precolumn to the analytical column and separated with a gradient of 3–40% mobile phase B over 90 min at a flow rate of 300 nL/min, followed by a 2 min column rinse with 85% of mobile phase B. The columns were re-equilibrated at initial conditions for 20 min. The analytical column temperature was maintained at 35 °C. The lock mass compound, 200 fmol [Glu^1^]-fibrinopeptide B/ µL, was delivered by an auxiliary pump at 600 nL/min.

Mass spectrometric analysis of tryptic peptides was performed using a Synapt G2-S mass spectrometer (Waters Corporation, Wilmslow, United Kingdom). For all measurements, the mass spectrometer was operated in v-mode with nominal resolution of 20,000 FWHM. All analyses were performed in positive mode ESI. The ion source block temperature and capillary voltage were set to 100 °C and 3.2 kV, respectively. The time of flight analyser of the mass spectrometer was externally calibrated with a NaCsI mixture from *m/z* 50 to 1990. The data were post-acquisition lock mass corrected using the doubly charged monoisotopic ion of [Glu^1^]-fibrinopeptide B. The reference sprayer was sampled with a frequency of 60 s. Accurate mass LC-MS data were collected in ion mobility assisted data independent (LC-IM-DIA-MS) mode of acquisition ((U)(H)DMSE)^29^. The spectral acquisition time in each mode was 0.5 s with a 0.02 s interscan delay. In low energy MS mode, data were collected at a constant ‘Trap’ collision energy of 4 eV and a constant ‘Transfer’ collision energy of 2 eV. In elevated energy mode, the ‘Trap’ collision energy was ramped from 4 eV to 5 eV in 0.5 s and the ‘Transfer’ collision energy was set to ramp during each ion mobility separation cycle, with the ramp applying the optimum collision energy for ions at a specific drift time. The ion mobility separations were combined in the 0.5 s total elevated energy integration time. One cycle of low MS1 and elevated energy MS2 data was acquired every 1 s. The quadrupole mass analyser was operated in non-resolving mode. The mass spectrometry proteomics data have been deposited to the ProteomeXchange Consortium via the PRIDE^30^ partner repository with the dataset accession number PXD008362.

### Data processing and database searching

ProteinLynx GlobalSERVER version 3.0.2 (Waters Corporation) was used to process all data. Protein identifications were obtained by searching the *C. albicans* database (6160 entries, http://www.candidagenome.org/). To detect and monitor protein and peptide identification error rates, decoy database entries were created by reversing the original protein amino acid sequences and concatenating them with the original entries. The principle of the search algorithm has been described previously^31^. Peptide and fragment ion tolerances were determined automatically, one missed cleavage site was allowed, as well as fixed carbamidomethylation of cysteine and variable oxidation of methionine. Identifications were accepted when the protein identification exceeded a confidence limit of identification greater than 95%, analogous to the probabilistic discriminant search score approach proposed by Keller *et al*.^32^, the protein was identified in at least two experimental replicates, either technical or biological, at least two peptides per protein were identified, and an amount estimation was associated with the identification^33^, which maintained an experiment-wide based protein identification false discovery rate (FDR) of 1%. Missing data points were replicated by data imputation after filtering for valid values^34^. In short, missing values were imputed with the average value of technical/biological replicates if the relative standard deviation across replicates did not exceed 50%. In all other instances, *i.e*. when the standard deviation was greater than 50% or when the protein was not detected in any of the replicates, the 0.1% percentile value of the normalised MS2 abundance was imputed.

### Bioinformatics

Normalised protein abundances were expressed as the MS2 product ion intensity sum of the fragment ions identified to a protein over the sum of all detected product ions from all proteins detected within a given replicate experiment. As demonstrated by Daly *et al*.^35^, product ion intensities scale linearly with precursor ion intensities and are a viable alternative to estimate the concentration of a protein in cases where saturation skews the estimation of the abundance. The latter can be caused by concentration effects, *i.e*. high abundant and well ionising species, or by acquisition, where ion concentration results in an increased number of ions reaching the detection system. This leads in both instances to underestimation of concentration and incorrect relative abundances. This is however not uncommon in LC-MS analysis of high dynamic range biological samples, is observed with various analyser types^36,37^ and quantitation techniques^38^, and can be corrected for using various approaches, either mathematically, or technically, respectively. The abundance estimation method applied here is similar in principle to the method described by Thalassinos *et al*.^39^, originally described by Silva *et al*.^33^. Relative protein abundances, log_2_ and square root-transformed, assuming Poisson distribution of the data, were considered significant when the 95% confidence interval was exceeded and p < 0.05 (two tailed, type two Student’s t-test), an approach of which the principle is analogous to the method described by Richardson *et al*.^40^ when dealing with uncertainty in multi-condition experiments using Bayes-based quantitation techniques, but uses univariate statistical logic instead.

To carry out functional analysis of our proteomic data, we queried online *S. cerevisiae* database annotation(s), which are more complete than the most recent *C. albicans* annotations. NCBI ‘blastp’ was initially used to search a reviewed UniProt *S. cerevisiae* database (v090816) to determine the *S. cerevisiae* homologs of our *C. albicans* protein assignments. Annotation for the top *S. cerevisiae* hit for each *C. albicans* sequence submitted to blastp (lowest e-value and highest bit score), was used for subsequent Gene Ontology (GO) functional analysis. The ‘simplify’ option of the ‘GOCluster_Report’ function of the ‘R/Bioconductor package systemPipeR version 1.10^41^ was used to perform enrichment tests for “slim” GO terms based on a hypergeometric distribution against a background list of all proteins in the *S. cerevisiae* proteome (minimum gene set size = 3, p-value cut-off = 0.05). To generate pathway diagrams annotated with our expression data, the WikiPathways^42^ plug-in of Pathvisio version 3.2.4^43^ was used. All other data analyses and visualisations were carried out using R version 3.4.2^44^; specifically, PCA using ‘FactoMineR’ and fuzzy c-means clustering using the Mfuzz package^45^.

## Results and Discussion

### Proteomic profiling of *C. albicans*

**Figure 1:**
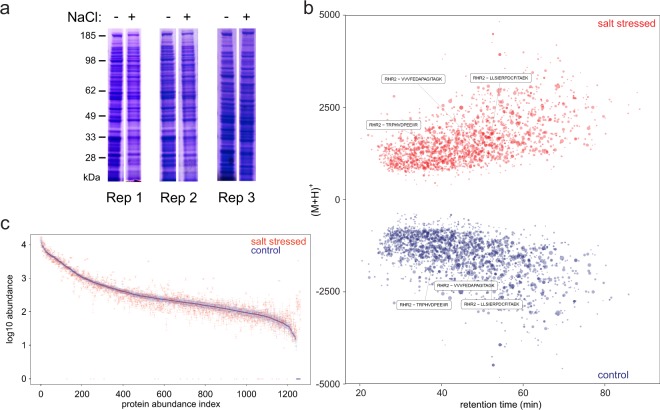
Semi quantitative profiling of *C. albicans* proteomes under normal and salt-stressed conditions. (**a**) SDS PAGE separation of three biological *C. albicans* replicates/cultures grown under different (control (−) vs. salt-stressed (+)) conditions. The gels from which these lanes were taken are shown in Supplementary Fig. 1. (**b**) Deconvoluted base peak intensity chromatograms of tryptic peptide digests of the salt-stressed and control proteomes. The peptide abundances are represented by the dot size, showing regulated Rhr2 as an example. (**c**) Protein abundances in descending order as a function of (control) protein abundance index.

To examine the impact of salt stress on the *C. albicans* proteome, cells were exposed to 1 M NaCl for 1 h. These cells retained over 98.5% viability, and the overall SDS-PAGE protein profile remained unchanged (Fig. 1a). Three biological replicates of each condition were analysed by label-free proteomics. The general, overall LC-MS profiles, shown as mirrored bubble plots, were very similar, with most peptides eluting between 25 and 75 min and 1,000–2,500 (M + H)^+^ (charge reduced peptide molecular weight) (Fig. 1b). Proteins that were identified with at least two peptides in a minimum of two replicates covered about three orders of magnitude of abundance. Highly abundant proteins included Ssa2 (Hsp70 family chaperone), Ssb1 (Hsp70 family heat shock protein), and Pdc11 (pyruvate decarboxylase), which were typically up to 100 times higher in abundance than the lowest abundance proteins, Cpa2 (arginine-specific carbamoylphosphate synthase), Dpb4 (DNA polymerase epsilon subunit D), and Csm1 (component of the monopolin complex), covering three orders of identification and quantitation dynamic range. When proteins were ordered by abundance for control cells, the salt-treated cells followed a broadly similar distribution, consistent with selective proteome changes. However, several proteins were notably increased or decreased in abundance in salt treatment (Fig. 1c).

### Overall proteomic analysis of *C. albicans*

**Figure 2:**
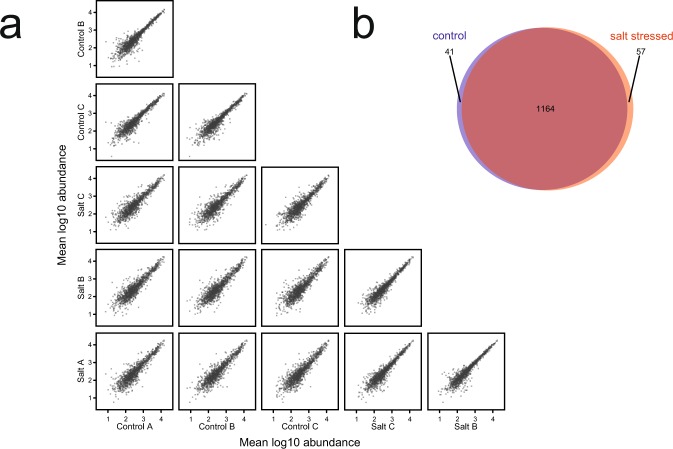
Quantitative and qualitative comparison of label-free proteome profiles. (**a**) Quantitative comparison of log-log protein abundance distributions, demonstrating biological reproducibility (control A vs. control B, control B vs. Control C, etc.) and biological variation due to salt stress (control A vs. salt A, control A vs. salt B, etc.). (**b**) Venn intersection, qualitative comparison between the two treatments groups, control vs. salt-stressed.

To explore the effect of salt treatment on the proteome of *C. albicans*, three biological replicates of each condition were analysed, each in technical triplicate. When the label-free abundance profiles of the six biological samples were compared, following averaging of each technical triplicate, the overall proteome profile was very similar and highly correlated (Fig. 2a), permitting combined analysis and comparison of the two proteomes. Label free quantitative proteomic LC-MS analyses revealed 1,262 quantifiable proteins, with very few proteins unique to one of the biological conditions. These 1,262 proteins were identified in at least two replicates with a protein false discovery rate (FDR) less than 1%. The identification replication rate, *i.e*. protein identification occurring in at least two of the three technical replicates, was 84.2% (Fig. 2a). There were 41 proteins that were exclusive to the control group, and 57 were exclusive to the salt treatment group, using the same criteria, leaving 1,164 proteins common to both conditions (Fig. 2b). Of the proteins previously identified by 2D gel analysis^24^, over 97% were detected and quantified by LC-MS and two-thirds of the 2D gel proteins that were detected lay within the highest LC-MS abundance order, as might have been anticipated. Moreover, the 2D gel-based proteomic approach can be biased against membrane (associated) proteins, whereas the LC-MS results presented here, applied to total cell extracts, afforded the identification and quantitation of 160 (12.5% of the total) membrane (or membrane-associated) proteins.

### Comparative label-free quantitative LC-MS proteome analysis of *C. albicans* under salt stress

**Figure 3:**
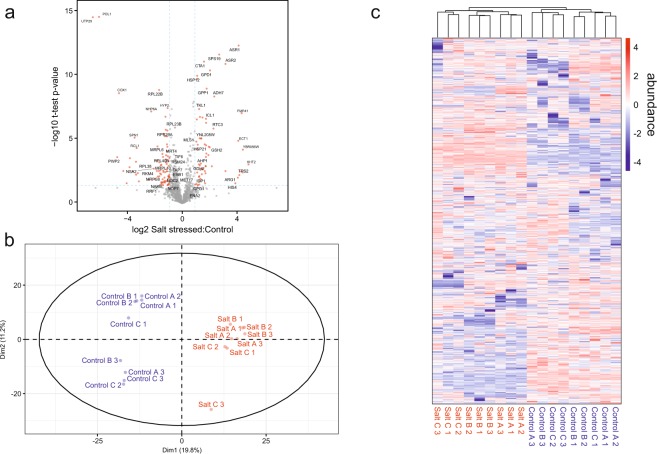
Cluster analysis of *C. albicans* label free LC-MS proteomic data under normal and salt-stressed growth conditions. (**a**) Univariate, significance (p) vs fold-change analyses highlighting several significant de-regulated proteins of interest. (**b**) Unsupervised multivariate PCA and (**c)** hierarchical clustering analyses.

Three independent replicate cultures (each with three technical replicates) of *C. albicans* were grown under normal conditions or under conditions of osmotic stress. The magnitude of change (fold change) of each protein, expressed as salt treatment/control and the t-test statistical significance (corrected for multiple comparisons) is represented in a volcano plot (Fig. 3a). Most proteins were clustered in a region of statistically insignificant differences between the two conditions. However, the abundance of a small subset of proteins was markedly altered between the two conditions. Unsupervised principal components analysis (Fig. 3b) using the entire proteome data set revealed a clear divergence between control and salt treatment proteomes, with tight association of most of the technical replicates. The robustness of the data is also apparent when the complete protein abundance data-set was used to direct hierarchical clustering analysis, with log_2_ transformed product ion abundances used as the input parameter (Fig. 3c). There was clear separation between the two conditions, although, as seen in the principal components analysis, there was some variance between technical replicates.

### Functional categorisation of the differential proteome

**Figure 4:**
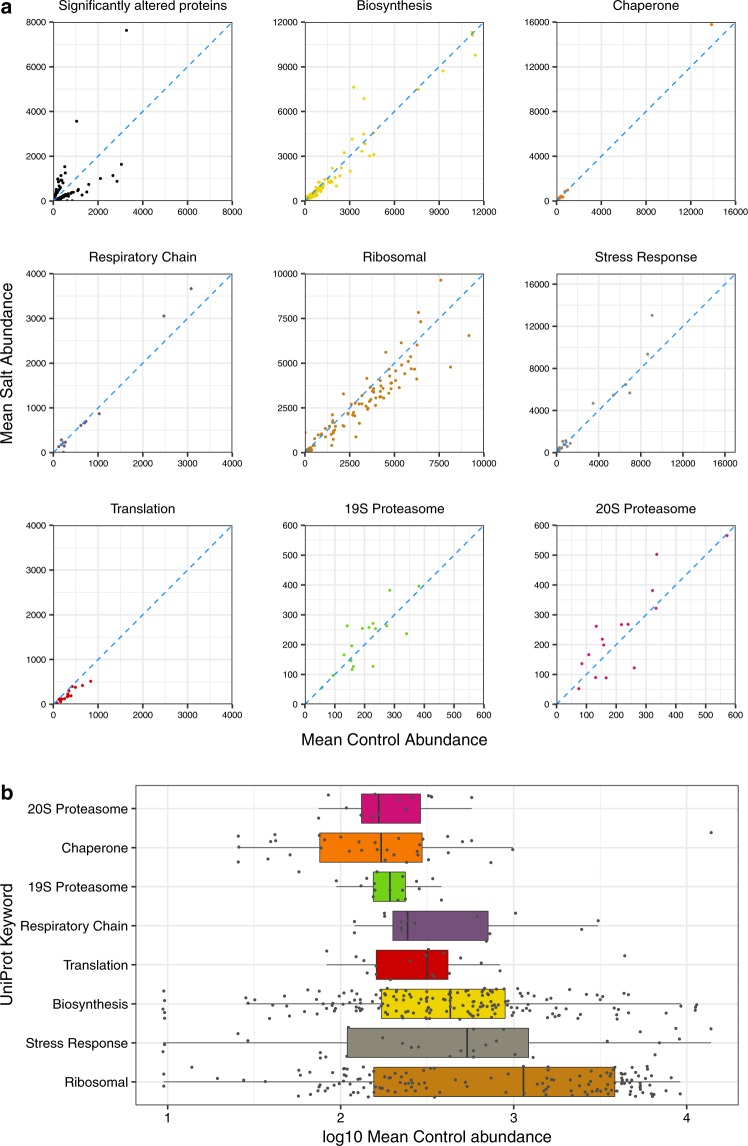
Expression changes in functional protein groupings under salt-stress conditions. (**a**) The proteome data were subclassified based on UniProt controlled vocabulary “keywords” and mean label-free abundances for each protein were compared between salt-stressed and control cells. The first (top left) panel emphasises the changes in those proteins assessed as significantly altered, summarised in Table 1. For the subsequent eight panels, the stability of the overall proteomes is emphasised for some major functional clusters. (**b**) The label-free abundances of each protein in the same eight clusters are plotted on a common set of axes, to illustrate the overall proteome stability across a broad dynamic range.

A significant proportion of *C. albicans* open reading frames have yet to be defined functionally, although many have been assigned putative roles based on homology to *S. cerevisiae* genes. Querying *S. cerevisiae* database annotation(s) following homology matching (see Experimental: Bioinformatics section), allowed us to propose a functional characterisation of the proteome data. Comparing the mean label-free abundances of proteins subclassified into functional clusters based on UniProt keywords, between salt-stressed and control cells, emphasised the overall stability of the proteome (Fig. 4a), evident across a broad dynamic range of protein abundance (Fig. 4b). The stability of the expression profile further served to highlight, and provided a good reference for, the changes in those proteins that exhibited statistically likely changes in abundance.

**Figure 5:**
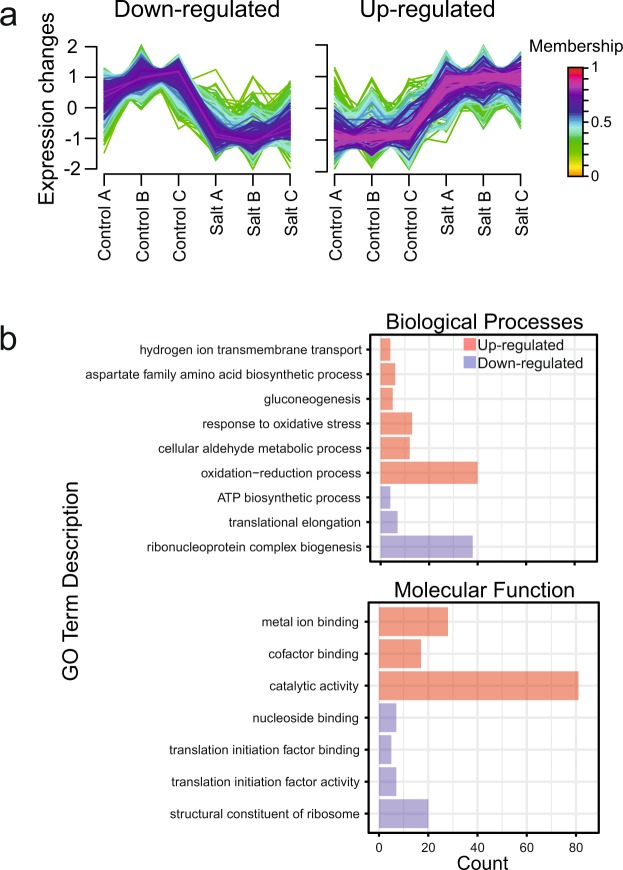
Clusters and enriched GO terms for proteins that were up- or down-regulated in response to salt stress. (**a**) Protein abundances were subjected to soft clustering analysis using a fuzzy c-means approach and only two clear clusters were evident; a cluster of 105 proteins down-regulated under conditions of salt stress (left panel), and a separate cluster of 110 proteins whose abundance increased under salt stress (right panel). Proteins with a membership value >0.5 in both clusters were subsequently used in GO enrichment tests (**b**). The most enriched GOBP (upper plot) and GOMF (lower plot) terms, based on a hypergeometric distribution, are shown.

**Figure 6:**
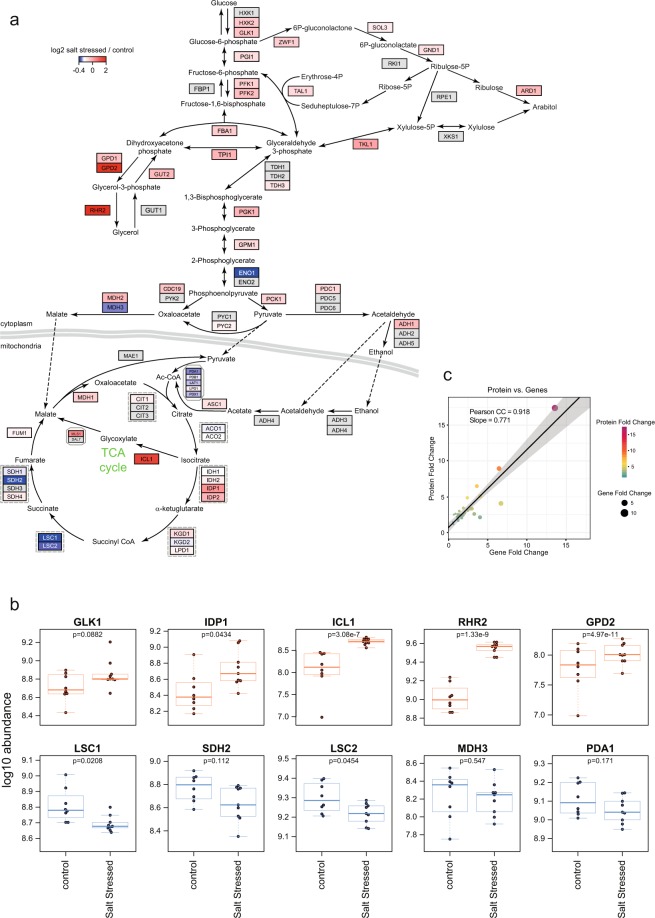
Impact of osmotic stress on central carbon metabolism. (**a**) Pathway annotation illustrating those enzymes that were detected, and their fold changes in response to the osmotic stress (see scale, top left). Any enzyme not detected in these experiments is coloured grey. The changes in selected representative proteins are illustrated in panel (**b**). For some proteins, it was possible to compare the proteome data with transcriptomic data generated by Enjalbert *et al*.^20^ in terms of relative expression of specific proteins compared to the cognate transcripts (**c**).

In parallel with the unbiased hard clustering analysis we performed to assess the reproducibility of the LC-MS data, we also subjected the abundance data to a ‘soft’ clustering approach. Two clear clusters were evident (Fig. 5a), where the first cluster contains 105 proteins that were down-regulated in conditions of salt stress, and the second cluster contains 110 proteins that were up-regulated under salt stress. Both GO enrichment analysis and pathway analysis was carried out on the proteins present in both clusters. The most enriched biological processes (upper plot) and molecular function (lower plot) terms are shown in Fig. 5b. Proteins involved in ‘metabolism’, particularly of carbohydrates (glucose) were up-regulated following osmotic stress, whereas proteins involved in ‘gene expression’, ‘translation’ and ‘translation initiation’ were down-regulated. To obtain a visual representation of the fold-change in protein abundance of the components of the ‘carbon metabolism’ pathway, the WikiPathways plug-in of Pathvisio was used to summarise the data (Fig. 6a).

### Impact of osmotic stress on central carbon metabolism

The core proteome was largely invariant in the two conditions (Fig. 2). Following exposure to 1 M NaCl, 84 proteins were significantly decreased in abundance (Supplementary Table 1), exceeding the confidence interval with p < 0.05. The majority (34) are (or are predicted to be) ribosomal proteins or proteins involved in ribosome biogenesis. Extending the analysis to moderately decreased proteins (>1.5-fold), the majority (57%) of 137 proteins are defined as ribosomal proteins or ribosome-related. They include several rRNA processing proteins such as Dim1, Ipi3, Nop14, Pno1, Pwp2, Rcl1, Utp22 as well as Utp25, which was amongst the most dramatically diminished proteins (Fig. 3; Supplementary Table 1). This group also included Pol1, a putative DNA polymerase involved in DNA replication. These data reflect the decrease in ribosome biogenesis, translation and possibly DNA replication that accompanies the transient cell cycle arrest that follows salt stress, or the imposition of other types of stress^20,25,46–48^. In addition to ribosome biogenesis and translation, the levels of Cox1 (subunit I of cytochrome c oxidase), which is one of the most strongly down-regulated proteins following osmotic stress, exhibiting a 10-fold reduction under stress (Fig. 3; Supplementary Table 1). A relative, univariate quantitative summary of all detected proteins is shown in Supplementary Fig. 2.

However, 64 proteins were increased in response to the osmotic stress (Supplementary Table 2), these proteins exceeding the regulation confidence interval at an adjusted p < 0.05. These included Gpd2 (glycerol-3-phosphate dehydrogenase; increased 4.1-fold) and Rhr2 (also known as Gpp1, glycerol 3-phosphatase; increased 3.4-fold), two enzymes required for biosynthesis of the osmolyte glycerol, a key requirement for osmo-adaptation in *C. albicans* and other yeasts^49,50^. This correlates well with the salt-mediated induction of the corresponding transcripts^17,20^. We note that Gpd2 levels increase to a greater extent than Gpd1 levels in response to the osmotic stress (Fig. 6a and Supplementary Table 2). Again, this correlates well with the available transcript profiling data for NaCl-induced stress in *C. albicans*^17,20^ and lends significant weight to the validity of the output of the osmotic stress proteome defined in this study.

In addition to glycerol biosynthetic enzymes, other central metabolic functions were induced by osmotic stress (Figs 3a and 6a,b; Supplementary Table 2). Several enzymes on the pentose phosphate pathway were up-regulated (Zwf1, Gnd1, Sol3, Tal1, Tkl1). Interestingly, this pathway links arabitol synthesis to glycolysis and glycerol production (Fig. 6), and arabitol accumulation has been implicated in stress adaptation in *C*. *albicans*^51,52^. Little work has been performed on arabitol synthesis in *C*. *albicans*^51–54^. However, cells are known to accumulate arabitol in response to osmotic stress in a manner that is only partially dependent on Hog1^51,52^. Our data reinforce the view that arabitol accumulation contributes to osmotic stress adaptation in *C*. *albicans*.

Other central metabolic functions induced by osmotic stress include the key glyoxylate cycle enzymes isocitrate lyase (Icl1) and malate synthase (Mls1). The glyoxylate cycle is thought to promote *C*. *albicans* pathogenicity, as both the *ILS1* and *MLS1* transcripts are induced during phagocytosis by macrophages^47,48,55,56^. In addition, enzymes that generate intermediates for the glyoxylate cycle were up-regulated in response to osmotic stress, *e.g*. isocitrate dehydrogenase (Idp1) and glutamate dehydrogenase (Gdh2). The up-regulated central metabolic enzymes also included the glycolytic enzymes Tpi1 and Pgk1 and two key enzymes that regulate glycolytic flux: phosphofructokinase (Pfk2) and pyruvate kinase (Cdc19) (Fig. 6a). Sorbose reductase (Sou1) was also strongly increased (6.7-fold). Sou1 is part of the sorbose utilization pathway, probably converting this sugar to fructose-6-phosphate on the glycolytic pathway^57^. Meanwhile, other central metabolic enzymes were down-regulated (Eno1, Sdh2, Lsc1) (Fig. 6a). Together, these changes may reflect the differential expression of specific isozymes, and/or the adjustment of central metabolic fluxes following the demand for increased glycerol, and possibly arabitol, synthesis. The lack of significant changes to other metabolic functions highlights the specificity of the metabolic response to osmotic stress.

The highest increases in protein abundance were manifest by Asr1 (a ubiquitin ligase that modifies RNA polymerase II) and Asr2. These proteins were identified as adenylyl cyclase and stress responsive^58^ in a study of transcriptional response of cAMP signalling in *C. albicans*, and are up-regulated following a range of other treatments including heat, osmotic^59^ and weak acid stress^22^ as well as in the response to 10% (v/v) fetal bovine serum in the medium^60^. The set of up-regulated proteins also include numerous stress proteins whose cognate genes are part of the core transcriptional response to stress in *C. albicans*^20^. These include Hsp12 (C5_02080C_A), Adh7 (C6_02480W_A) and Ahp1 (C4_02410C_A). Levels of the heat shock protein Hsp21 (C2_04010C_A), which is involved in glycerol regulation^61^, were also elevated. In addition, proteins involved in oxidative stress adaptation were induced by the osmotic stress, suggesting that this stress induced a mild oxidative stress response in *C. albicans*^62^. For example, the levels of Cat1 (catalase), Gsh2 (glutathione biosynthesis) and Glx3 (a glutathione-independent glyoxalase) were all elevated after salt stress. Significantly, Tps2 (C1_03380W_A) was also increased. Tps2 encodes trehalose-6-phosphate phosphatase, required for synthesis of the stress protectant trehalose. The *TPS2* gene is up-regulated in response to osmotic stress and contributes to ionic stress resistance in *C. albicans*^20,63^.

Comparing these proteomic outputs to those of Yin *et al*.^24^, there is reasonable overlap given the different technical approaches, with Rhr2, Grp2, Met15, Rct1, Tkl1 and Orf19.1862 being identified in both studies. Yin *et al*.^24^ used 2D gels and MALDI-ToF mass spectrometry to identify proteins that were up-regulated in response to 0.3 M NaCl. Furthermore, there is reasonable overlap with previous transcript profiling studies performed under similar conditions^10^ in that the top six proteins were also up-regulated at the transcript level (Fig. 6c). Previous work has indicated that during salt stress in *S. cerevisiae*, for low abundance proteins, the correlation between changes at protein and transcript levels is weak (R^2^ = 0.09)^25,64,65^.

## Conclusions

Label-free proteomic quantification by LC-MS has been effective in profiling of over 1,250 proteins from the *C. albicans* proteome, and in identifying the significant changes in this proteome that occur in response to exposure to 1M NaCl. The data clearly indicate that this salt stress leads to reduced levels of proteins involved in translation during the transient cell cycle arrest that accompanies this stress, as well as the increase of proteins that mediate adaptation to the stress. These include stress proteins and enzymes required for the synthesis of glycerol. The other stress-induced enzymes included components of the arabitol biosynthetic pathway, which reinforces the view that this often ignored osmolyte contributes to osmotic stress adaptation in *C. albicans*. This study opens the way to the detailed proteomic analysis of other adaptive responses that contribute to the pathobiology of this major pathogen of humans. Analysis of the proteins that are changing is underway, but classic stress responses (heat shock proteins, glycerol metabolism) are already identified as responsive at the protein level.

## Electronic supplementary material

Supplementary Material
