# Supplementary material for: Specificity of the osmotic stress response in Candida albicans highlighted by quantitative proteomics
Source: Sci Rep. 2018 Sep 27;8:14492. doi: 10.1038/s41598-018-32792-6 (PMC6160413; doi:10.1038/s41598-018-32792-6)
Supplement: Supplementary file 1 — Supplementary Material [file 41598_2018_32792_MOESM1_ESM.pdf]

Specificity of the osmotic stress response in *Candida albicans* highlighted by quantitative proteomics

Mette D Jacobsen[1,†], Robert J Beynon[2], Lee A Gethings[3,†], Amy J Claydon[2,†], James I Langridge[3], Johannes PC Vissers[3], Alistair JP Brown[1,*] and Dean E Hammond[4,*]

1. Medical Research Council Centre for Medical Mycology at the University of Aberdeen, Aberdeen Fungal Group, Institute of Medical Sciences, Foresterhill, Aberdeen AB25 2ZD, United Kingdom
2. Centre for Proteome Research, Institute of Integrative Biology, University of Liverpool, Crown Street, L697ZB, United Kingdom
3. Waters Corporation, Stamford Avenue, Altrincham Road, Wilmslow, SK9 4AX, United Kingdom
4. Cellular and Molecular Physiology, Institute of Translational Medicine, University of Liverpool, Liverpool L69 3BX, United Kingdom

† These authors contributed equally to this work

* Corresponding authors

Prof. Alistair JP Brown
Aberdeen Fungal Group, MRC Centre for Medical Mycology
University of Aberdeen, Institute of Medical Sciences
Foresterhill, Aberdeen AB25 2ZD, United Kingdom
[T] +44 1224 437482
[E] Al.Brown@abdn.ac.uk

Dr. Dean Hammond
Cellular and Molecular Physiology
Institute of Translational Medicine
University of Liverpool
Liverpool L69 3BX, United Kingdom
[T] +44 151 794 9566
[E] d.e.hammond@liverpool.ac.uk

Table 1. Down-regulated *C. Albicans* proteins grown under osmotic salt stress.

accession number	orf	feature name	gene name	ratio	log2 ratio	p	# peptides	coverage (%)	Description
CAL0001785	orf19.5083	C1_08100W_A	DRG1	0.5	-0.90	3.3E-03	16	48.6	GTP-binding protein
CAL0005770	orf19.3504	C6_02070C_A	RPL23A	0.5	-0.89	1.5E-06	8	59.4	Ribosomal 60S subunit protein L23B
CAL0003843	orf19.7238	C1_14280C_A	NPL3	0.5	-0.89	2.3E-04	10	31.6	mRNA-binding protein
CAL0002732	orf19.5195	C1_04420C_A	URA6	0.5	-0.93	2.6E-02	9	30.2	Uridylate kinase (UK) (EC 2.7.4.14)
CAL0003464	orf19.5991	C3_05160C_A	DBP10	0.5	-0.92	2.9E-02	19	14.8	ATP-dependent RNA helicase DBP10 (EC 3.6.4.13)
CAL0000023	orf19.7531	CR_00090C_A	CAALFM	0.5	-0.95	1.1E-02	7	25.3	Uncharacterized protein
CAL0002882	orf19.1236	C4_05550C_A	GVP36	0.5	-0.95	7.2E-03	13	39.7	Gvp36p
CAL0005156	orf19.2859	CR_02980C_A	SRP40	0.5	-0.94	3.5E-04	8	16.6	Srp40p
CAL0002204	orf19.3895	C5_04130C_A	CHT2	0.5	-0.98	9.6E-03	5	11.0	Chitinase 2 (EC 3.2.1.14)
CAL0004971	orf19.4093	C2_09320C_A	NOP7	0.5	-0.97	4.2E-02	17	25.7	Pescadillo homolog (Nucleolar protein 7 homolog)
CAL0005704	orf19.7484	CR_00510C_A	ADE1	0.5	-0.97	1.2E-02	12	48.5	Phosphoribosylaminoimidazolesuccinocarboxamide synthase
CAL0003056	orf19.1280	C5_04090C_A	SUI1	0.5	-1.01	6.5E-04	6	47.2	Translation initiation factor eIF1
CAL0003866	orf19.200	C2_09040W_A	orf19.200	0.5	-1.00	1.5E-03	7	36.8	Uncharacterized protein
CAL0000794	orf19.7602	CR_10270C_A	AHA1	0.5	-0.99	3.4E-02	10	15.7	Aha1p
CAL0004966	orf19.6779	C3_07220C_A	PRO2	0.5	-1.04	1.5E-02	14	21.7	Glutamate-5-semialdehyde dehydrogenase
CAL0001157	orf19.1047	C1_04130W_A	ERB1	0.5	-1.03	9.6E-03	17	15.8	Ribosome biogenesis protein ERB1
CAL0004932	orf19.3426	C6_01610W_A	ANB1	0.5	-1.07	4.5E-08	11	70.3	Eukaryotic translation initiation factor 5A (eIF-5A) (eIF-4D)
CAL0005158	orf19.6060	C1_00480C_A	GCN20	0.5	-1.07	4.8E-02	20	18.9	Putative AAA family ATPase
CAL0005794	orf19.6853	C1_04600C_A	orf19.6853	0.5	-1.05	4.6E-02	11	25.1	Uncharacterized protein
CAL0001603	orf19.1815	CR_07080W_A	TIF6	0.5	-1.05	2.6E-04	6	30.2	Eukaryotic translation initiation factor 6 (eIF-6)
CAF0007079	orf19.6415.1	CR_08480C_A	CAALFM	0.5	-1.09	2.5E-06	5	58.9	Ribosomal 40S subunit protein S29A
CAL0003033	orf19.124	C6_01170W_A	CIC1	0.5	-1.09	4.7E-05	10	31.1	Cic1p
CAL0001364	orf19.3022	C1_03280W_A	orf19.3022	0.5	-1.14	1.0E-03	11	31.3	Mitochondrial 37S ribosomal protein RSM24
CAL0004107	orf19.6672	C5_03600W_A	MDJ1	0.5	-1.12	3.9E-03	12	19.3	Mdj1p
CAL0004924	orf19.3423	C6_01630W_A	TIF3	0.5	-1.12	3.1E-05	17	36.7	Tif3p
CAF0006915	orf19.953.1	C5_00370W_A	COF1	0.5	-1.12	7.2E-06	8	67.8	Cofilin
CAL0001031	orf19.655	C1_11480W_A	PHO84	0.5	-1.12	1.1E-04	11	24.1	Phosphate transporter
CAL0003050	orf19.3962	C5_04750C_A	HAS1	0.5	-1.12	2.4E-02	16	29.2	ATP-dependent RNA helicase HAS1 (EC 3.6.4.13)
CAL0001546	orf19.5789	C2_03090C_A	ADE8	0.5	-1.16	2.7E-03	6	34.3	Phosphoribosylglycinamide formyltransferase
CAL0001661	orf19.1839	C1_10670C_A	RPA190	0.4	-1.20	4.6E-04	44	25.2	DNA-directed RNA polymerase subunit (EC 2.7.7.6)
CAL0006176	orf19.5550	C6_02770W_A	MRT4	0.4	-1.18	2.2E-04	10	52.0	Ribosome assembly factor mrt4
CAL0002295	orf19.3138	C4_06720W_A	NOP1	0.4	-1.23	2.1E-07	14	47.8	rRNA methyltransferase
CAL0001419	orf19.5732	C6_03640W_A	NOG2	0.4	-1.22	2.3E-02	16	24.8	Nucleolar GTP-binding protein 2
CAL0004804	orf19.7384	C3_06030W_A	NOG1	0.4	-1.21	2.2E-06	24	28.8	Nucleolar GTP-binding protein 1
CAL0000279	orf19.4261	C5_02490C_A	TIF5	0.4	-1.20	7.7E-04	18	38.6	Translation initiation factor eIF5
CAL0002459	orf19.5885	C3_04380C_A	SNU13	0.4	-1.26	7.1E-06	6	53.2	13 kDa ribonucleoprotein-associated protein
CAL0000683	orf19.86	C6_00850W_A	orf19.86	0.4	-1.26	3.0E-02	6	26.6	Glutathione peroxidase
CAL0005189	orf19.6074	C1_00340W_A	HBR1	0.4	-1.25	4.7E-03	8	39.4	Adenylate kinase isoenzyme 6 homolog HBR1 (AK6) (EC 2.7.4.3)
CAL0005829	orf19.6863	C4_05240C_A	VPH1	0.4	-1.31	2.3E-02	16	11.1	V-type proton ATPase subunit a
CAL0005000	orf19.6791	C3_07090W_A	HHT3	0.4	-1.30	1.2E-02	4	21.0	Histone H3.3
CAF0007031	orf19.5137.1	C7_03180C_A	HHO1	0.4	-1.37	4.6E-06	4	21.2	Histone
CAL0002373	orf19.5850	CR_05520W_A	NOC2	0.4	-1.34	2.9E-02	15	13.8	mRNA-binding ribosome synthesis protein
CAF0007021	orf19.4684.2	C4_01050C_A	RPL40B	0.4	-1.41	3.0E-04	2	13.5	Rpl40bp

CAL0005569	orf19.1569	C2_02450C_A	UTP22	0.4	-1.40	4.8E-02	25	13.8	U3 small nucleolar RNA-associated protein 22
CAL0000463	orf19.3680	C1_02230W_A	SEP7	0.4	-1.39	3.4E-03	14	15.9	Septation protein 7 (Seventh homolog of septin 1)
CAL0005873	orf19.3532	C2_05000C_A	MRPL10	0.4	-1.38	4.4E-03	9	24.4	Mitochondrial 54S ribosomal protein YmL10/YmL18
CAL0005711	orf19.7486	CR_00480W_A	MRPL6	0.4	-1.45	1.7E-04	10	30.6	Mitochondrial 54S ribosomal protein YmL16
CAL0002827	orf19.1220	C6_04040C_A	RVS167	0.4	-1.44	1.7E-02	9	14.0	Regulator of cytoskeleton and endocytosis RVS167
CAL0005880	orf19.498	CR_04140W_A	CAALFM	0.4	-1.44	2.9E-02	13	18.0	Mitochondrial 37S ribosomal protein NAM9
CAL0005804	orf19.477	CR_03950W_A	CAALFM	0.4	-1.47	3.1E-02	9	18.7	Uncharacterized protein
CAL0005878	orf19.3534	C2_05030C_A	RHO3	0.4	-1.53	1.2E-03	6	20.5	Rho family GTPase
CAL0000477	orf19.6234	C1_06760C_A	IPI3	0.4	-1.52	4.3E-02	11	19.2	Pre-rRNA-processing protein IPI3
CAL0002160	orf19.1193	C6_00330C_A	GNP1	0.3	-1.57	1.4E-03	8	11.9	Amino acid transporter
CAL0001326	orf19.3003	C1_03100W_A	orf19.3003	0.3	-1.55	6.9E-03	20	10.3	Uncharacterized protein
CAL0001053	orf19.661	CR_09510C_A	CAALFM	0.3	-1.54	2.9E-02	11	18.3	Uncharacterized protein
CAL0005507	orf19.1545	C2_02270C_A	orf19.1545	0.3	-1.62	5.8E-03	8	44.8	Mitochondrial 37S ribosomal protein MRPS8
CAL0002553	orf19.5136	C7_03200C_A	orf19.5136	0.3	-1.60	3.2E-03	5	21.5	Uncharacterized protein
CAL0005705	orf19.6828	C3_06760W_A	orf19.6828	0.3	-1.71	3.8E-03	6	15.5	Uncharacterized protein
CAF0007454	orf19.1409.1	C4_04390W_A	orf19.1409.1	0.3	-1.69	1.7E-09	5	48.0	Ribosomal 60S subunit protein L22B
CAL0004347	orf19.4705	C4_00880W_A	orf19.4705	0.3	-1.68	7.8E-04	10	9.7	tRNA adenylyltransferase
CAL0005905	orf19.6886	C2_05750W_A	orf19.6886	0.3	-1.68	9.7E-03	12	20.8	Uncharacterized protein
CAL0002680	orf19.825	C2_03990W_A	GCD7	0.3	-1.81	4.5E-05	7	10.2	Translation initiation factor eIF2B subunit beta
CAL0003420	orf19.3288	C1_00990C_A	NMA111	0.3	-1.79	1.0E-02	24	16.4	Nma111p
CAF0006935	orf19.2111.2	C2_00210W_A	RPL38	0.3	-1.90	5.8E-04	5	39.2	Ribosomal 60S subunit protein L38
CAL0003473	orf19.2654	C5_03250W_A	RMS1	0.3	-1.86	3.7E-03	12	17.0	Ribosomal lysine N-methyltransferase
CAL0005222	orf19.2884	C4_06500W_A	CDC68	0.3	-2.01	1.7E-02	23	13.2	FACT complex subunit SPT16 (CaCDC68)
CAL0004846	orf19.7398	C3_06390W_A	orf19.7398	0.2	-2.17	2.4E-03	6	24.9	Uncharacterized protein (Fragment)
CAF0007020	orf19.4623.3	C4_01700C_A	NHP6	0.2	-2.27	8.2E-08	3	35.9	Non-histone chromosomal protein 6
CAL0120690	orf19.332.1	C3_03340C_A	orf19.332.1	0.2	-2.54	1.8E-02	3	30.3	Uncharacterized protein
CAL0001325	orf19.6346	C1_12760W_A	BCP1	0.1	-3.17	2.4E-02	7	16.0	Protein BCP1
CAL0000825	orf19.7618	CR_10410C_A	PNO1	0.1	-3.39	7.1E-04	6	15.0	Pre-rRNA-processing protein PNO1
CAL0006081	orf19.2965	C1_02760W_A	GET3	0.1	-3.37	7.2E-03	5	11.6	ATPase GET3 (EC 3.6.-.-) (Arsenical pump-driving ATPase)
CAL0001795	orf19.1886	C2_07450C_A	RCL1	0.1	-3.34	5.9E-05	11	14.0	rRNA-processing endoribonuclease
CAL0001590	orf19.5010	C1_13730C_A	DIM1	0.1	-3.26	5.5E-03	7	17.8	rRNA adenine N(6)-methyltransferase (EC 2.1.1.-)
CAL0000512	orf19.6252	C1_06590C_A	IWS1	0.1	-3.49	9.1E-06	9	12.6	Transcription factor IWS1
CAL0001381	orf19.6374	CR_08130W_A	CAALFM	0.1	-3.62	3.9E-03	9	12.2	Uncharacterized protein
CAL0003384	orf19.5959	C3_04900W_A	NOP14	0.1	-3.86	1.9E-03	20	10.9	SnoRNA-binding rRNA-processing protein
CAL0004137	orf19.6686	C7_03540C_A	ENP2	0.1	-3.83	3.7E-04	13	12.5	Ribosome biosynthesis protein
CAL0003706	orf19.1305	C4_03730C_A	TRM5	0.1	-4.06	3.4E-02	12	20.6	tRNA (guanine(37)-N1)-methyltransferase (EC 2.1.1.228)
CAL0003382	orf19.5958	C3_04890W_A	CDR2	0.1	-4.31	3.7E-03	22	9.5	Multidrug resistance protein CDR2
CAL0003392	orf19.3276	CR_00800C_A	PWP2	0.04	-4.75	3.1E-04	25	14.5	SnoRNA-binding rRNA-processing protein
CAF0007377	CaalfMp08	CM_00210W	COX1	0.04	-4.62	3.0E-09	2	4.5	Cytochrome c oxidase subunit 1
CAL0001680	orf19.1849	CR_06840W_A	UTP25	0.01	-6.54	3.4E-15	15	11.4	U3 small nucleolar RNA-associated protein 25
CAL0002431	orf19.5873	C3_04300C_A	POL1	0.01	-6.08	3.2E-15	19	5.2	DNA polymerase (EC 2.7.7.7)

Table 2. Up-regulated *C. Albicans* proteins grown under osmotic salt stress.

accession number	orf	feature name	gene name	ratio	log2 ratio	p	# peptides	coverage (%)	description
CAL0001203	orf19.1061	C1_04260W_A	HHT21	27.9	4.80	1.1E-03	4	21.2	Histone H3.1/H3.2
CAL0003768	orf19.7214	C1_14060W_A	orf19.7214	21.1	4.40	7.9E-05	14	20.5	17-beta-hydroxysteroid dehydrogenase-like protein
CAL0000310	orf19.577	C5_00750C_A	orf19.577	20.8	4.38	4.8E-03	15	17.8	Uncharacterized protein
CAL0003876	orf19.7244	C1_14320C_A	orf19.7244	19.5	4.28	1.1E-07	8	28.6	Uncharacterized protein
CAL0005659	orf19.6816	C3_06860C_A	orf19.6816	17.8	4.15	4.1E-03	9	26.9	Aldo-keto reductase superfamily protein
CAL0000739	orf19.2344	C1_10740C_A	ASR1	17.4	4.12	5.6E-13	7	13.0	Asr1p
CAL0001393	orf19.3038	C1_03380W_A	TPS2	17.4	4.12	7.7E-03	20	13.9	Trehalose-phosphatase
CAL0005270	orf19.2107	C2_00260C_A	MUQ1	16.2	4.01	1.6E-05	10	28.8	Ethanolamine-phosphate cytidylyltransferase
CAL0005634	orf19.7469	CR_00620C_A	ARG1	15.9	3.99	1.2E-02	10	19.5	Argininosuccinate synthase
CAL0000622	orf19.5639	C4_00140C_A	HIS4	14.0	3.81	3.4E-02	16	15.2	Histidine biosynthesis trifunctional protein
CAL0003994	orf19.7284	CR_08890C_A	ASR2	8.9	3.16	1.5E-11	17	59.4	Asr2p
CAL0006354	orf19.7504	CR_00290W_A	CAALFM	8.4	3.08	3.8E-03	4	9.2	Uncharacterized protein
CAL0005253	orf19.2896	C4_06390W_A	SOU1	6.5	2.70	2.8E-12	10	48.2	Sorbose reductase SOU1 (EC 1.1.1.289)
CAL0006095	orf19.5517	C6_02480W_A	orf19.5517	5.1	2.34	5.5E-09	13	35.2	NADP-dependent alcohol dehydrogenase
CAL0001729	orf19.1862	C2_07630C_A	orf19.1862	4.9	2.28	1.8E-06	7	66.3	Uncharacterized protein
CAL0005506	orf19.4888	C1_10190W_A	orf19.4888	4.8	2.26	1.6E-03	3	7.8	Uncharacterized protein
CAL0001161	orf19.691	C6_02010C_A	GPD2	4.1	2.03	5.0E-11	24	77.4	Glycerol-3-phosphate dehydrogenase [NAD(+)] (EC 1.1.1.8)
CAL0004219	orf19.2737	C4_02620C_A	orf19.2737	4.0	2.00	5.2E-05	20	32.3	Putative phosphotransferase
CAL0002106	orf19.6404	CR_08370W_A	GSH2	3.9	1.95	4.6E-05	12	18.3	Glutathione synthetase (GSH-S) (EC 6.3.2.3)
CAL0001252	orf19.1084	C6_04270W_A	CDC39	3.8	1.91	2.1E-04	24	7.9	CCR4-NOT core subunit
CAL0005236	orf19.5437	C3_00320W_A	RHR2	3.4	1.78	1.3E-09	11	44.1	Glycerol-1-phosphatase
CAL0004933	orf19.4082	C2_09220W_A	DDR48	3.4	1.77	1.6E-04	9	30.4	Stress protein DDR48 (DNA damage-responsive protein 48)
CAL0000895	orf19.4309	C5_02860C_A	GRP2	3.3	1.74	6.3E-07	17	65.9	Putative NADPH-dependent methylglyoxal reductase GRP2
CAL0004989	orf19.3443	C6_01410C_A	OYE2	3.3	1.74	3.7E-05	6	11.3	NADPH dehydrogenase
CAL0002731	orf19.4538	C1_01850C_A	orf19.4538	3.3	1.73	3.0E-04	6	10.9	Uncharacterized protein
CAL0005762	orf19.6844	C1_04500W_A	ICL1	3.3	1.73	3.1E-07	17	36.2	Isocitrate lyase
CAL0003556	orf19.5243	C1_12210W_A	TRP3	3.3	1.71	1.6E-03	11	19.5	Bifunctional anthranilate synthase
CAL0003394	orf19.3278	CR_00780C_A	GSY1	3.3	1.71	1.6E-03	14	15.1	Glycogen [starch] synthase (EC 2.4.1.11)
CAL0001619	orf19.5025	C1_13870W_A	MET3	3.3	1.70	5.7E-03	15	22.4	Sulfate adenylyltransferase (EC 2.7.7.4) (ATP-sulfurylase)
CAL0000999	orf19.1796	C4_05390W_A	orf19.1796	3.2	1.67	3.5E-02	10	12.6	Glyoxylate reductase
CAL0006102	orf19.2970	C1_02820W_A	LYS2	3.1	1.65	3.8E-03	32	20.9	L-aminoadipate-semialdehyde dehydrogenase
CAL0006266	orf19.2244	C2_06890C_A	orf19.2244	3.1	1.64	3.1E-05	12	46.5	Uncharacterized protein
CAL0000462	orf19.6229	C1_06810W_A	CAT1	3.0	1.59	1.0E-11	19	50.7	Peroxisomal catalase (EC 1.11.1.6)
CAL0006358	orf19.4960	C1_13330C_A	orf19.4960	2.8	1.50	2.4E-07	5	11.0	Spermine synthase
CAL0001835	orf19.745	C4_05150W_A	VAC8	2.8	1.47	2.5E-03	8	10.9	Vacuolar protein 8
CAL0000225	orf19.4246	C5_02380W_A	orf19.4246	2.8	1.46	2.8E-03	21	42.5	Uncharacterized protein
CAL0004896	orf19.6757	C3_07340W_A	GCY1	2.7	1.43	6.7E-03	8	23.9	Glycerol 2-dehydrogenase (NADP(+))
CAL0006415	orf19.2296	C1_11200W_A	orf19.2296	2.6	1.39	1.0E-05	55	26.8	Uncharacterized protein
CAL0004665	orf19.7350	C3_05710W_A	RCT1	2.6	1.38	1.1E-05	10	51.6	Rct1p
CAL0001685	orf19.5054	C1_07840W_A	orf19.5054	2.6	1.36	9.8E-04	10	33.5	Nicotinate-nucleotide pyrophosphorylase [carboxylating
CAL0005448	orf19.1528	C2_02120W_A	orf19.1528	2.5	1.33	1.5E-02	9	21.0	RNA polymerase specificity factor
CAL0004180	orf19.2710	C4_02860W_A	orf19.2710	2.5	1.32	3.0E-03	5	18.5	Uncharacterized protein
CAL0002434	orf19.2531	CR_01470W_A	CSP37	2.5	1.29	2.2E-07	20	48.8	Csp37p

CAF0006895	orf19.410.3	C1_05560W_A	RIB4	2.4	1.28	1.4E-03	6	32.9	6,7-dimethyl-8-ribityllumazine synthase (DMRL synthase)
CAL0000706	orf19.94	C6_00930C_A	orf19.94	2.4	1.27	4.3E-02	6	22.7	Uncharacterized protein
CAL0001962	orf19.4477	C1_04020C_A	CSH1	2.4	1.27	1.0E-05	16	47.1	Csh1p
CAL0004923	orf19.386	C1_08410C_A	SAM4	2.4	1.27	2.4E-02	5	18.4	S-adenosylmethionine-homocysteine S-methyltransferase
CAL0004024	orf19.7296	CR_08990C_A	CAALFM	2.4	1.26	9.6E-03	9	24.1	Uncharacterized protein
CAL0004557	orf19.4777	C1_09190C_A	DAK2	2.4	1.23	5.3E-08	24	47.9	Dihydroxyacetone kinase
CAL0004916	orf19.382	C1_08380W_A	TEF2	2.3	1.22	1.1E-02	16	52.2	Elongation factor 1-alpha 2 (EF-1-alpha 2)
CAL0004253	orf19.2762	C4_02410C_A	AHP1	2.3	1.21	1.1E-03	6	58.1	Thioredoxin peroxidase
CAL0002168	orf19.7080	C7_00400W_A	LEU2	2.2	1.14	4.9E-02	8	20.5	3-isopropylmalate dehydrogenase (EC 1.1.1.85)
CAL0002678	orf19.822	C2_04010C_A	HSP21	2.2	1.13	3.3E-05	8	45.6	Small heat shock protein 21
CAL0000101	orf19.4216	C5_02110W_A	HSP12	2.2	1.11	2.3E-10	9	57.1	Hsp12p
CAL0001960	orf19.4476	C1_04010C_A	orf19.4476	2.1	1.09	4.9E-03	14	40.5	Uncharacterized protein
CAL0004002	orf19.251	C3_02610C_A	GLX3	2.1	1.09	7.3E-05	9	57.4	Glyoxalase 3 (EC 4.2.1.130)
CAL0002358	orf19.3160	C5_02080C_A	HSP12	2.1	1.09	1.3E-10	9	55.6	Hsp12p (Lipid-binding protein)
CAF0007466	orf19.4674.1	C4_01160W_A	CRD2	2.1	1.06	3.5E-07	3	59.2	Crd2p
CAL0001761	orf19.5078	C1_08060W_A	OFR1	2.1	1.04	3.9E-02	5	33.5	Ofr1p
CAL0005353	orf19.4833	C1_09690W_A	MLS1	2.1	1.03	1.9E-05	17	33.6	Malate synthase (EC 2.3.3.9)
CAL0003500	orf19.5211	C2_05890C_A	IDP1	1.9	0.94	4.3E-02	21	43.6	Isocitrate dehydrogenase [NADP] (EC 1.1.1.42)
CAL0003630	orf19.925	C5_00640C_A	SAM51	1.9	0.92	1.6E-03	9	17.2	Sam51p
CAL0005475	orf19.2192	C2_07900W_A	GDH2	1.9	0.91	4.6E-03	33	33.8	NAD-specific glutamate dehydrogenase (EC 1.4.1.2)
CAL0003649	orf19.5285	CR_05390W_A	PST3	1.9	0.90	2.3E-06	7	55.6	Flavodoxin-like fold family protein

Supplementary Figure 1: Preparation of *C. albicans* protein samples from unstressed and salt-stressed cells. Four replicate samples were prepared for each condition (Samples 1-4), the first three of which were used for the proteomic analyses described in the main text. Each protein sample was prepared by pooling the extracts generated from the cells after five sequential rounds of bead beating to ensure that efficient cell lysis had occurred. Before pooling, each of the five extracts was analysed by gel electrophoresis (1st to 5th ex). The first extraction (1st ex, *) for Samples 1-3 are shown in Figure 1.

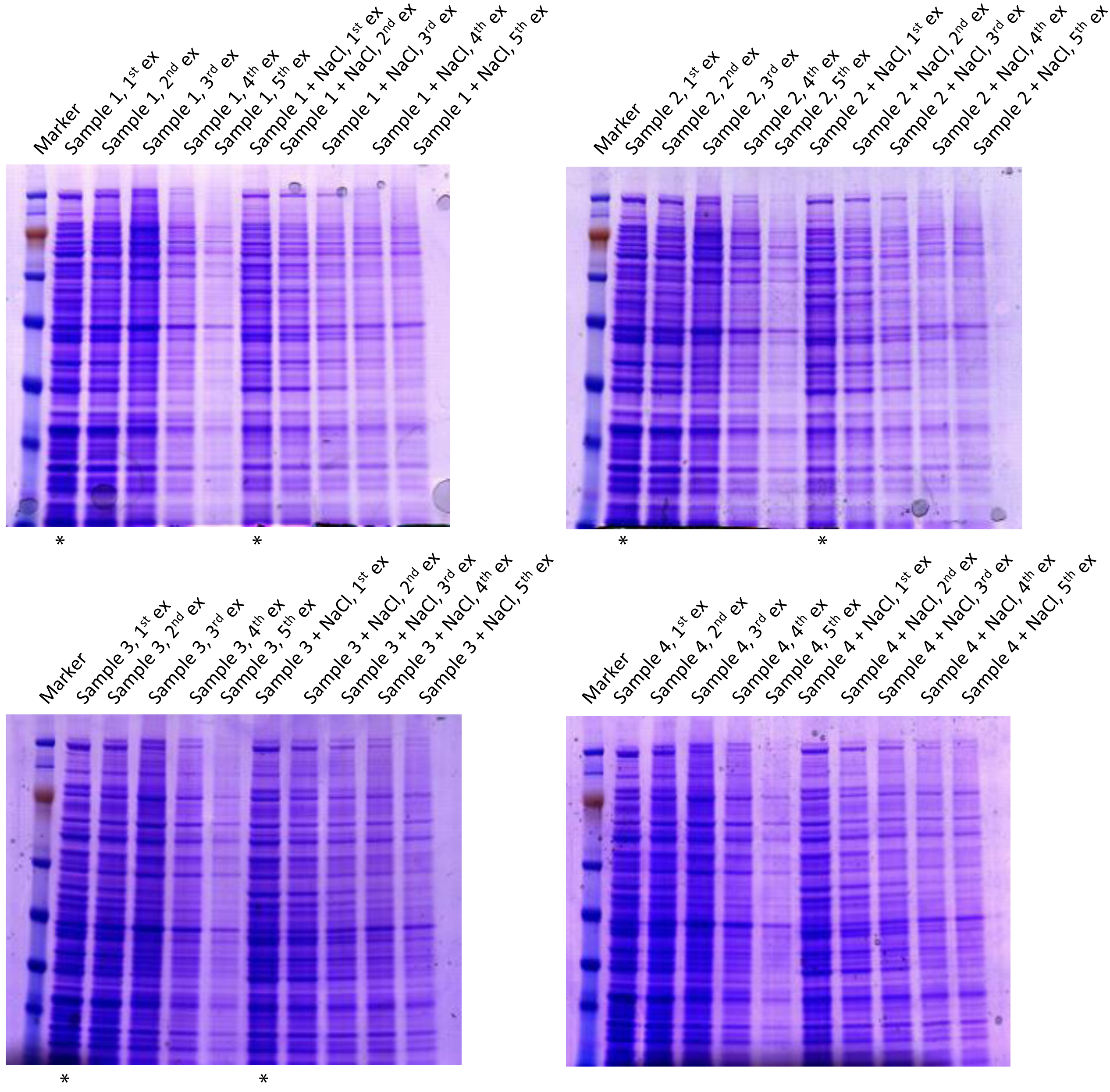

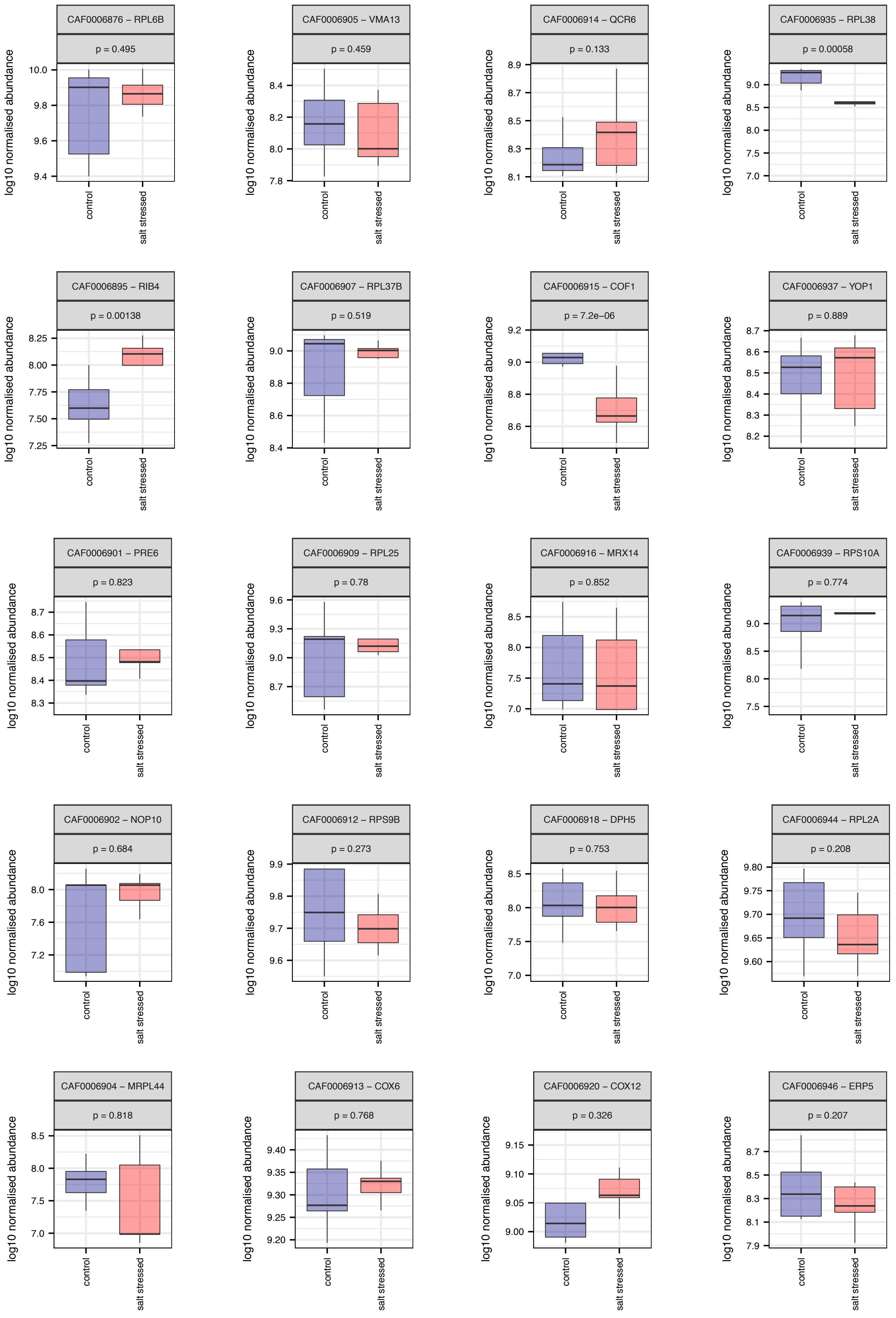
CAF0006876 – RPL6B
p = 0.495
CAF0006905 – VMA13
p = 0.459
CAF0006914 – QCR6
p = 0.133
CAF0006935 – RPL38
p = 0.00058
CAF0006895 – RIB4
p = 0.00138
CAF0006907 – RPL37B
p = 0.519
CAF0006915 – COF1
p = 7.2e–06
CAF0006937 – YOP1
p = 0.889
CAF0006901 – PRE6
p = 0.823
CAF0006909 – RPL25
p = 0.78
CAF0006916 – MRX14
p = 0.852
CAF0006939 – RPS10A
p = 0.774
CAF0006902 – NOP10
p = 0.684
CAF0006912 – RPS9B
p = 0.273
CAF0006918 – DPH5
p = 0.753
CAF0006944 – RPL2A
p = 0.208
CAF0006904 – MRPL44
p = 0.818
CAF0006913 – COX6
p = 0.768
CAF0006920 – COX12
p = 0.326
CAF0006946 – ERP5
p = 0.207
log10 normalised abundance
control
salt stressed

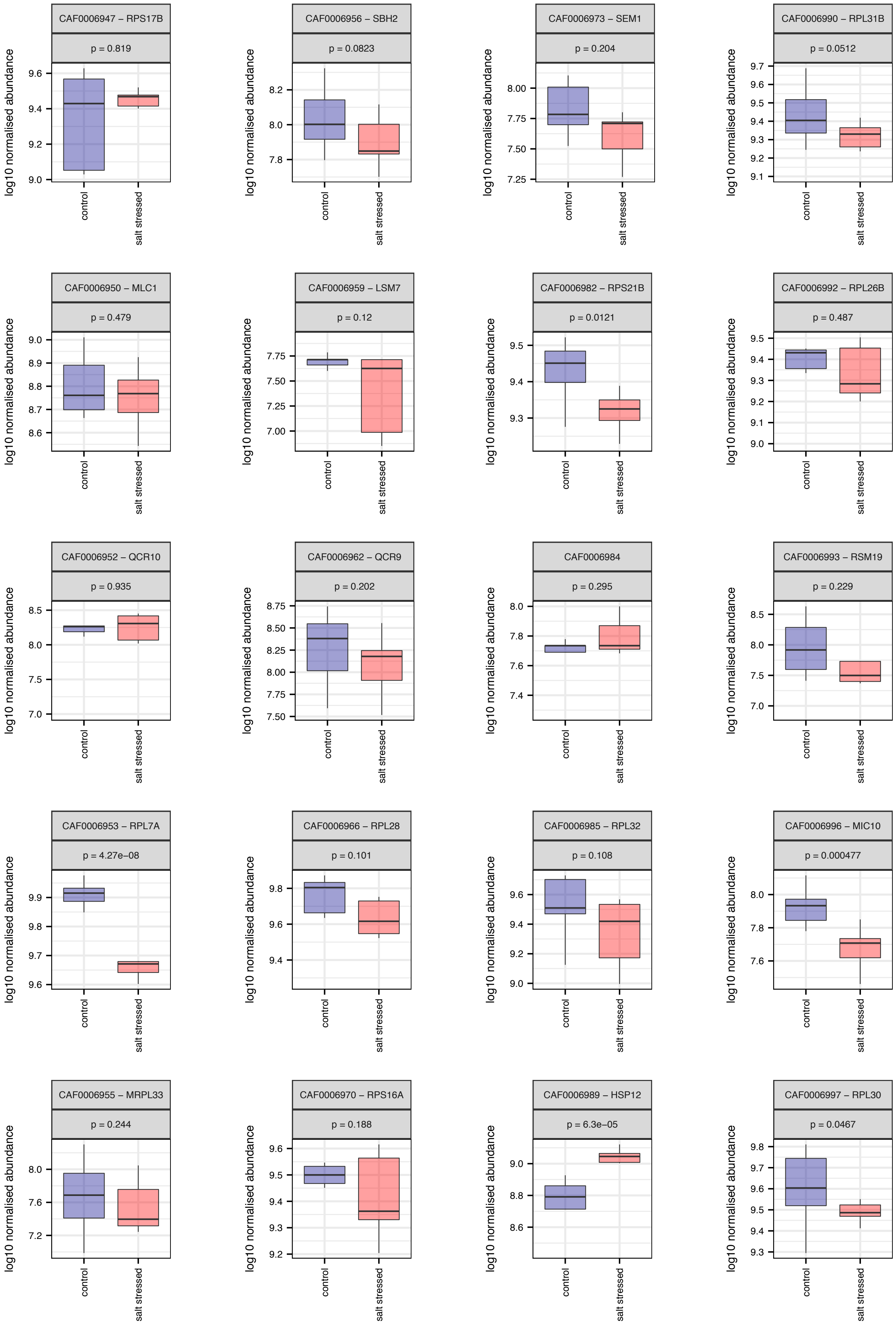
CAF0006947 – RPS17B
p = 0.819
CAF0006956 – SBH2
p = 0.0823
CAF0006973 – SEM1
p = 0.204
CAF0006990 – RPL31B
p = 0.0512
CAF0006950 – MLC1
p = 0.479
CAF0006959 – LSM7
p = 0.12
CAF0006982 – RPS21B
p = 0.0121
CAF0006992 – RPL26B
p = 0.487
CAF0006952 – QCR10
p = 0.935
CAF0006962 – QCR9
p = 0.202
CAF0006984
p = 0.295
CAF0006993 – RSM19
p = 0.229
CAF0006953 – RPL7A
p = 4.27e–08
CAF0006966 – RPL28
p = 0.101
CAF0006985 – RPL32
p = 0.108
CAF0006996 – MIC10
p = 0.000477
CAF0006955 – MRPL33
p = 0.244
CAF0006970 – RPS16A
p = 0.188
CAF0006989 – HSP12
p = 6.3e–05
CAF0006997 – RPL30
p = 0.0467
log10 normalised abundance
control
salt stressed

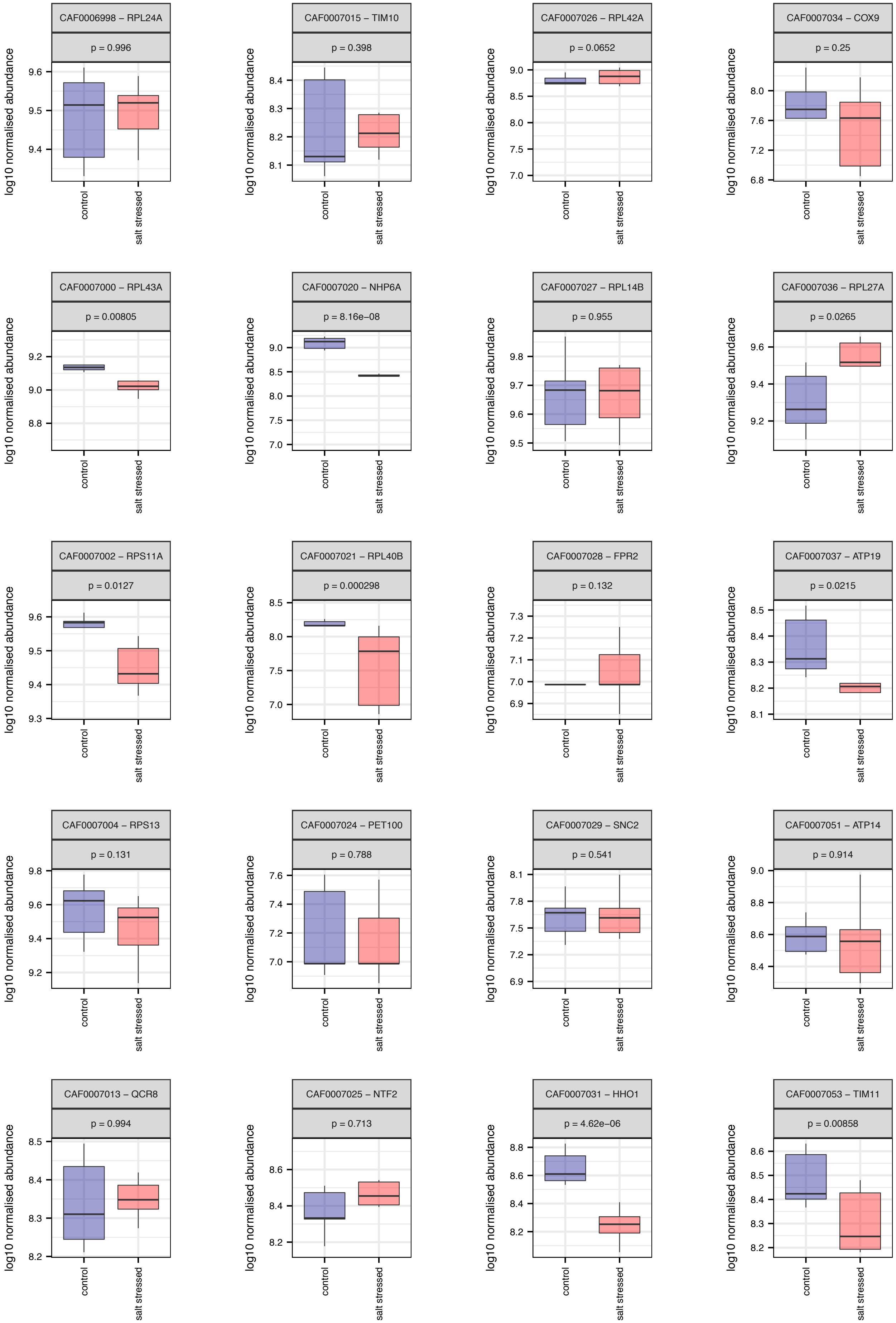
CAF0006998 – RPL24A
p = 0.996
CAF0007015 – TIM10
p = 0.398
CAF0007026 – RPL42A
p = 0.0652
CAF0007034 – COX9
p = 0.25
CAF0007000 – RPL43A
p = 0.00805
CAF0007020 – NHP6A
p = 8.16e–08
CAF0007027 – RPL14B
p = 0.955
CAF0007036 – RPL27A
p = 0.0265
CAF0007002 – RPS11A
p = 0.0127
CAF0007021 – RPL40B
p = 0.000298
CAF0007028 – FPR2
p = 0.132
CAF0007037 – ATP19
p = 0.0215
CAF0007004 – RPS13
p = 0.131
CAF0007024 – PET100
p = 0.788
CAF0007029 – SNC2
p = 0.541
CAF0007051 – ATP14
p = 0.914
CAF0007013 – QCR8
p = 0.994
CAF0007025 – NTF2
p = 0.713
CAF0007031 – HHO1
p = 4.62e–06
CAF0007053 – TIM11
p = 0.00858
log10 normalised abundance
control
salt stressed

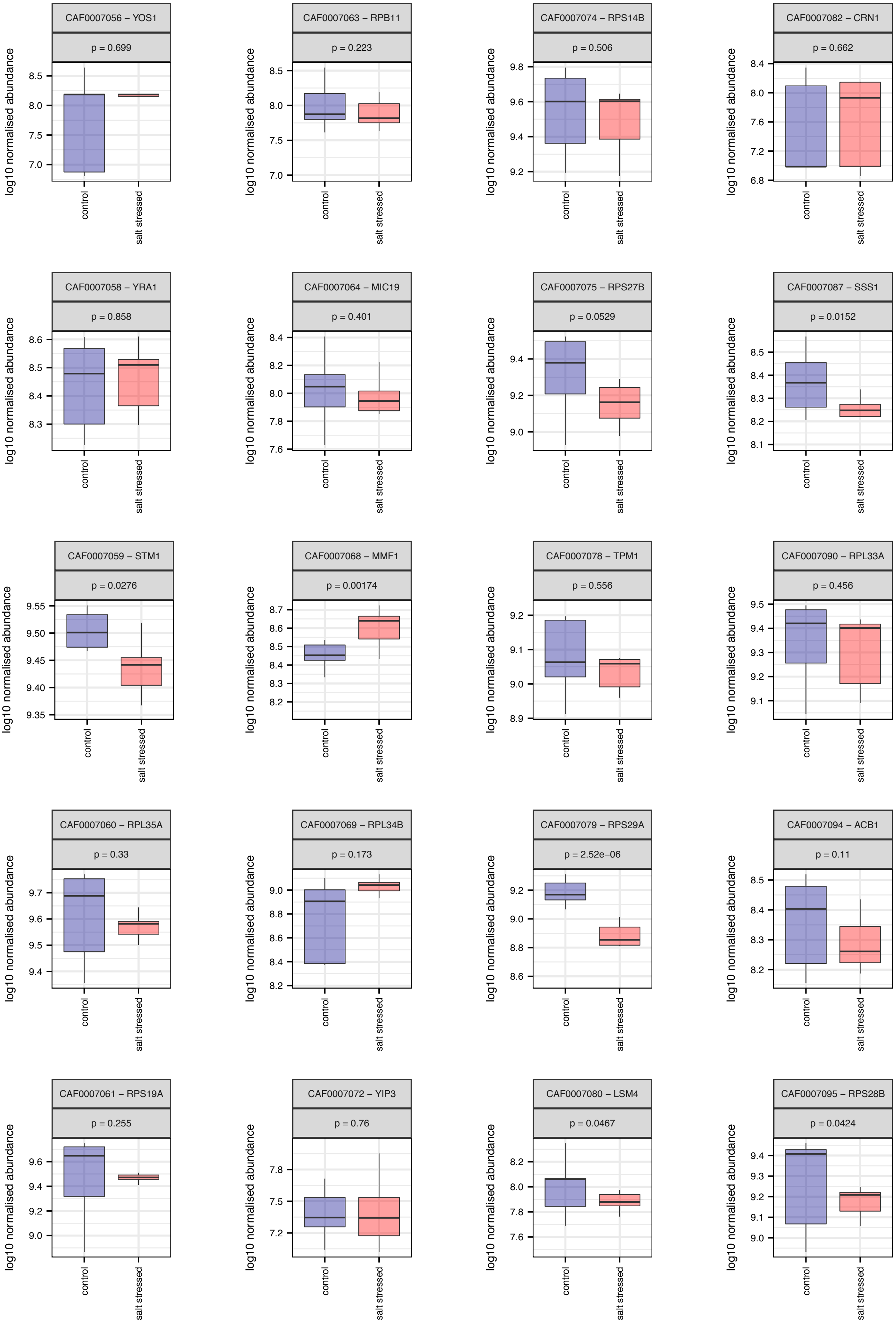
CAF0007056 – YOS1
p = 0.699
CAF0007063 – RPB11
p = 0.223
CAF0007074 – RPS14B
p = 0.506
CAF0007082 – CRN1
p = 0.662
CAF0007058 – YRA1
p = 0.858
CAF0007064 – MIC19
p = 0.401
CAF0007075 – RPS27B
p = 0.0529
CAF0007087 – SSS1
p = 0.0152
CAF0007059 – STM1
p = 0.0276
CAF0007068 – MMF1
p = 0.00174
CAF0007078 – TPM1
p = 0.556
CAF0007090 – RPL33A
p = 0.456
CAF0007060 – RPL35A
p = 0.33
CAF0007069 – RPL34B
p = 0.173
CAF0007079 – RPS29A
p = 2.52e–06
CAF0007094 – ACB1
p = 0.11
CAF0007061 – RPS19A
p = 0.255
CAF0007072 – YIP3
p = 0.76
CAF0007080 – LSM4
p = 0.0467
CAF0007095 – RPS28B
p = 0.0424
log10 normalised abundance
control
salt stressed

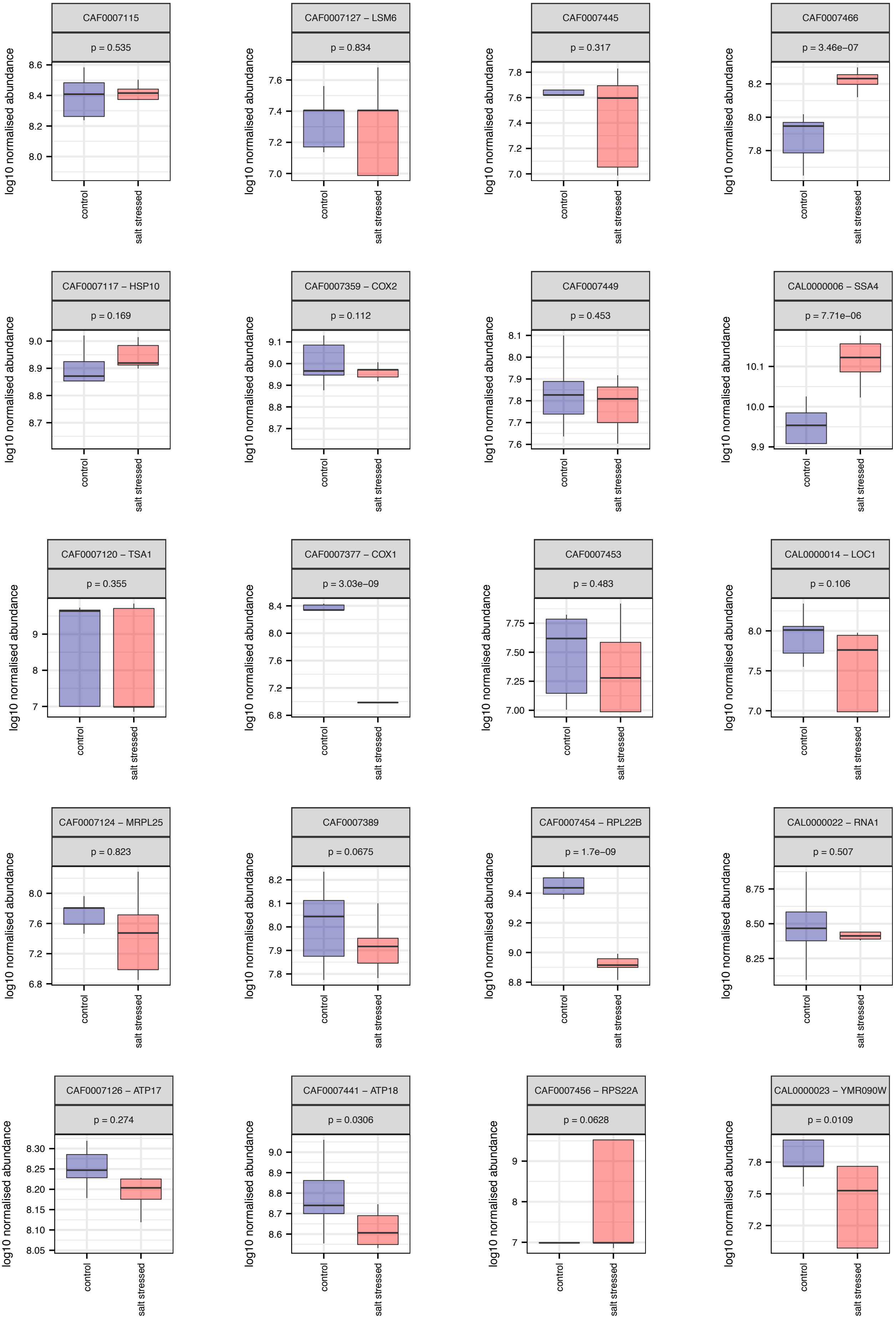
CAF0007115
p = 0.535
CAF0007127 – LSM6
p = 0.834
CAF0007445
p = 0.317
CAF0007466
p = 3.46e–07
CAF0007117 – HSP10
p = 0.169
CAF0007359 – COX2
p = 0.112
CAF0007449
p = 0.453
CAL0000006 – SSA4
p = 7.71e–06
CAF0007120 – TSA1
p = 0.355
CAF0007377 – COX1
p = 3.03e–09
CAF0007453
p = 0.483
CAL0000014 – LOC1
p = 0.106
CAF0007124 – MRPL25
p = 0.823
CAF0007389
p = 0.0675
CAF0007454 – RPL22B
p = 1.7e–09
CAL0000022 – RNA1
p = 0.507
CAF0007126 – ATP17
p = 0.274
CAF0007441 – ATP18
p = 0.0306
CAF0007456 – RPS22A
p = 0.0628
CAL0000023 – YMR090W
p = 0.0109
log10 normalised abundance
control
salt stressed

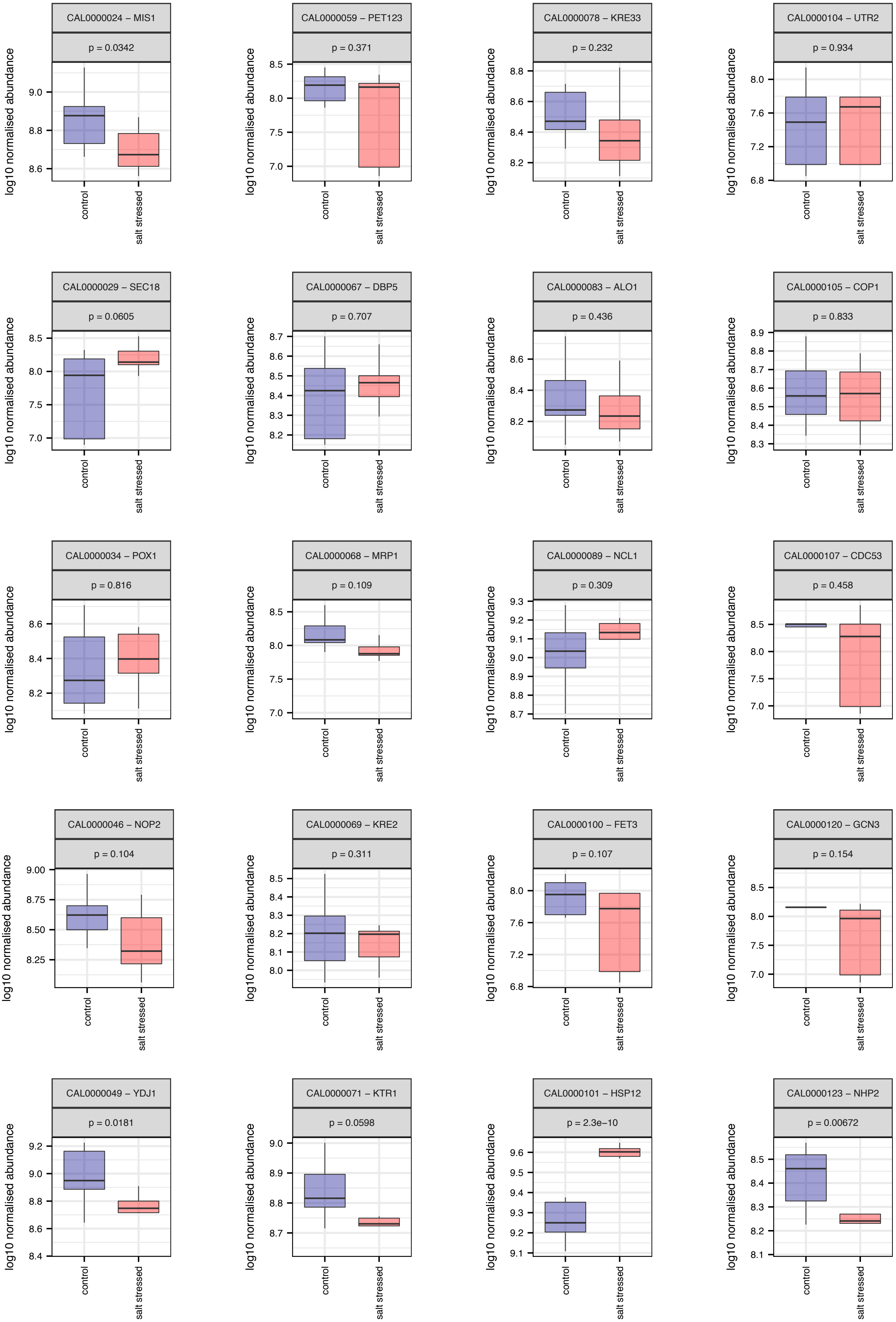
CAL0000024 – MIS1
p = 0.0342
CAL0000059 – PET123
p = 0.371
CAL0000078 – KRE33
p = 0.232
CAL0000104 – UTR2
p = 0.934
CAL0000029 – SEC18
p = 0.0605
CAL0000067 – DBP5
p = 0.707
CAL0000083 – ALO1
p = 0.436
CAL0000105 – COP1
p = 0.833
CAL0000034 – POX1
p = 0.816
CAL0000068 – MRP1
p = 0.109
CAL0000089 – NCL1
p = 0.309
CAL0000107 – CDC53
p = 0.458
CAL0000046 – NOP2
p = 0.104
CAL0000069 – KRE2
p = 0.311
CAL0000100 – FET3
p = 0.107
CAL0000120 – GCN3
p = 0.154
CAL0000049 – YDJ1
p = 0.0181
CAL0000071 – KTR1
p = 0.0598
CAL0000101 – HSP12
p = 2.3e–10
CAL0000123 – NHP2
p = 0.00672
log10 normalised abundance
control
salt stressed

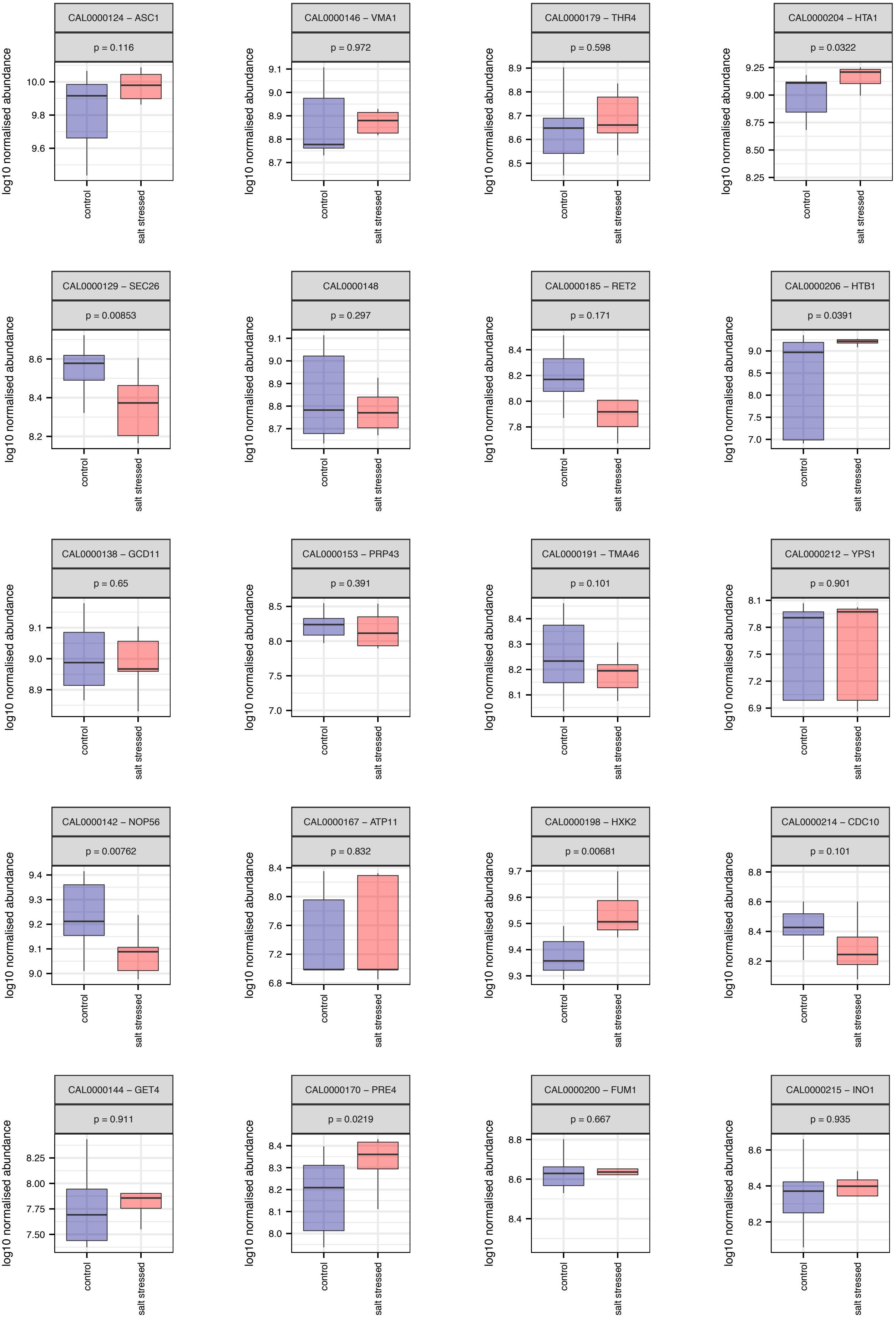
CAL0000124 – ASC1
p = 0.116
CAL0000146 – VMA1
p = 0.972
CAL0000179 – THR4
p = 0.598
CAL0000204 – HTA1
p = 0.0322
CAL0000129 – SEC26
p = 0.00853
CAL0000148
p = 0.297
CAL0000185 – RET2
p = 0.171
CAL0000206 – HTB1
p = 0.0391
CAL0000138 – GCD11
p = 0.65
CAL0000153 – PRP43
p = 0.391
CAL0000191 – TMA46
p = 0.101
CAL0000212 – YPS1
p = 0.901
CAL0000142 – NOP56
p = 0.00762
CAL0000167 – ATP11
p = 0.832
CAL0000198 – HXK2
p = 0.00681
CAL0000214 – CDC10
p = 0.101
CAL0000144 – GET4
p = 0.911
CAL0000170 – PRE4
p = 0.0219
CAL0000200 – FUM1
p = 0.667
CAL0000215 – INO1
p = 0.935
log10 normalised abundance
control
salt stressed

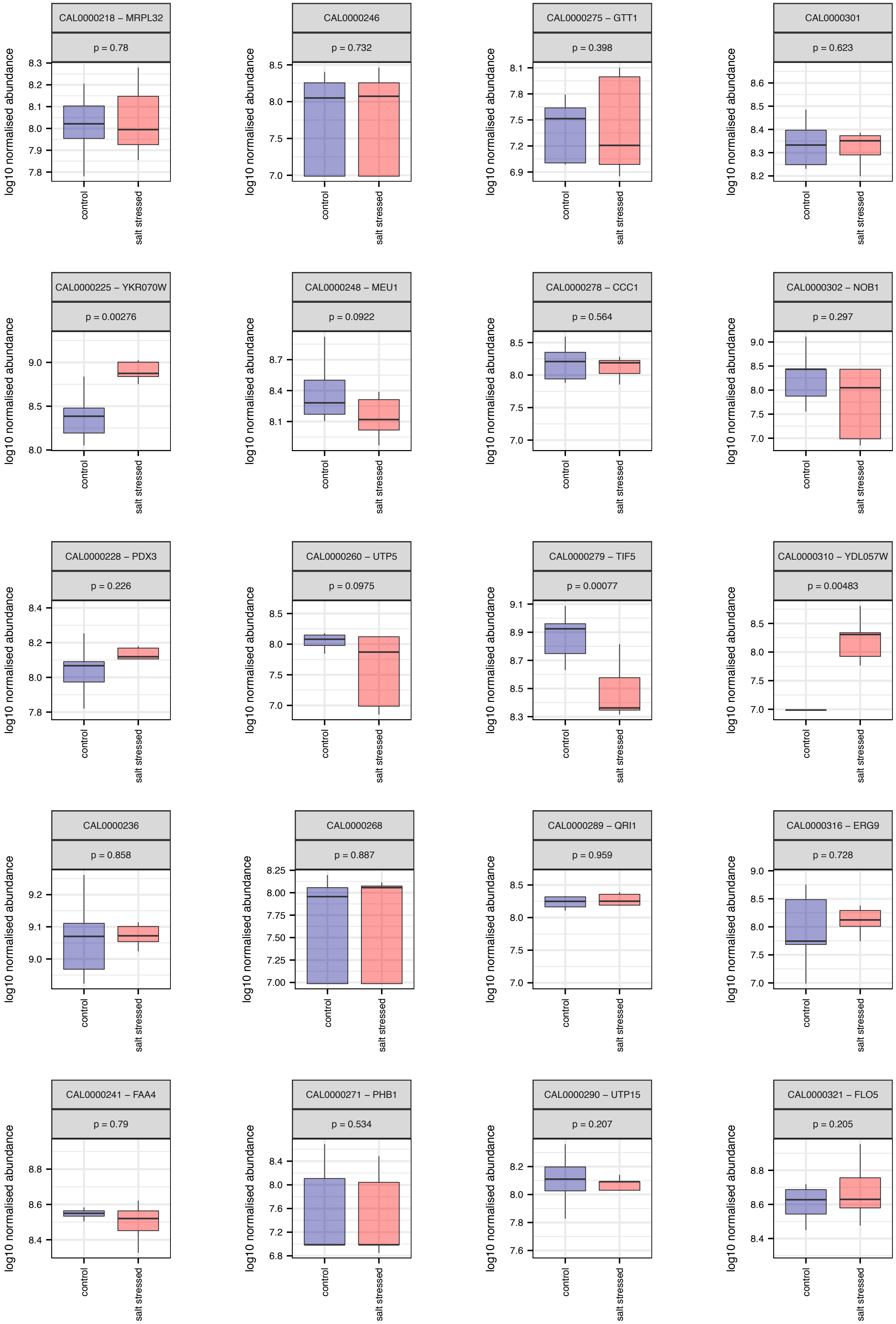
CAL0000218 – MRPL32
p = 0.78
CAL0000246
p = 0.732
CAL0000275 – GTT1
p = 0.398
CAL0000301
p = 0.623
CAL0000225 – YKR070W
p = 0.00276
CAL0000248 – MEU1
p = 0.0922
CAL0000278 – CCC1
p = 0.564
CAL0000302 – NOB1
p = 0.297
CAL0000228 – PDX3
p = 0.226
CAL0000260 – UTP5
p = 0.0975
CAL0000279 – TIF5
p = 0.00077
CAL0000310 – YDL057W
p = 0.00483
CAL0000236
p = 0.858
CAL0000268
p = 0.887
CAL0000289 – QRI1
p = 0.959
CAL0000316 – ERG9
p = 0.728
CAL0000241 – FAA4
p = 0.79
CAL0000271 – PHB1
p = 0.534
CAL0000290 – UTP15
p = 0.207
CAL0000321 – FLO5
p = 0.205
log10 normalised abundance
control
salt stressed

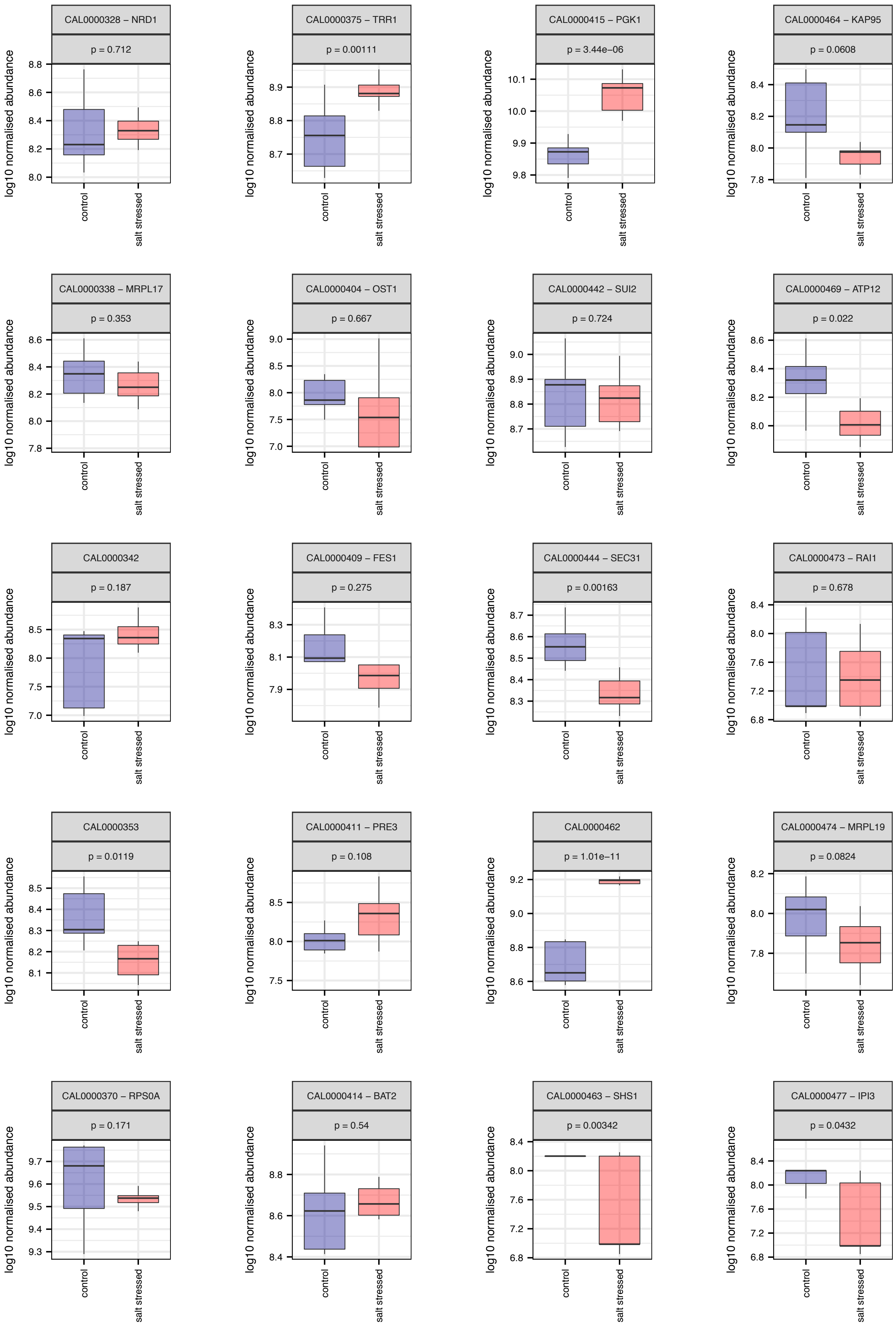
CAL0000328 – NRD1
p = 0.712
CAL0000375 – TRR1
p = 0.00111
CAL0000415 – PGK1
p = 3.44e−06
CAL0000464 – KAP95
p = 0.0608
CAL0000338 – MRPL17
p = 0.353
CAL0000404 – OST1
p = 0.667
CAL0000442 – SUI2
p = 0.724
CAL0000469 – ATP12
p = 0.022
CAL0000342
p = 0.187
CAL0000409 – FES1
p = 0.275
CAL0000444 – SEC31
p = 0.00163
CAL0000473 – RAI1
p = 0.678
CAL0000353
p = 0.0119
CAL0000411 – PRE3
p = 0.108
CAL0000462
p = 1.01e−11
CAL0000474 – MRPL19
p = 0.0824
CAL0000370 – RPS0A
p = 0.171
CAL0000414 – BAT2
p = 0.54
CAL0000463 – SHS1
p = 0.00342
CAL0000477 – IPI3
p = 0.0432
log10 normalised abundance
control
salt stressed

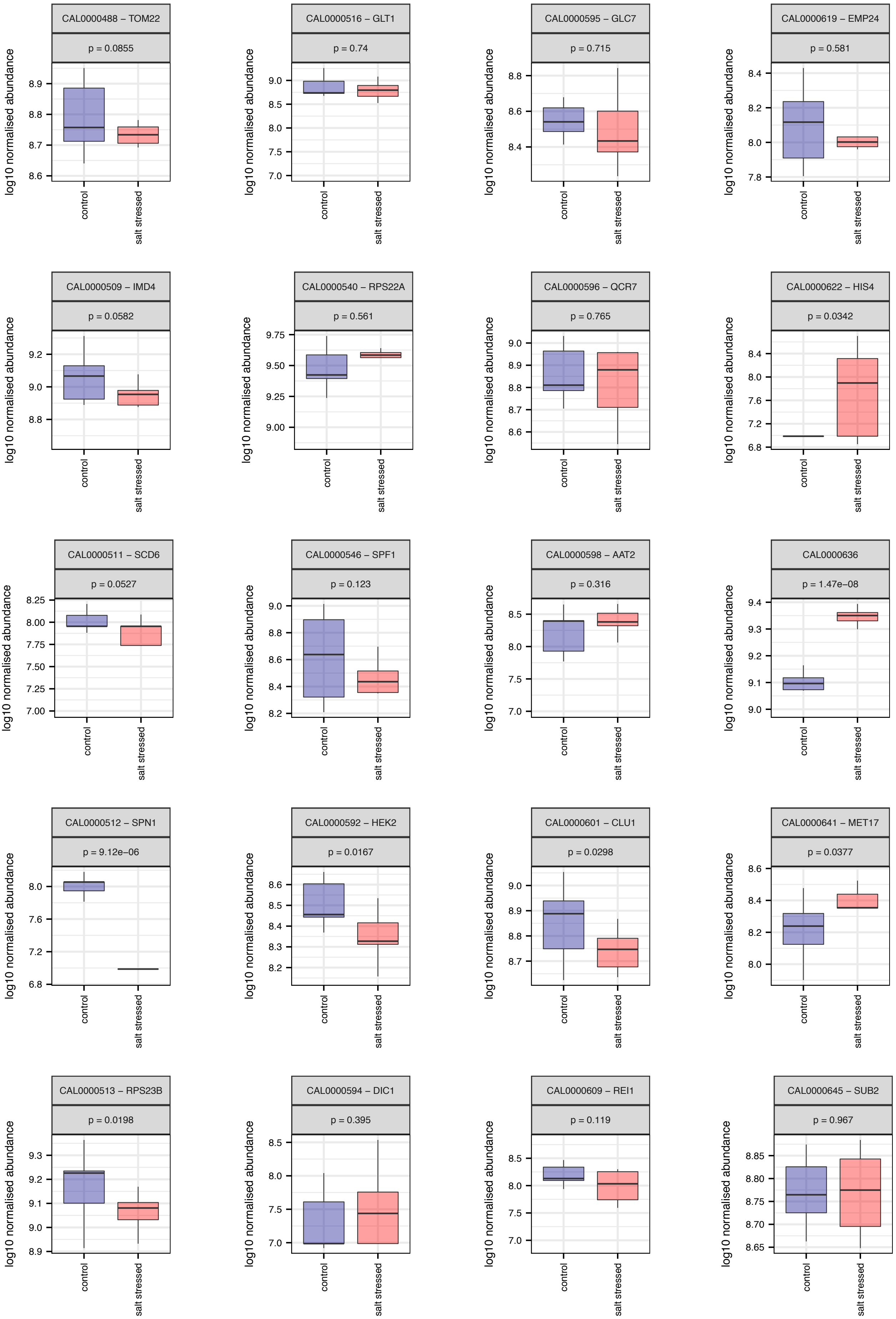
CAL0000488 – TOM22
p = 0.0855
CAL0000516 – GLT1
p = 0.74
CAL0000595 – GLC7
p = 0.715
CAL0000619 – EMP24
p = 0.581
CAL0000509 – IMD4
p = 0.0582
CAL0000540 – RPS22A
p = 0.561
CAL0000596 – QCR7
p = 0.765
CAL0000622 – HIS4
p = 0.0342
CAL0000511 – SCD6
p = 0.0527
CAL0000546 – SPF1
p = 0.123
CAL0000598 – AAT2
p = 0.316
CAL0000636
p = 1.47e−08
CAL0000512 – SPN1
p = 9.12e−06
CAL0000592 – HEK2
p = 0.0167
CAL0000601 – CLU1
p = 0.0298
CAL0000641 – MET17
p = 0.0377
CAL0000513 – RPS23B
p = 0.0198
CAL0000594 – DIC1
p = 0.395
CAL0000609 – REI1
p = 0.119
CAL0000645 – SUB2
p = 0.967
log10 normalised abundance
control
salt stressed

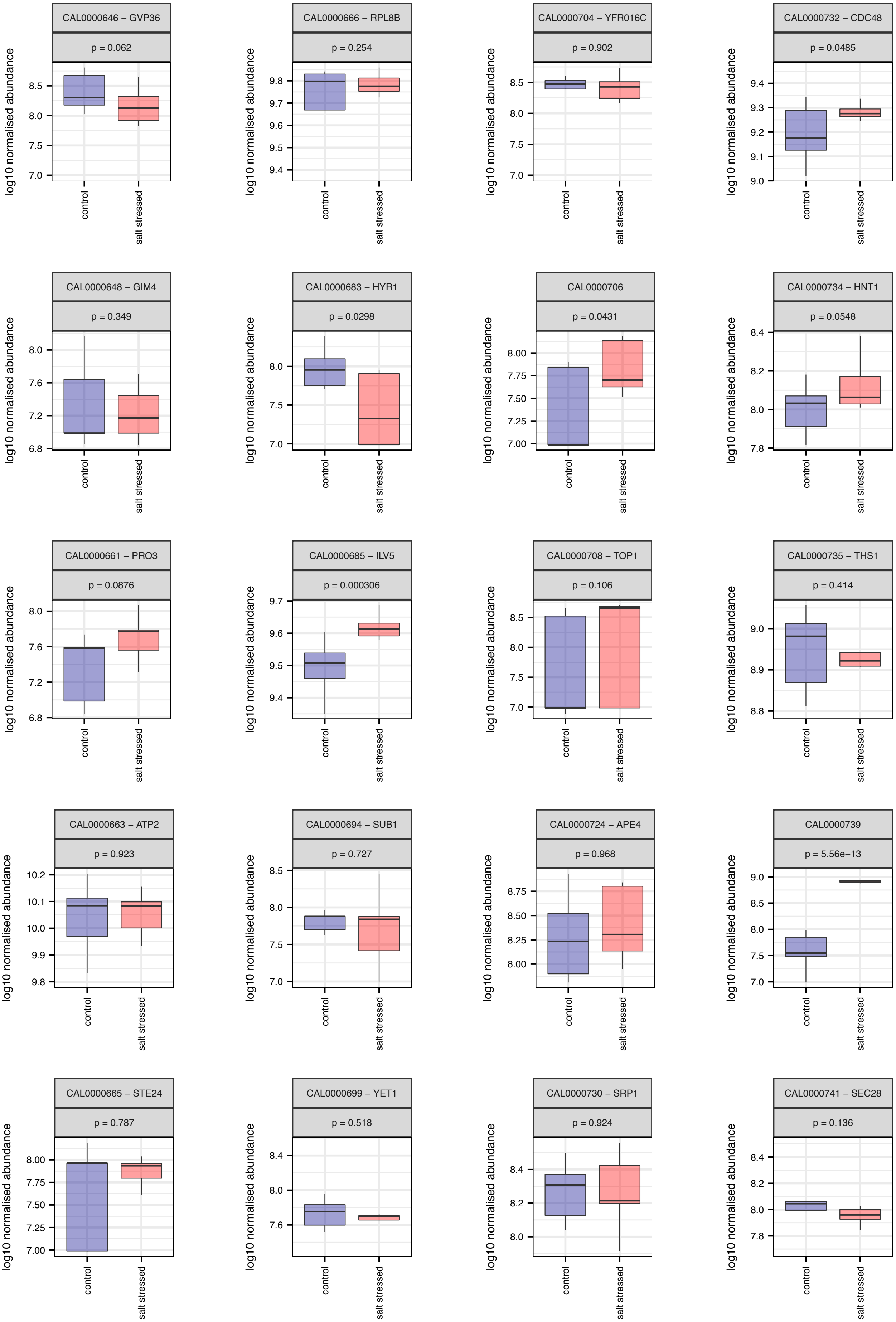
CAL0000646 – GVP36
p = 0.062
CAL0000666 – RPL8B
p = 0.254
CAL0000704 – YFR016C
p = 0.902
CAL0000732 – CDC48
p = 0.0485
CAL0000648 – GIM4
p = 0.349
CAL0000683 – HYR1
p = 0.0298
CAL0000706
p = 0.0431
CAL0000734 – HNT1
p = 0.0548
CAL0000661 – PRO3
p = 0.0876
CAL0000685 – ILV5
p = 0.000306
CAL0000708 – TOP1
p = 0.106
CAL0000735 – THS1
p = 0.414
CAL0000663 – ATP2
p = 0.923
CAL0000694 – SUB1
p = 0.727
CAL0000724 – APE4
p = 0.968
CAL0000739
p = 5.56e–13
CAL0000665 – STE24
p = 0.787
CAL0000699 – YET1
p = 0.518
CAL0000730 – SRP1
p = 0.924
CAL0000741 – SEC28
p = 0.136
log10 normalised abundance
control
salt stressed

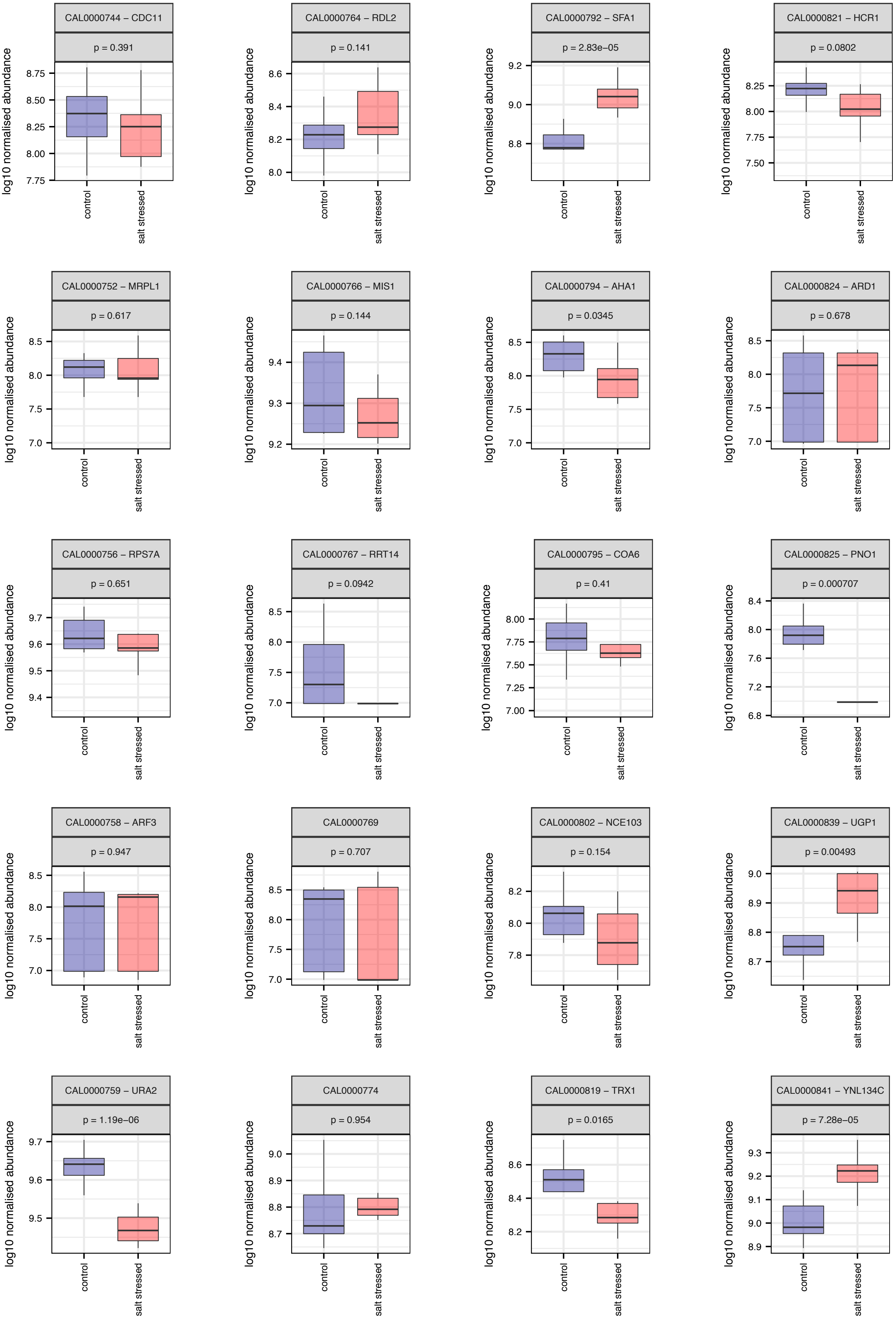
CAL0000744 – CDC11
p = 0.391
CAL0000764 – RDL2
p = 0.141
CAL0000792 – SFA1
p = 2.83e–05
CAL0000821 – HCR1
p = 0.0802
CAL0000752 – MRPL1
p = 0.617
CAL0000766 – MIS1
p = 0.144
CAL0000794 – AHA1
p = 0.0345
CAL0000824 – ARD1
p = 0.678
CAL0000756 – RPS7A
p = 0.651
CAL0000767 – RRT14
p = 0.0942
CAL0000795 – COA6
p = 0.41
CAL0000825 – PNO1
p = 0.000707
CAL0000758 – ARF3
p = 0.947
CAL0000769
p = 0.707
CAL0000802 – NCE103
p = 0.154
CAL0000839 – UGP1
p = 0.00493
CAL0000759 – URA2
p = 1.19e–06
CAL0000774
p = 0.954
CAL0000819 – TRX1
p = 0.0165
CAL0000841 – YNL134C
p = 7.28e–05
log10 normalised abundance
control
salt stressed

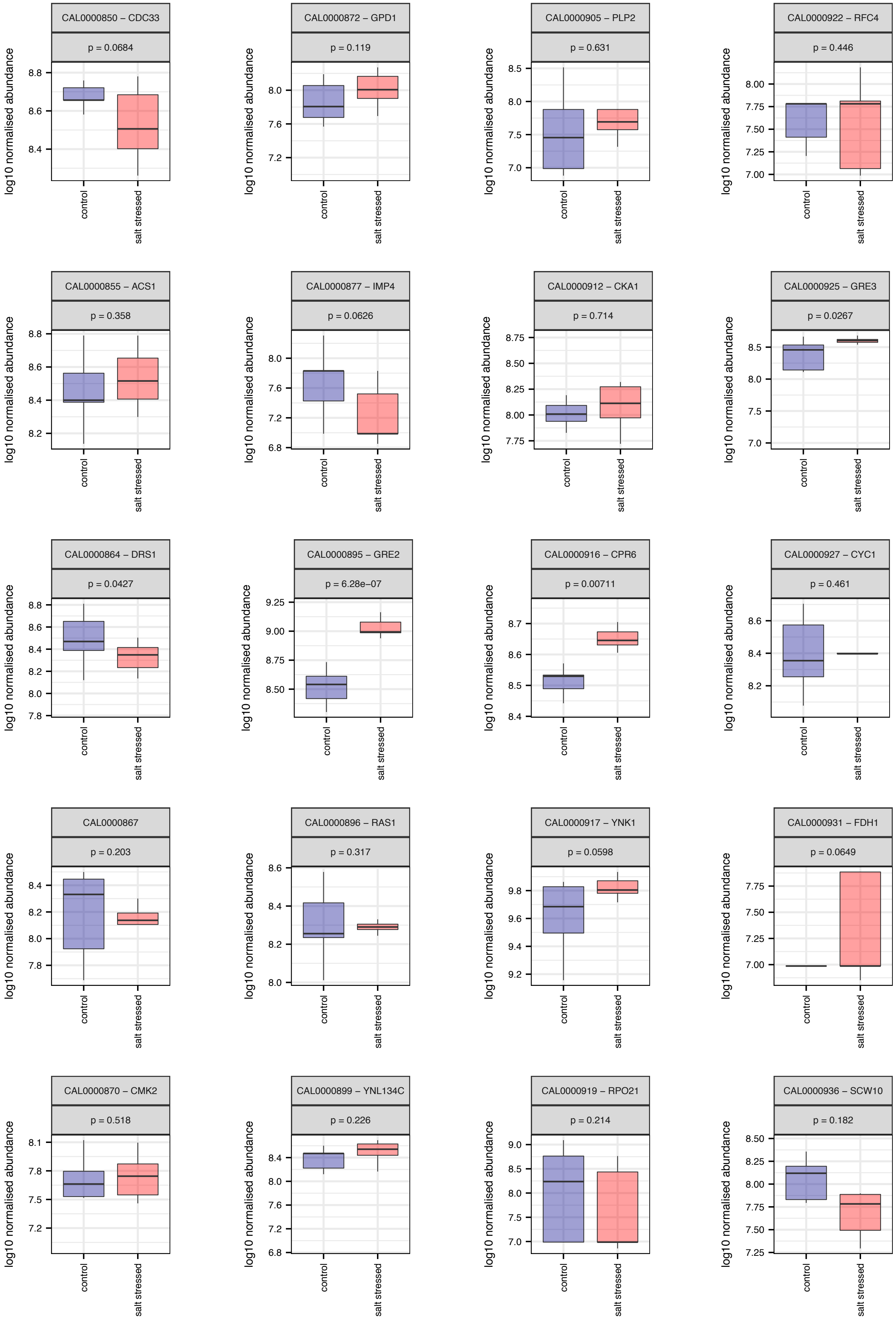
CAL0000850 – CDC33
p = 0.0684
CAL0000872 – GPD1
p = 0.119
CAL0000905 – PLP2
p = 0.631
CAL0000922 – RFC4
p = 0.446
CAL0000855 – ACS1
p = 0.358
CAL0000877 – IMP4
p = 0.0626
CAL0000912 – CKA1
p = 0.714
CAL0000925 – GRE3
p = 0.0267
CAL0000864 – DRS1
p = 0.0427
CAL0000895 – GRE2
p = 6.28e−07
CAL0000916 – CPR6
p = 0.00711
CAL0000927 – CYC1
p = 0.461
CAL0000867
p = 0.203
CAL0000896 – RAS1
p = 0.317
CAL0000917 – YNK1
p = 0.0598
CAL0000931 – FDH1
p = 0.0649
CAL0000870 – CMK2
p = 0.518
CAL0000899 – YNL134C
p = 0.226
CAL0000919 – RPO21
p = 0.214
CAL0000936 – SCW10
p = 0.182
log10 normalised abundance
control
salt stressed

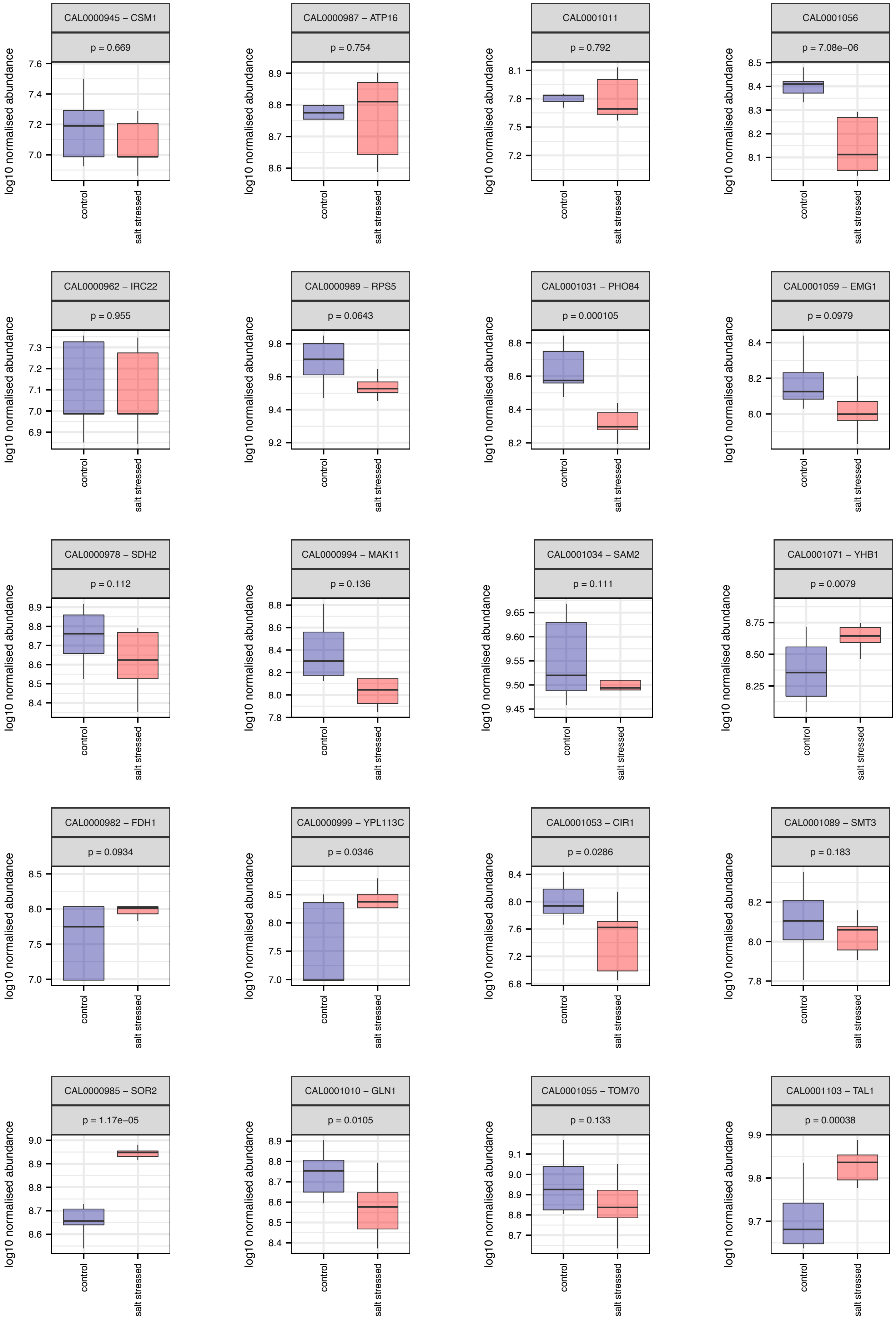
CAL0000945 – CSM1
p = 0.669
CAL0000987 – ATP16
p = 0.754
CAL0001011
p = 0.792
CAL0001056
p = 7.08e−06
CAL0000962 – IRC22
p = 0.955
CAL0000989 – RPS5
p = 0.0643
CAL0001031 – PHO84
p = 0.000105
CAL0001059 – EMG1
p = 0.0979
CAL0000978 – SDH2
p = 0.112
CAL0000994 – MAK11
p = 0.136
CAL0001034 – SAM2
p = 0.111
CAL0001071 – YHB1
p = 0.0079
CAL0000982 – FDH1
p = 0.0934
CAL0000999 – YPL113C
p = 0.0346
CAL0001053 – CIR1
p = 0.0286
CAL0001089 – SMT3
p = 0.183
CAL0000985 – SOR2
p = 1.17e−05
CAL0001010 – GLN1
p = 0.0105
CAL0001055 – TOM70
p = 0.133
CAL0001103 – TAL1
p = 0.00038
log10 normalised abundance
control
salt stressed

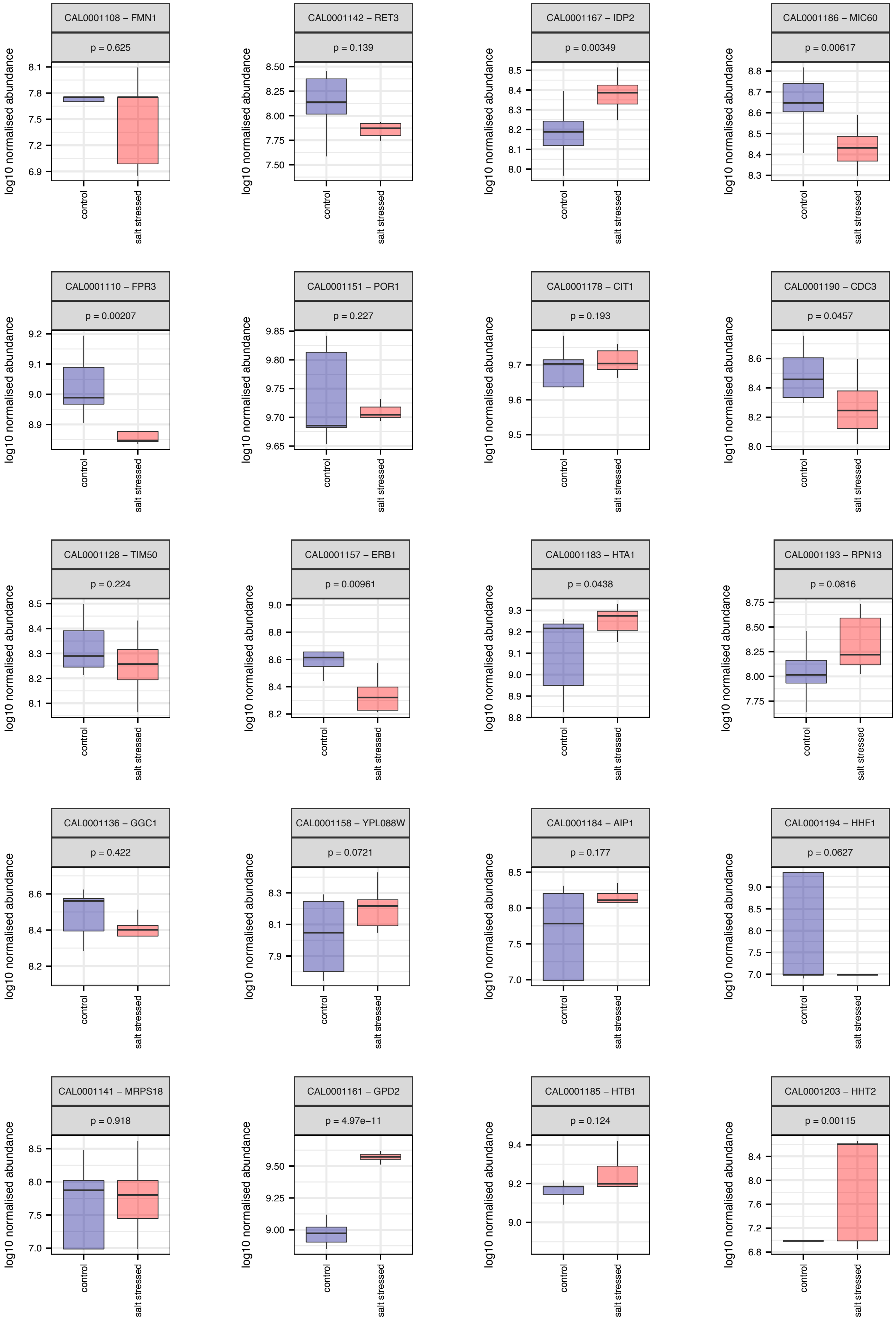
CAL0001108 – FMN1
p = 0.625
CAL0001142 – RET3
p = 0.139
CAL0001167 – IDP2
p = 0.00349
CAL0001186 – MIC60
p = 0.00617
CAL0001110 – FPR3
p = 0.00207
CAL0001151 – POR1
p = 0.227
CAL0001178 – CIT1
p = 0.193
CAL0001190 – CDC3
p = 0.0457
CAL0001128 – TIM50
p = 0.224
CAL0001157 – ERB1
p = 0.00961
CAL0001183 – HTA1
p = 0.0438
CAL0001193 – RPN13
p = 0.0816
CAL0001136 – GGC1
p = 0.422
CAL0001158 – YPL088W
p = 0.0721
CAL0001184 – AIP1
p = 0.177
CAL0001194 – HHF1
p = 0.0627
CAL0001141 – MRPS18
p = 0.918
CAL0001161 – GPD2
p = 4.97e−11
CAL0001185 – HTB1
p = 0.124
CAL0001203 – HHT2
p = 0.00115
log10 normalised abundance
control
salt stressed

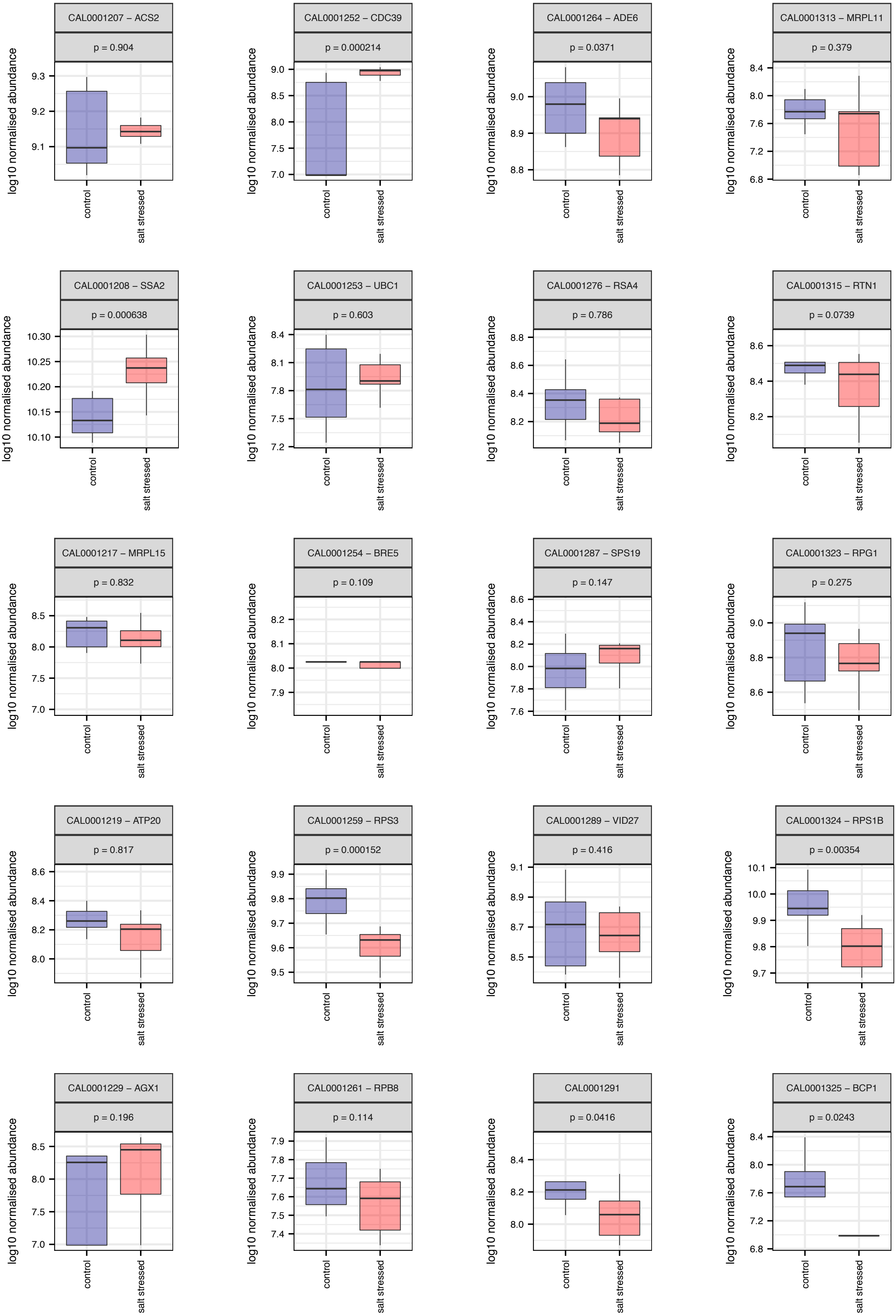
CAL0001207 – ACS2
p = 0.904
CAL0001252 – CDC39
p = 0.000214
CAL0001264 – ADE6
p = 0.0371
CAL0001313 – MRPL11
p = 0.379
CAL0001208 – SSA2
p = 0.000638
CAL0001253 – UBC1
p = 0.603
CAL0001276 – RSA4
p = 0.786
CAL0001315 – RTN1
p = 0.0739
CAL0001217 – MRPL15
p = 0.832
CAL0001254 – BRE5
p = 0.109
CAL0001287 – SPS19
p = 0.147
CAL0001323 – RPG1
p = 0.275
CAL0001219 – ATP20
p = 0.817
CAL0001259 – RPS3
p = 0.000152
CAL0001289 – VID27
p = 0.416
CAL0001324 – RPS1B
p = 0.00354
CAL0001229 – AGX1
p = 0.196
CAL0001261 – RPB8
p = 0.114
CAL0001291
p = 0.0416
CAL0001325 – BCP1
p = 0.0243
log10 normalised abundance
control
salt stressed

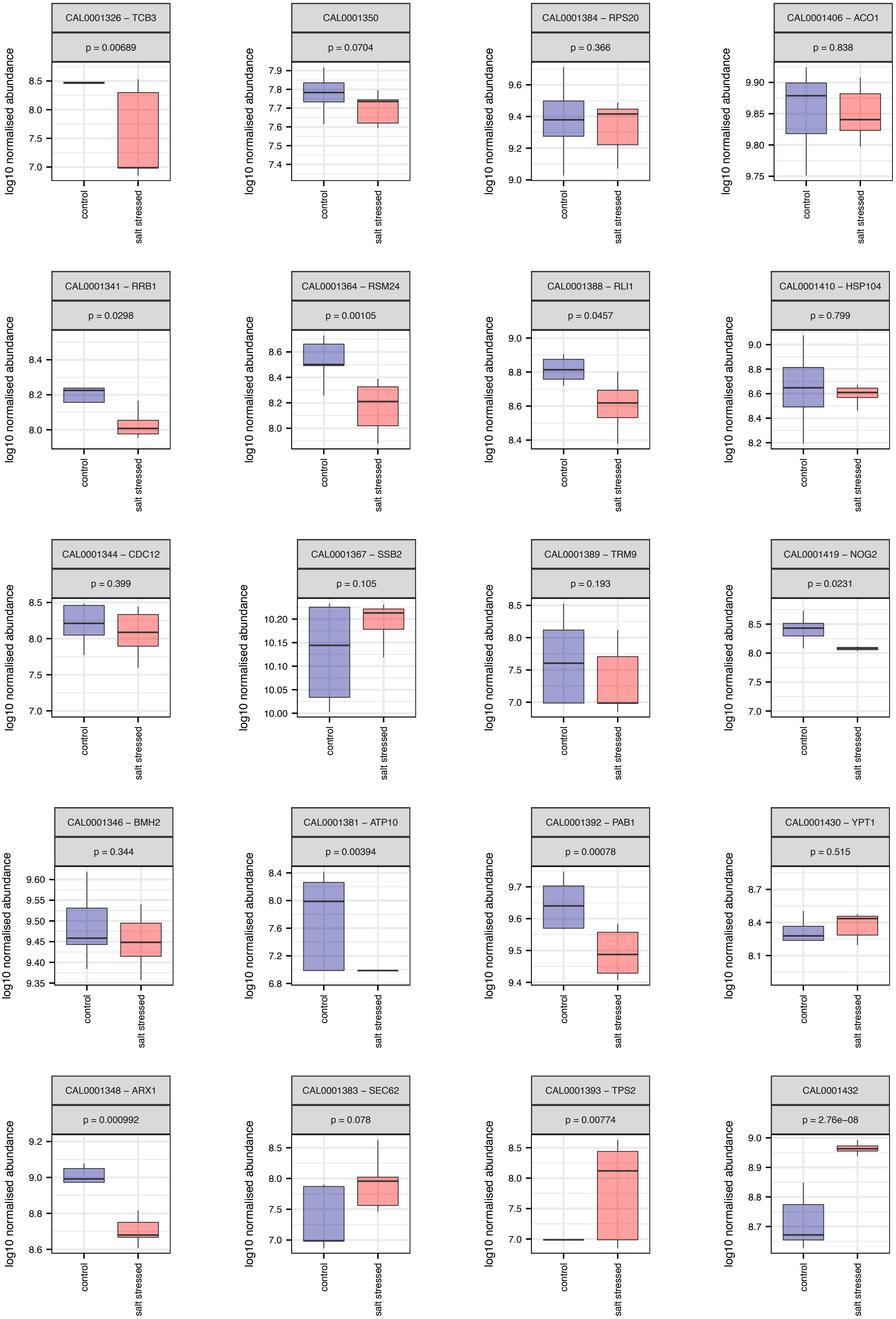
CAL0001326 – TCB3
p = 0.00689
CAL0001350
p = 0.0704
CAL0001384 – RPS20
p = 0.366
CAL0001406 – ACO1
p = 0.838
CAL0001341 – RRB1
p = 0.0298
CAL0001364 – RSM24
p = 0.00105
CAL0001388 – RLI1
p = 0.0457
CAL0001410 – HSP104
p = 0.799
CAL0001344 – CDC12
p = 0.399
CAL0001367 – SSB2
p = 0.105
CAL0001389 – TRM9
p = 0.193
CAL0001419 – NOG2
p = 0.0231
CAL0001346 – BMH2
p = 0.344
CAL0001381 – ATP10
p = 0.00394
CAL0001392 – PAB1
p = 0.00078
CAL0001430 – YPT1
p = 0.515
CAL0001348 – ARX1
p = 0.000992
CAL0001383 – SEC62
p = 0.078
CAL0001393 – TPS2
p = 0.00774
CAL0001432
p = 2.76e−08
log10 normalised abundance
control
salt stressed

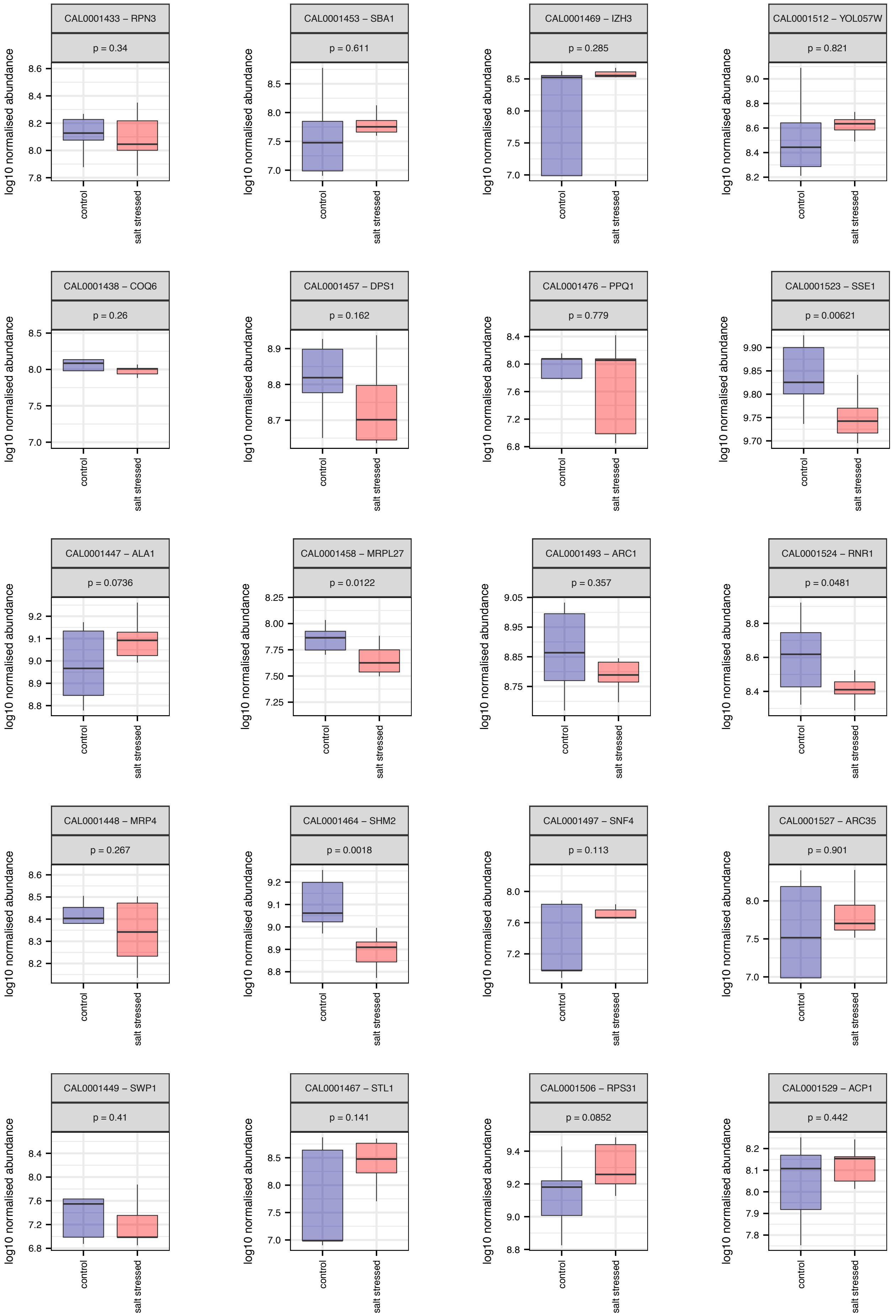
CAL0001433 – RPN3
p = 0.34
CAL0001453 – SBA1
p = 0.611
CAL0001469 – IZH3
p = 0.285
CAL0001512 – YOL057W
p = 0.821
CAL0001438 – COQ6
p = 0.26
CAL0001457 – DPS1
p = 0.162
CAL0001476 – PPQ1
p = 0.779
CAL0001523 – SSE1
p = 0.00621
CAL0001447 – ALA1
p = 0.0736
CAL0001458 – MRPL27
p = 0.0122
CAL0001493 – ARC1
p = 0.357
CAL0001524 – RNR1
p = 0.0481
CAL0001448 – MRP4
p = 0.267
CAL0001464 – SHM2
p = 0.0018
CAL0001497 – SNF4
p = 0.113
CAL0001527 – ARC35
p = 0.901
CAL0001449 – SWP1
p = 0.41
CAL0001467 – STL1
p = 0.141
CAL0001506 – RPS31
p = 0.0852
CAL0001529 – ACP1
p = 0.442
log10 normalised abundance
control
salt stressed

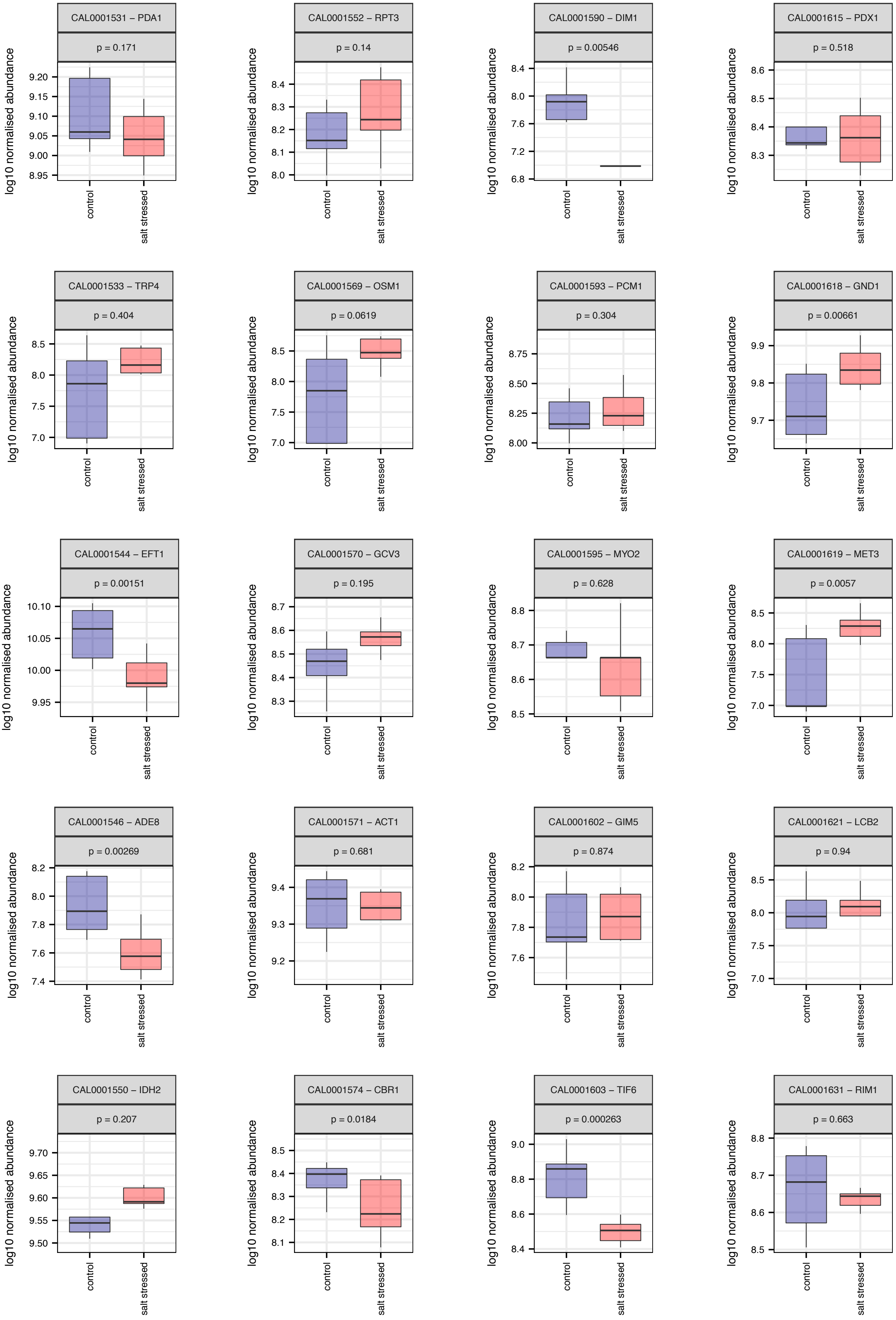
CAL0001531 – PDA1
p = 0.171
CAL0001552 – RPT3
p = 0.14
CAL0001590 – DIM1
p = 0.00546
CAL0001615 – PDX1
p = 0.518
CAL0001533 – TRP4
p = 0.404
CAL0001569 – OSM1
p = 0.0619
CAL0001593 – PCM1
p = 0.304
CAL0001618 – GND1
p = 0.00661
CAL0001544 – EFT1
p = 0.00151
CAL0001570 – GCV3
p = 0.195
CAL0001595 – MYO2
p = 0.628
CAL0001619 – MET3
p = 0.0057
CAL0001546 – ADE8
p = 0.00269
CAL0001571 – ACT1
p = 0.681
CAL0001602 – GIM5
p = 0.874
CAL0001621 – LCB2
p = 0.94
CAL0001550 – IDH2
p = 0.207
CAL0001574 – CBR1
p = 0.0184
CAL0001603 – TIF6
p = 0.000263
CAL0001631 – RIM1
p = 0.663
log10 normalised abundance
control
salt stressed

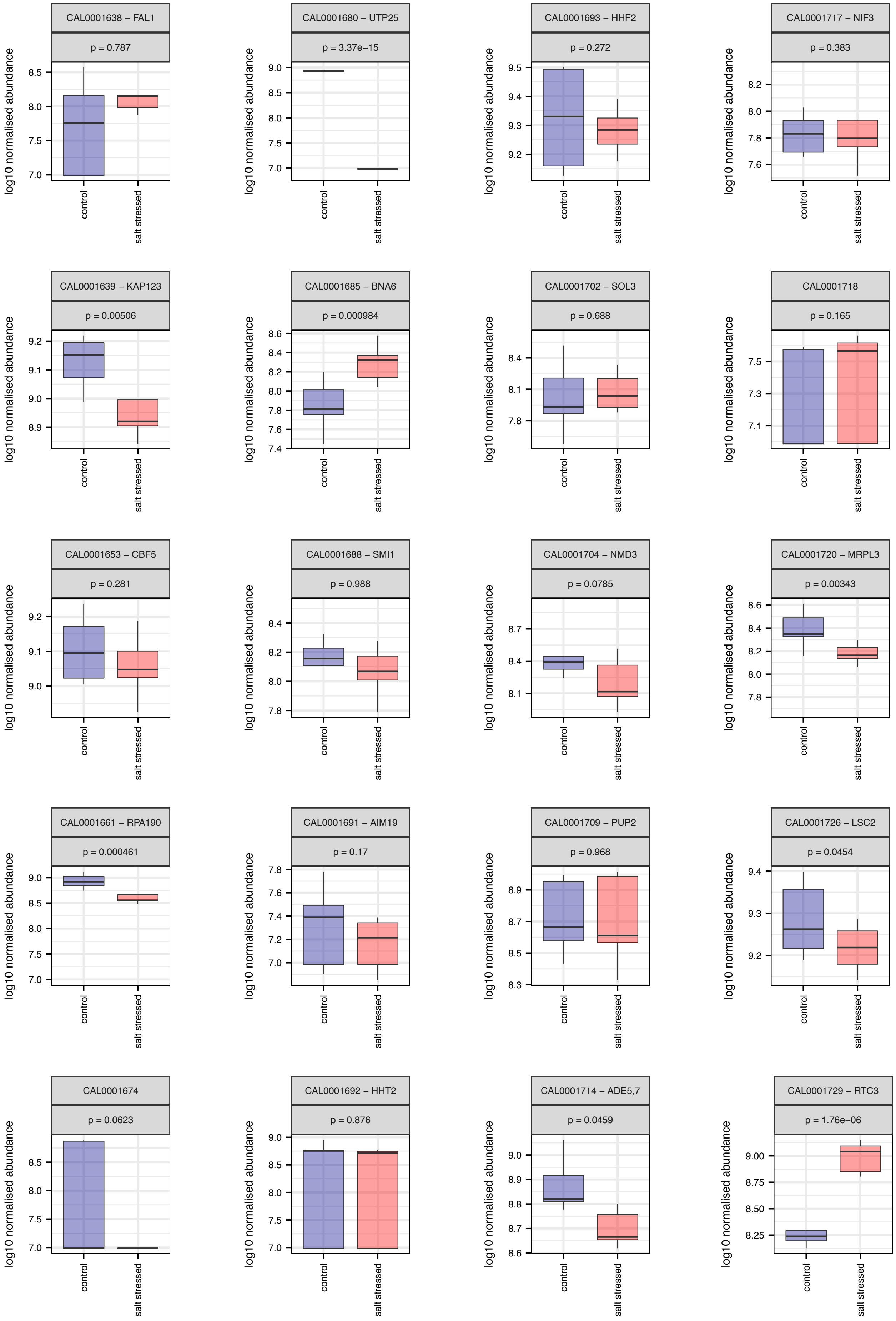
CAL0001638 – FAL1
p = 0.787
CAL0001680 – UTP25
p = 3.37e–15
CAL0001693 – HHF2
p = 0.272
CAL0001717 – NIF3
p = 0.383
CAL0001639 – KAP123
p = 0.00506
CAL0001685 – BNA6
p = 0.000984
CAL0001702 – SOL3
p = 0.688
CAL0001718
p = 0.165
CAL0001653 – CBF5
p = 0.281
CAL0001688 – SMI1
p = 0.988
CAL0001704 – NMD3
p = 0.0785
CAL0001720 – MRPL3
p = 0.00343
CAL0001661 – RPA190
p = 0.000461
CAL0001691 – AIM19
p = 0.17
CAL0001709 – PUP2
p = 0.968
CAL0001726 – LSC2
p = 0.0454
CAL0001674
p = 0.0623
CAL0001692 – HHT2
p = 0.876
CAL0001714 – ADE5,7
p = 0.0459
CAL0001729 – RTC3
p = 1.76e–06
log10 normalised abundance
control
salt stressed

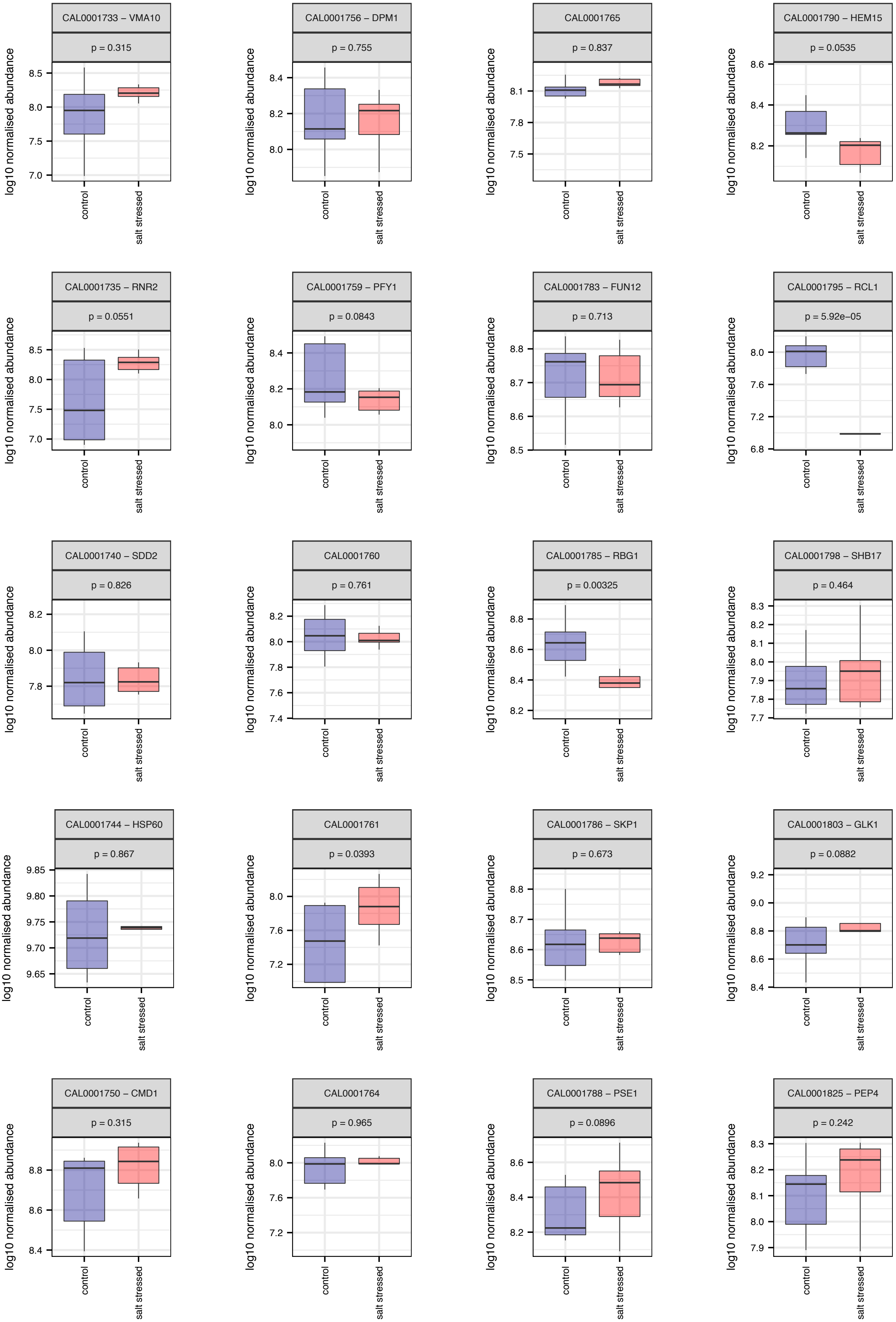
CAL0001733 – VMA10
p = 0.315
CAL0001756 – DPM1
p = 0.755
CAL0001765
p = 0.837
CAL0001790 – HEM15
p = 0.0535
CAL0001735 – RNR2
p = 0.0551
CAL0001759 – PFY1
p = 0.0843
CAL0001783 – FUN12
p = 0.713
CAL0001795 – RCL1
p = 5.92e−05
CAL0001740 – SDD2
p = 0.826
CAL0001760
p = 0.761
CAL0001785 – RBG1
p = 0.00325
CAL0001798 – SHB17
p = 0.464
CAL0001744 – HSP60
p = 0.867
CAL0001761
p = 0.0393
CAL0001786 – SKP1
p = 0.673
CAL0001803 – GLK1
p = 0.0882
CAL0001750 – CMD1
p = 0.315
CAL0001764
p = 0.965
CAL0001788 – PSE1
p = 0.0896
CAL0001825 – PEP4
p = 0.242
log10 normalised abundance
control
salt stressed

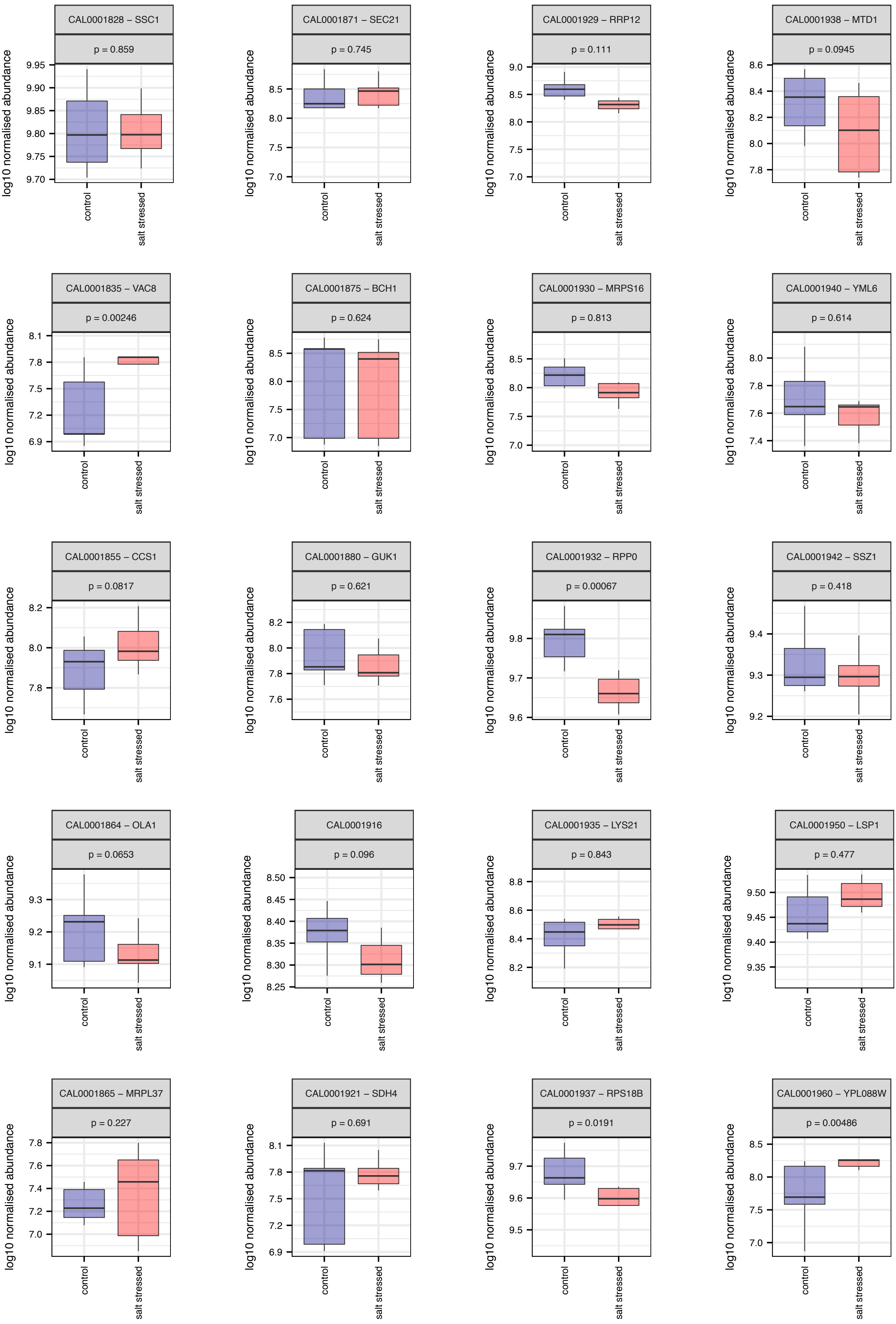
CAL0001828 – SSC1
p = 0.859
CAL0001871 – SEC21
p = 0.745
CAL0001929 – RRP12
p = 0.111
CAL0001938 – MTD1
p = 0.0945
CAL0001835 – VAC8
p = 0.00246
CAL0001875 – BCH1
p = 0.624
CAL0001930 – MRPS16
p = 0.813
CAL0001940 – YML6
p = 0.614
CAL0001855 – CCS1
p = 0.0817
CAL0001880 – GUK1
p = 0.621
CAL0001932 – RPP0
p = 0.00067
CAL0001942 – SSZ1
p = 0.418
CAL0001864 – OLA1
p = 0.0653
CAL0001916
p = 0.096
CAL0001935 – LYS21
p = 0.843
CAL0001950 – LSP1
p = 0.477
CAL0001865 – MRPL37
p = 0.227
CAL0001921 – SDH4
p = 0.691
CAL0001937 – RPS18B
p = 0.0191
CAL0001960 – YPL088W
p = 0.00486
log10 normalised abundance
control
salt stressed

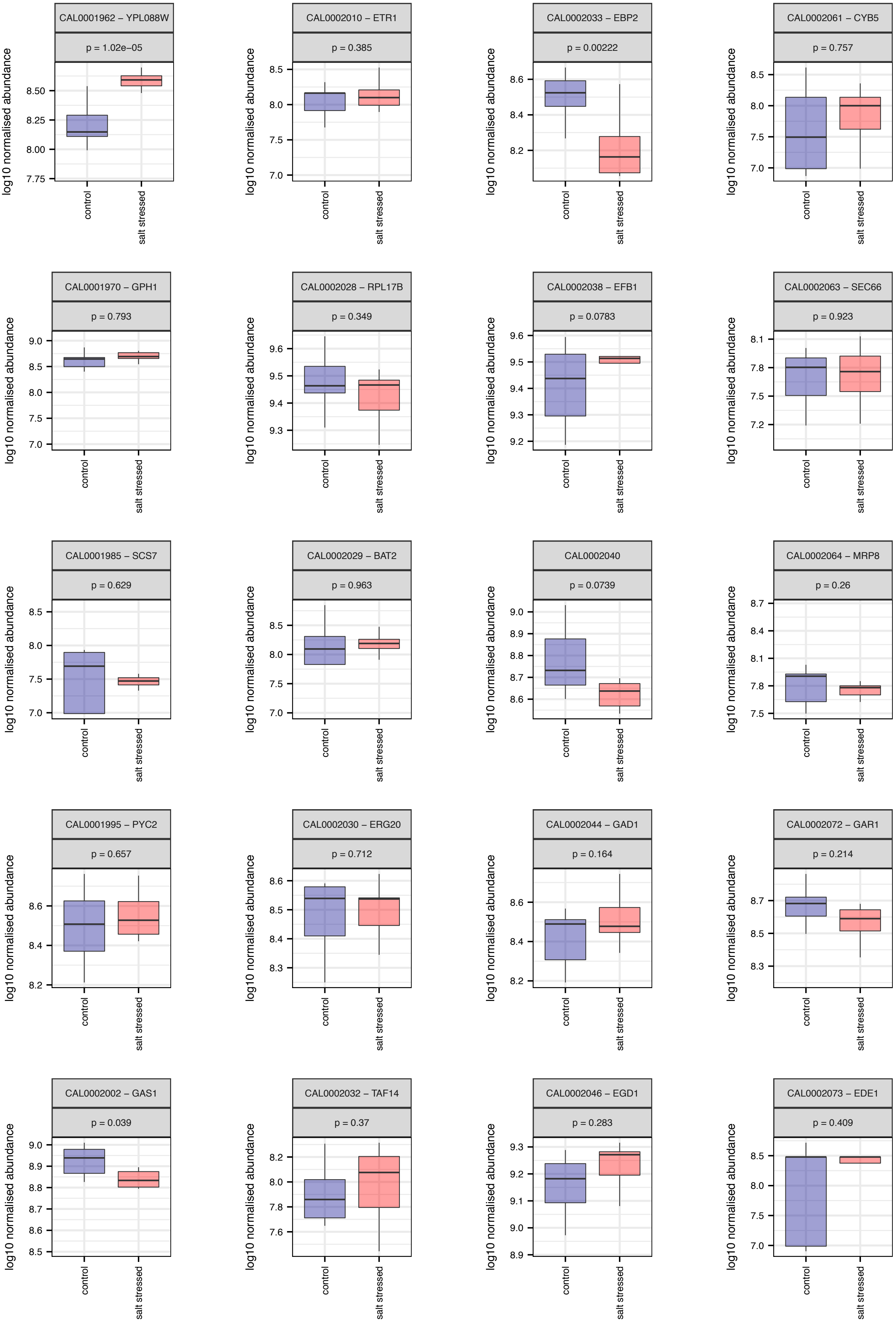
CAL0001962 – YPL088W
p = 1.02e−05
CAL0002010 – ETR1
p = 0.385
CAL0002033 – EBP2
p = 0.00222
CAL0002061 – CYB5
p = 0.757
CAL0001970 – GPH1
p = 0.793
CAL0002028 – RPL17B
p = 0.349
CAL0002038 – EFB1
p = 0.0783
CAL0002063 – SEC66
p = 0.923
CAL0001985 – SCS7
p = 0.629
CAL0002029 – BAT2
p = 0.963
CAL0002040
p = 0.0739
CAL0002064 – MRP8
p = 0.26
CAL0001995 – PYC2
p = 0.657
CAL0002030 – ERG20
p = 0.712
CAL0002044 – GAD1
p = 0.164
CAL0002072 – GAR1
p = 0.214
CAL0002002 – GAS1
p = 0.039
CAL0002032 – TAF14
p = 0.37
CAL0002046 – EGD1
p = 0.283
CAL0002073 – EDE1
p = 0.409
log10 normalised abundance
control
salt stressed

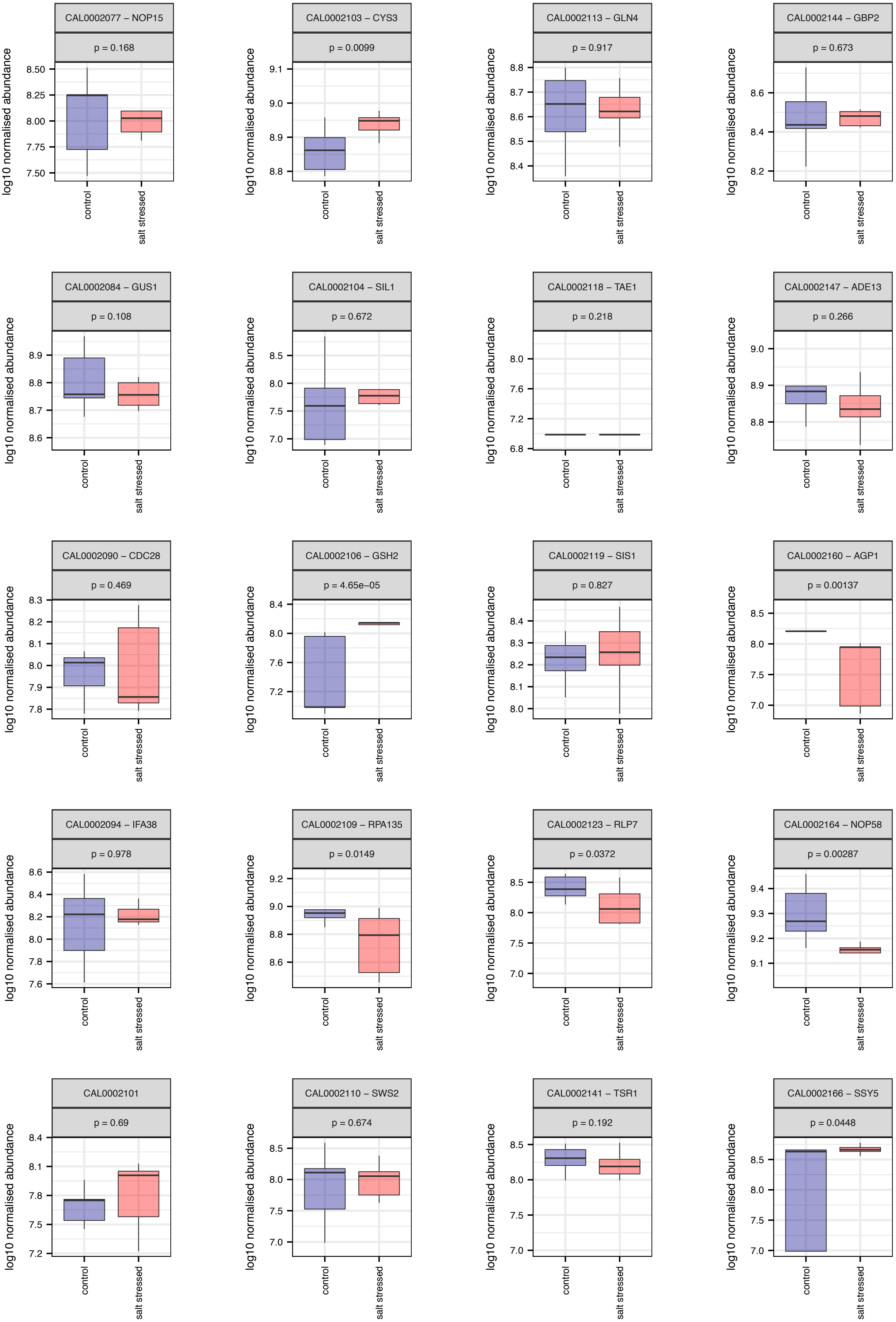
CAL0002077 – NOP15
p = 0.168
CAL0002103 – CYS3
p = 0.0099
CAL0002113 – GLN4
p = 0.917
CAL0002144 – GBP2
p = 0.673
CAL0002084 – GUS1
p = 0.108
CAL0002104 – SIL1
p = 0.672
CAL0002118 – TAE1
p = 0.218
CAL0002147 – ADE13
p = 0.266
CAL0002090 – CDC28
p = 0.469
CAL0002106 – GSH2
p = 4.65e–05
CAL0002119 – SIS1
p = 0.827
CAL0002160 – AGP1
p = 0.00137
CAL0002094 – IFA38
p = 0.978
CAL0002109 – RPA135
p = 0.0149
CAL0002123 – RLP7
p = 0.0372
CAL0002164 – NOP58
p = 0.00287
CAL0002101
p = 0.69
CAL0002110 – SWS2
p = 0.674
CAL0002141 – TSR1
p = 0.192
CAL0002166 – SSY5
p = 0.0448
log10 normalised abundance
control
salt stressed

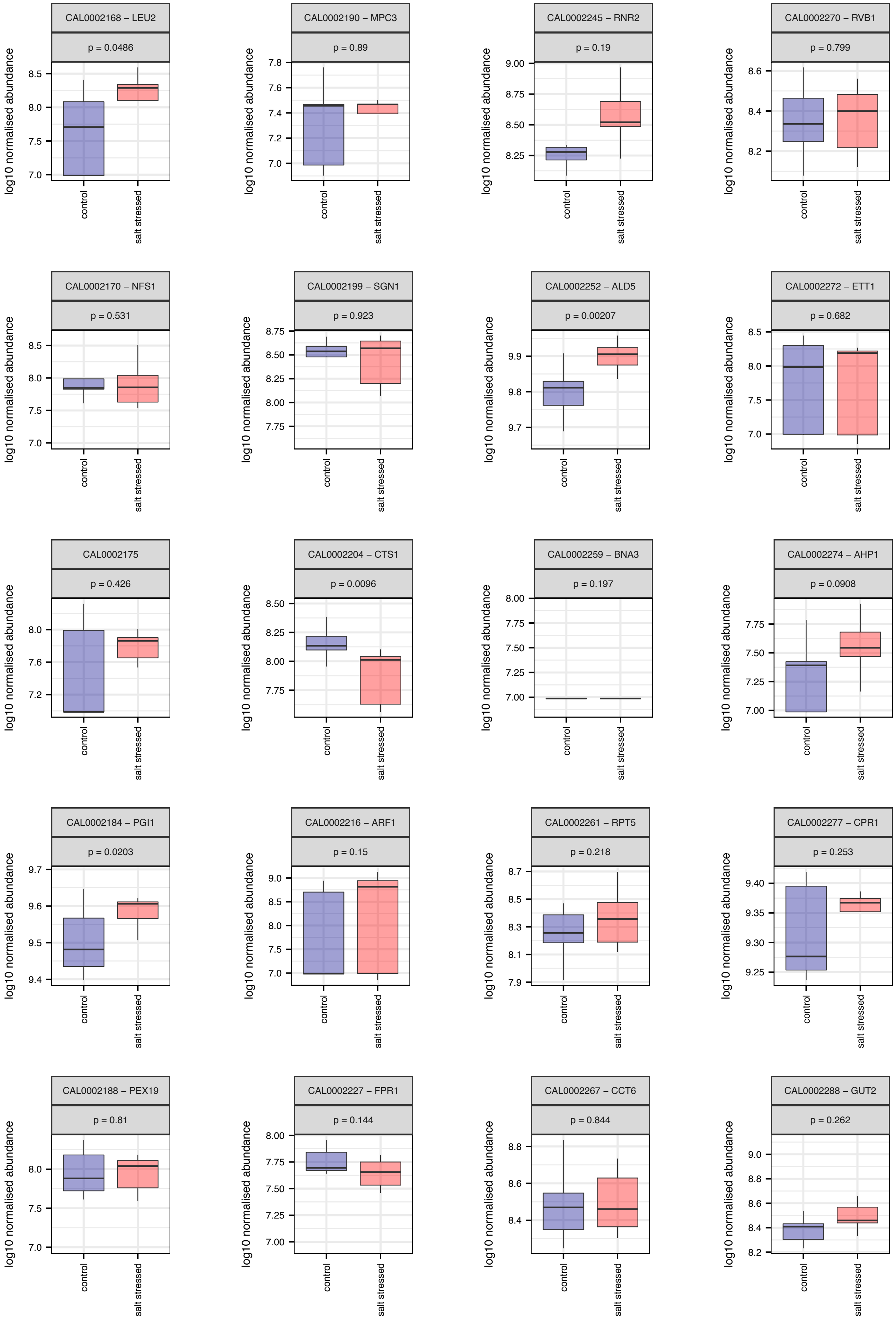
CAL0002168 – LEU2
p = 0.0486
8.5
8.0
7.5
7.0
log10 normalised abundance
control
salt stressed
CAL0002190 – MPC3
p = 0.89
7.8
7.6
7.4
7.2
7.0
log10 normalised abundance
control
salt stressed
CAL0002245 – RNR2
p = 0.19
9.00
8.75
8.50
8.25
log10 normalised abundance
control
salt stressed
CAL0002270 – RVB1
p = 0.799
8.6
8.4
8.2
log10 normalised abundance
control
salt stressed
CAL0002170 – NFS1
p = 0.531
8.5
8.0
7.5
7.0
log10 normalised abundance
control
salt stressed
CAL0002199 – SGN1
p = 0.923
8.75
8.50
8.25
8.00
7.75
log10 normalised abundance
control
salt stressed
CAL0002252 – ALD5
p = 0.00207
9.9
9.8
9.7
log10 normalised abundance
control
salt stressed
CAL0002272 – ETT1
p = 0.682
8.5
8.0
7.5
7.0
log10 normalised abundance
control
salt stressed
CAL0002175
p = 0.426
8.0
7.6
7.2
log10 normalised abundance
control
salt stressed
CAL0002204 – CTS1
p = 0.0096
8.50
8.25
8.00
7.75
log10 normalised abundance
control
salt stressed
CAL0002259 – BNA3
p = 0.197
8.00
7.75
7.50
7.25
7.00
log10 normalised abundance
control
salt stressed
CAL0002274 – AHP1
p = 0.0908
7.75
7.50
7.25
7.00
log10 normalised abundance
control
salt stressed
CAL0002184 – PGI1
p = 0.0203
9.7
9.6
9.5
9.4
log10 normalised abundance
control
salt stressed
CAL0002216 – ARF1
p = 0.15
9.0
8.5
8.0
7.5
7.0
log10 normalised abundance
control
salt stressed
CAL0002261 – RPT5
p = 0.218
8.7
8.5
8.3
8.1
7.9
log10 normalised abundance
control
salt stressed
CAL0002277 – CPR1
p = 0.253
9.40
9.35
9.30
9.25
log10 normalised abundance
control
salt stressed
CAL0002188 – PEX19
p = 0.81
8.0
7.5
7.0
log10 normalised abundance
control
salt stressed
CAL0002227 – FPR1
p = 0.144
8.00
7.75
7.50
7.25
7.00
log10 normalised abundance
control
salt stressed
CAL0002267 – CCT6
p = 0.844
8.8
8.6
8.4
log10 normalised abundance
control
salt stressed
CAL0002288 – GUT2
p = 0.262
9.0
8.8
8.6
8.4
8.2
log10 normalised abundance
control
salt stressed

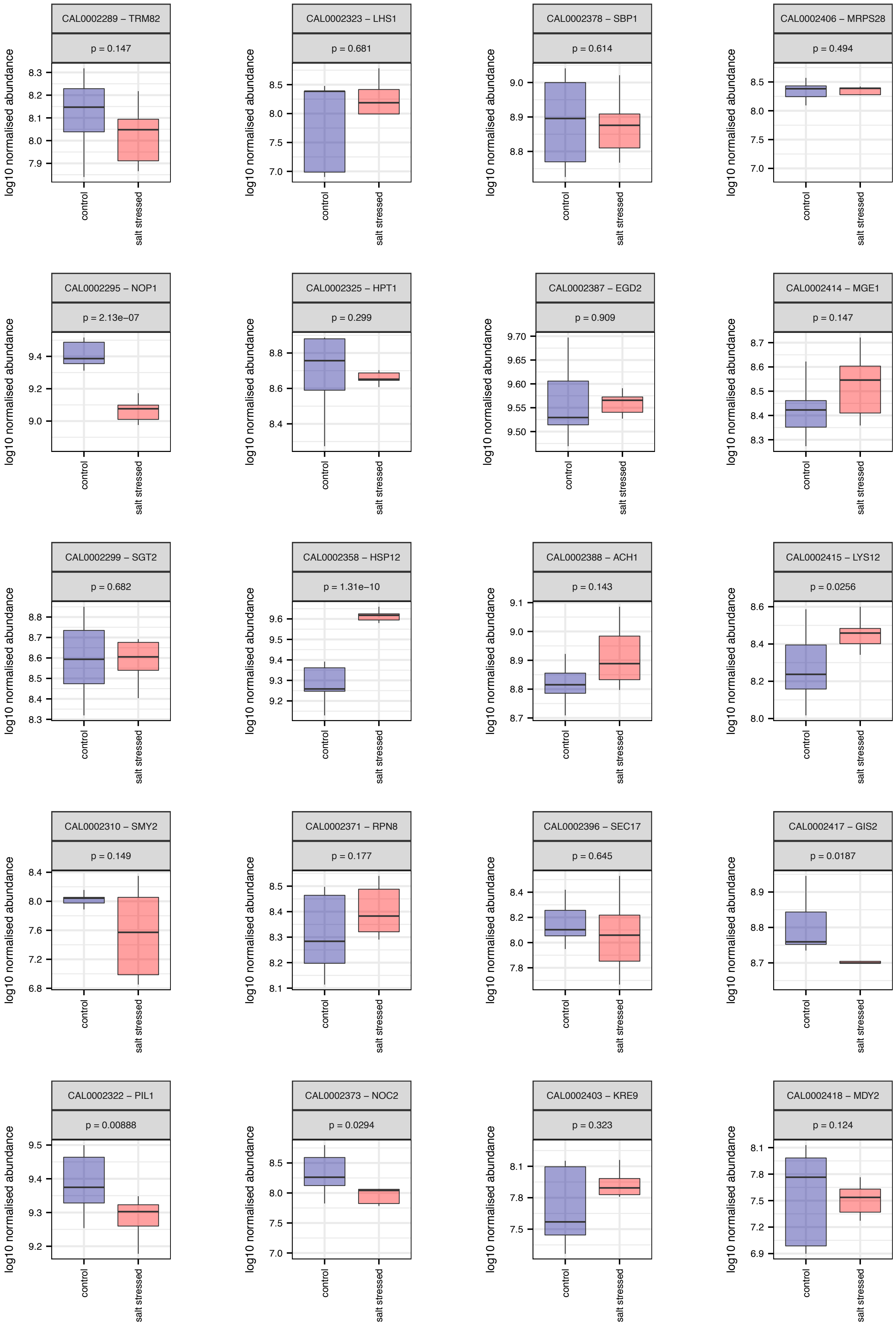
CAL0002289 – TRM82
p = 0.147
CAL0002323 – LHS1
p = 0.681
CAL0002378 – SBP1
p = 0.614
CAL0002406 – MRPS28
p = 0.494
CAL0002295 – NOP1
p = 2.13e–07
CAL0002325 – HPT1
p = 0.299
CAL0002387 – EGD2
p = 0.909
CAL0002414 – MGE1
p = 0.147
CAL0002299 – SGT2
p = 0.682
CAL0002358 – HSP12
p = 1.31e–10
CAL0002388 – ACH1
p = 0.143
CAL0002415 – LYS12
p = 0.0256
CAL0002310 – SMY2
p = 0.149
CAL0002371 – RPN8
p = 0.177
CAL0002396 – SEC17
p = 0.645
CAL0002417 – GIS2
p = 0.0187
CAL0002322 – PIL1
p = 0.00888
CAL0002373 – NOC2
p = 0.0294
CAL0002403 – KRE9
p = 0.323
CAL0002418 – MDY2
p = 0.124
log10 normalised abundance
control
salt stressed

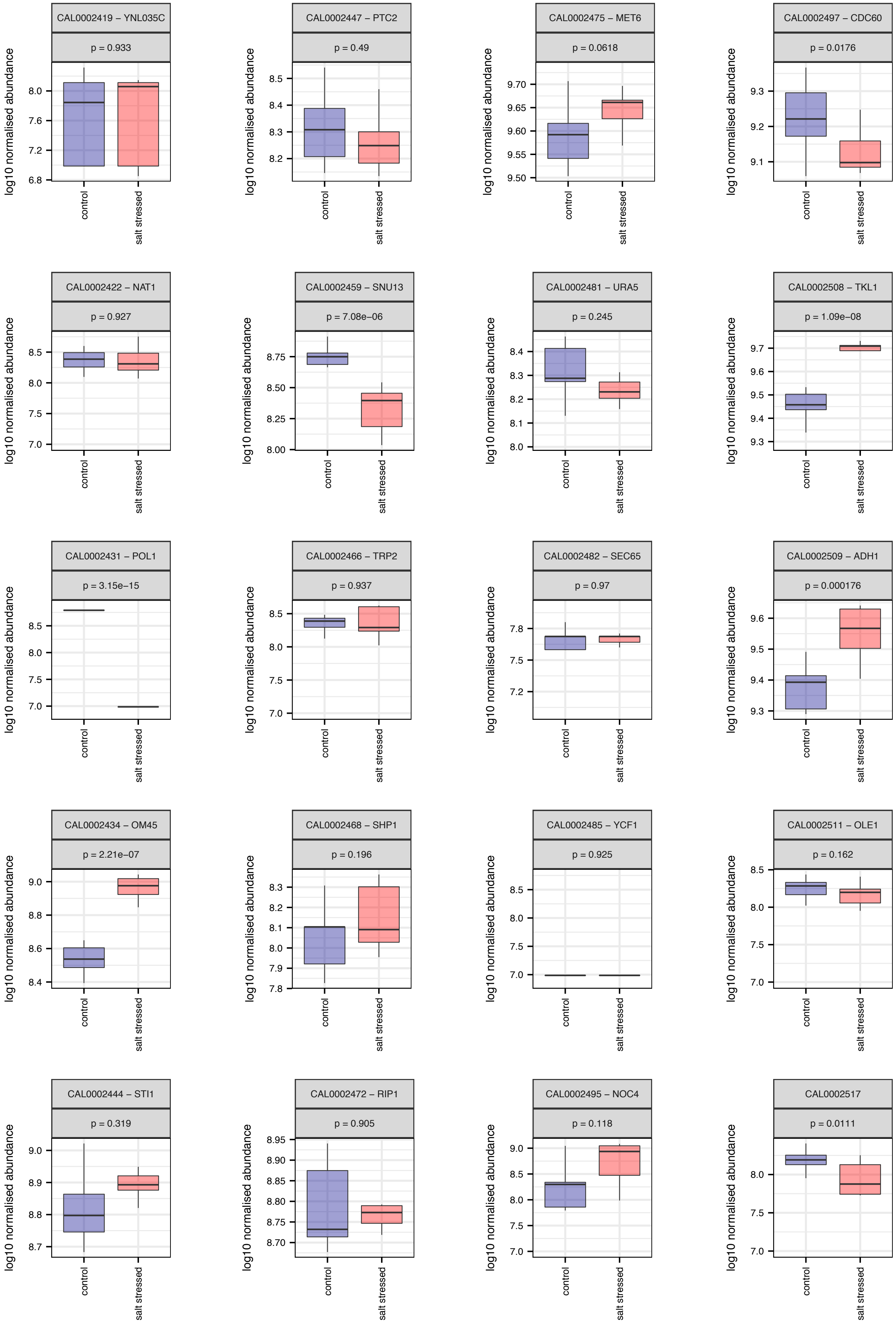
CAL0002419 – YNL035C
p = 0.933
CAL0002447 – PTC2
p = 0.49
CAL0002475 – MET6
p = 0.0618
CAL0002497 – CDC60
p = 0.0176
CAL0002422 – NAT1
p = 0.927
CAL0002459 – SNU13
p = 7.08e–06
CAL0002481 – URA5
p = 0.245
CAL0002508 – TKL1
p = 1.09e–08
CAL0002431 – POL1
p = 3.15e–15
CAL0002466 – TRP2
p = 0.937
CAL0002482 – SEC65
p = 0.97
CAL0002509 – ADH1
p = 0.000176
CAL0002434 – OM45
p = 2.21e–07
CAL0002468 – SHP1
p = 0.196
CAL0002485 – YCF1
p = 0.925
CAL0002511 – OLE1
p = 0.162
CAL0002444 – STI1
p = 0.319
CAL0002472 – RIP1
p = 0.905
CAL0002495 – NOC4
p = 0.118
CAL0002517
p = 0.0111
log10 normalised abundance
control
salt stressed

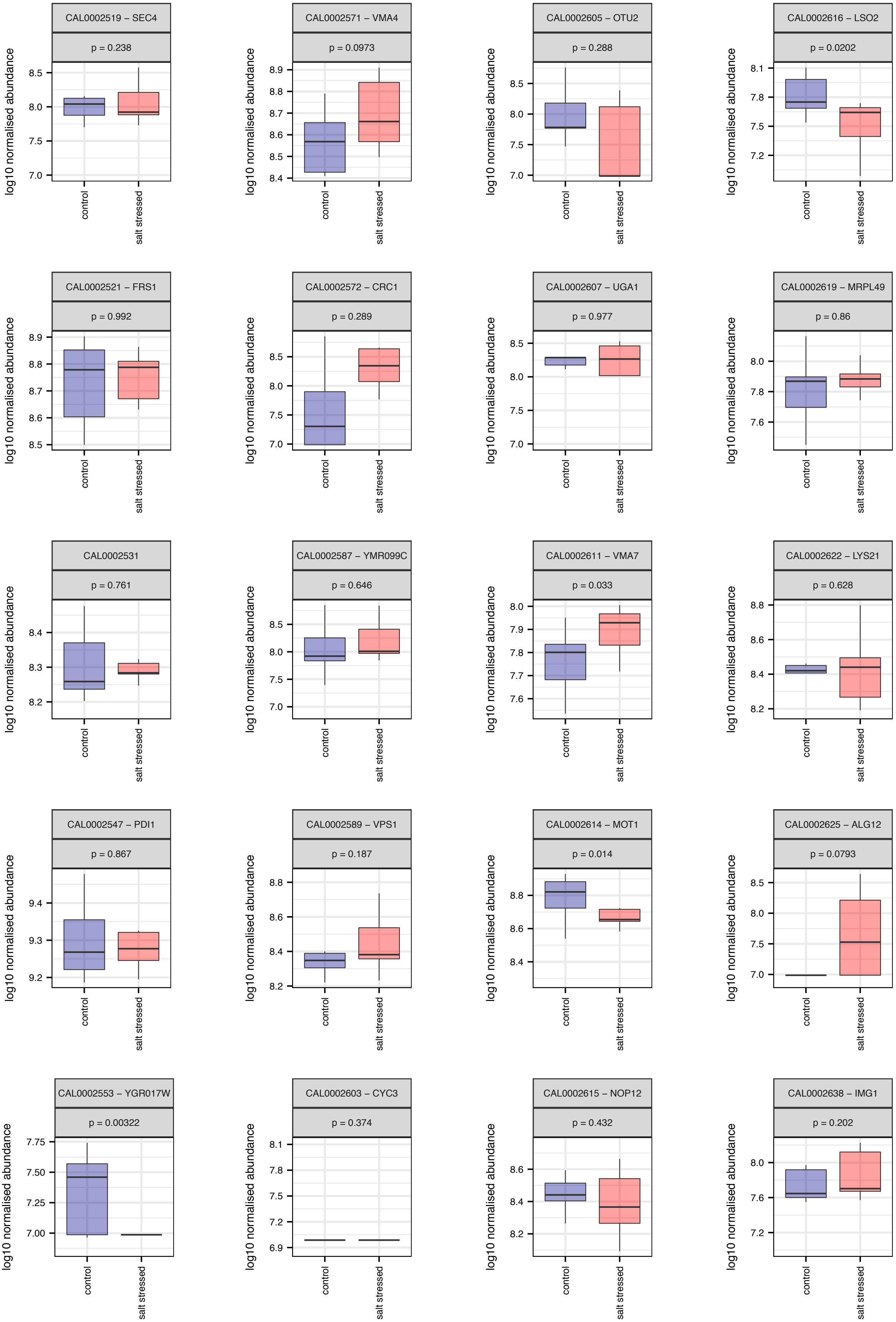
CAL0002519 – SEC4
p = 0.238
CAL0002571 – VMA4
p = 0.0973
CAL0002605 – OTU2
p = 0.288
CAL0002616 – LSO2
p = 0.0202
CAL0002521 – FRS1
p = 0.992
CAL0002572 – CRC1
p = 0.289
CAL0002607 – UGA1
p = 0.977
CAL0002619 – MRPL49
p = 0.86
CAL0002531
p = 0.761
CAL0002587 – YMR099C
p = 0.646
CAL0002611 – VMA7
p = 0.033
CAL0002622 – LYS21
p = 0.628
CAL0002547 – PDI1
p = 0.867
CAL0002589 – VPS1
p = 0.187
CAL0002614 – MOT1
p = 0.014
CAL0002625 – ALG12
p = 0.0793
CAL0002553 – YGR017W
p = 0.00322
CAL0002603 – CYC3
p = 0.374
CAL0002615 – NOP12
p = 0.432
CAL0002638 – IMG1
p = 0.202
log10 normalised abundance
control
salt stressed

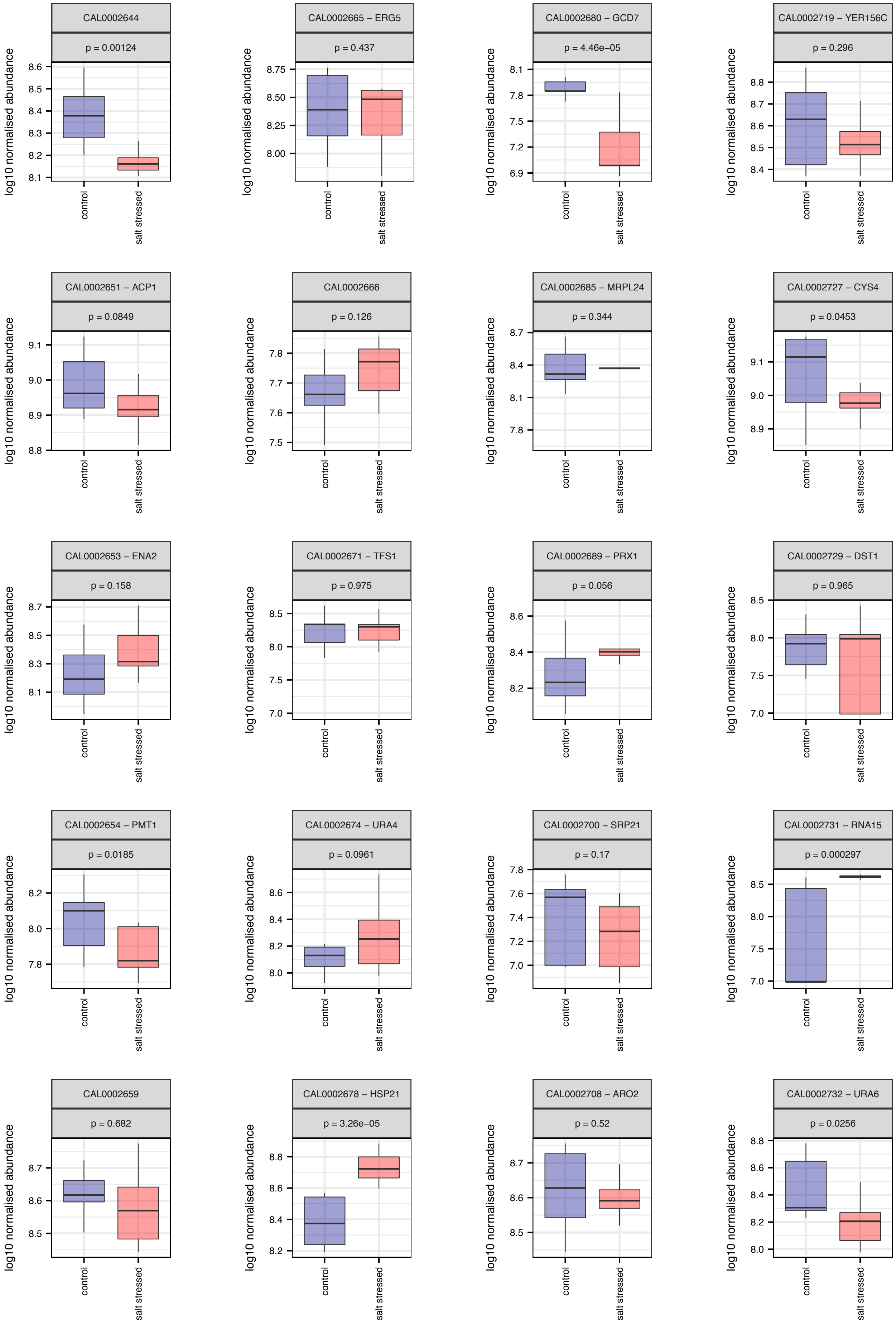
CAL0002644
p = 0.00124
CAL0002665 – ERG5
p = 0.437
CAL0002680 – GCD7
p = 4.46e–05
CAL0002719 – YER156C
p = 0.296
CAL0002651 – ACP1
p = 0.0849
CAL0002666
p = 0.126
CAL0002685 – MRPL24
p = 0.344
CAL0002727 – CYS4
p = 0.0453
CAL0002653 – ENA2
p = 0.158
CAL0002671 – TFS1
p = 0.975
CAL0002689 – PRX1
p = 0.056
CAL0002729 – DST1
p = 0.965
CAL0002654 – PMT1
p = 0.0185
CAL0002674 – URA4
p = 0.0961
CAL0002700 – SRP21
p = 0.17
CAL0002731 – RNA15
p = 0.000297
CAL0002659
p = 0.682
CAL0002678 – HSP21
p = 3.26e–05
CAL0002708 – ARO2
p = 0.52
CAL0002732 – URA6
p = 0.0256
log10 normalised abundance
control
salt stressed

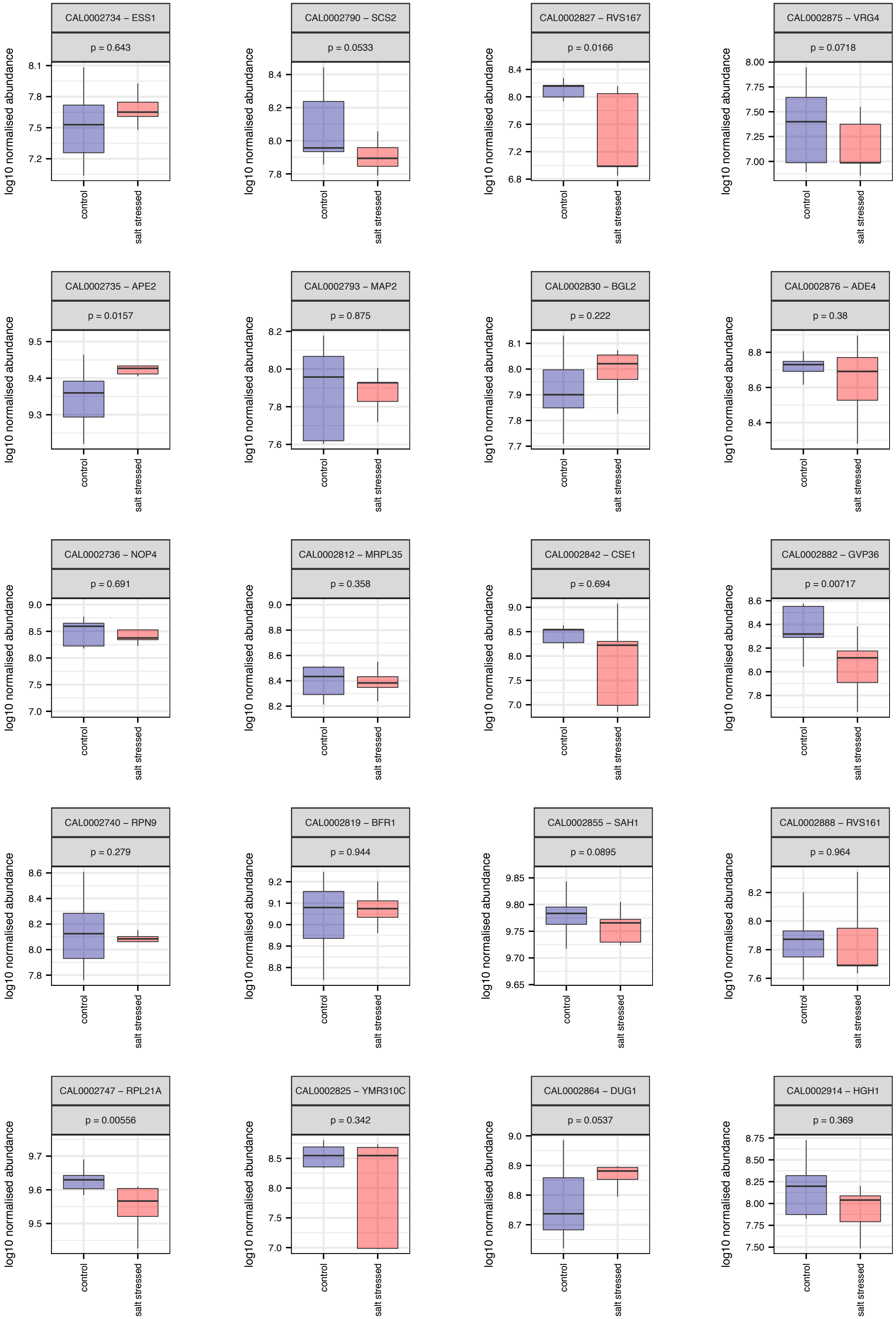
CAL0002734 – ESS1
p = 0.643
CAL0002790 – SCS2
p = 0.0533
CAL0002827 – RVS167
p = 0.0166
CAL0002875 – VRG4
p = 0.0718
CAL0002735 – APE2
p = 0.0157
CAL0002793 – MAP2
p = 0.875
CAL0002830 – BGL2
p = 0.222
CAL0002876 – ADE4
p = 0.38
CAL0002736 – NOP4
p = 0.691
CAL0002812 – MRPL35
p = 0.358
CAL0002842 – CSE1
p = 0.694
CAL0002882 – GVP36
p = 0.00717
CAL0002740 – RPN9
p = 0.279
CAL0002819 – BFR1
p = 0.944
CAL0002855 – SAH1
p = 0.0895
CAL0002888 – RVS161
p = 0.964
CAL0002747 – RPL21A
p = 0.00556
CAL0002825 – YMR310C
p = 0.342
CAL0002864 – DUG1
p = 0.0537
CAL0002914 – HGH1
p = 0.369
log10 normalised abundance
control
salt stressed

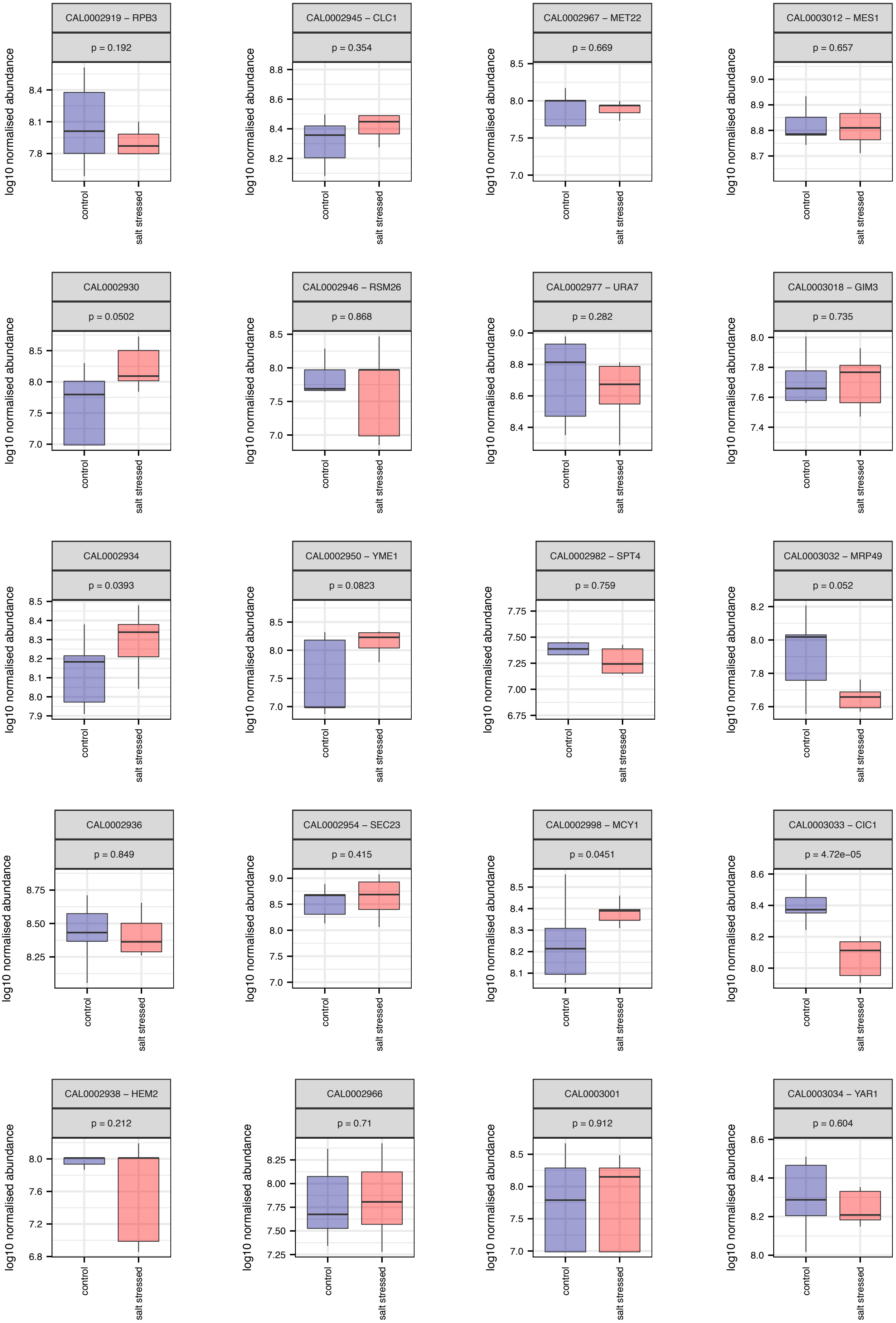
CAL0002919 – RPB3
p = 0.192
CAL0002945 – CLC1
p = 0.354
CAL0002967 – MET22
p = 0.669
CAL0003012 – MES1
p = 0.657
CAL0002930
p = 0.0502
CAL0002946 – RSM26
p = 0.868
CAL0002977 – URA7
p = 0.282
CAL0003018 – GIM3
p = 0.735
CAL0002934
p = 0.0393
CAL0002950 – YME1
p = 0.0823
CAL0002982 – SPT4
p = 0.759
CAL0003032 – MRP49
p = 0.052
CAL0002936
p = 0.849
CAL0002954 – SEC23
p = 0.415
CAL0002998 – MCY1
p = 0.0451
CAL0003033 – CIC1
p = 4.72e–05
CAL0002938 – HEM2
p = 0.212
CAL0002966
p = 0.71
CAL0003001
p = 0.912
CAL0003034 – YAR1
p = 0.604
log10 normalised abundance
control
salt stressed

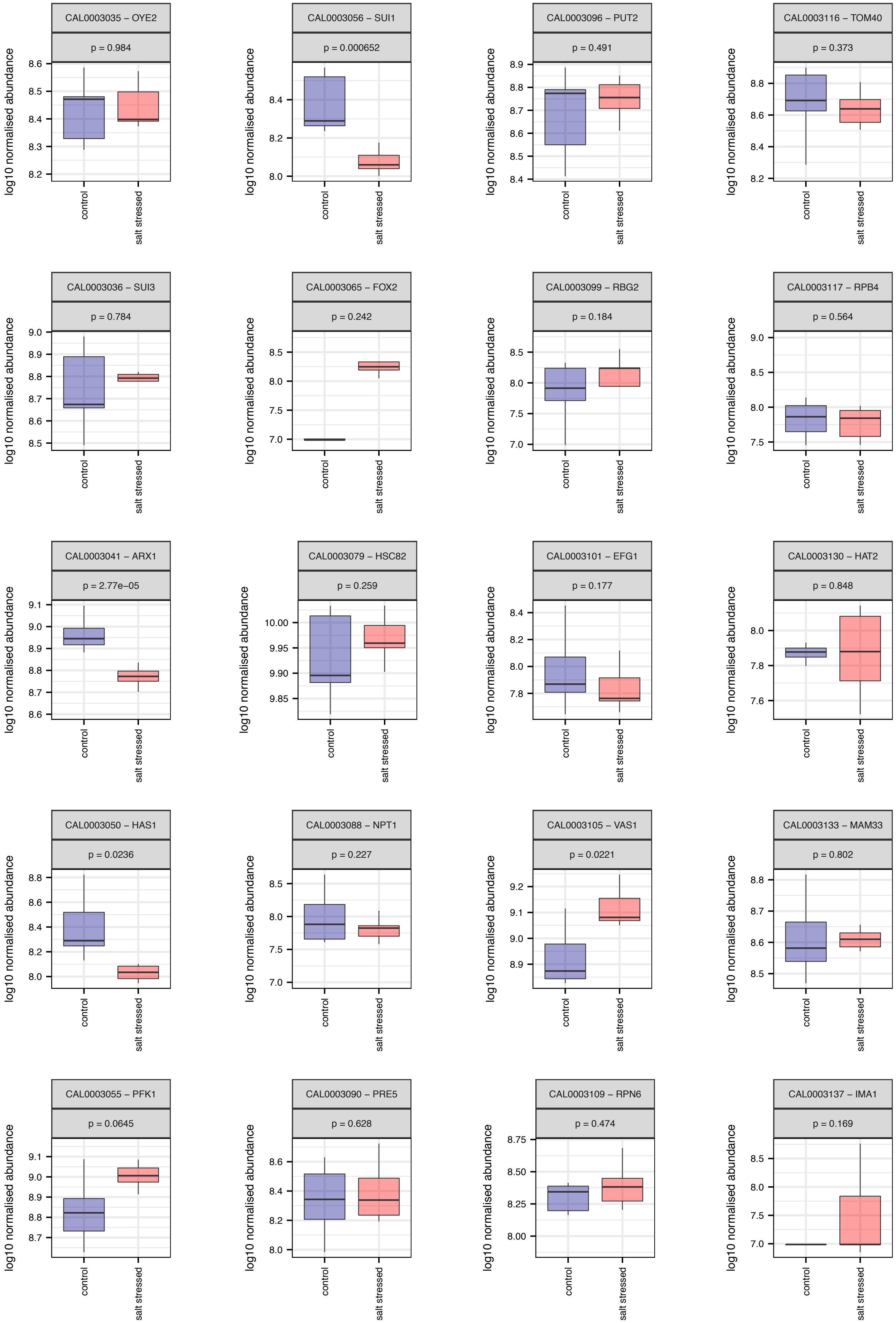
CAL0003035 – OYE2
p = 0.984
CAL0003056 – SUI1
p = 0.000652
CAL0003096 – PUT2
p = 0.491
CAL0003116 – TOM40
p = 0.373
CAL0003036 – SUI3
p = 0.784
CAL0003065 – FOX2
p = 0.242
CAL0003099 – RBG2
p = 0.184
CAL0003117 – RPB4
p = 0.564
CAL0003041 – ARX1
p = 2.77e−05
CAL0003079 – HSC82
p = 0.259
CAL0003101 – EFG1
p = 0.177
CAL0003130 – HAT2
p = 0.848
CAL0003050 – HAS1
p = 0.0236
CAL0003088 – NPT1
p = 0.227
CAL0003105 – VAS1
p = 0.0221
CAL0003133 – MAM33
p = 0.802
CAL0003055 – PFK1
p = 0.0645
CAL0003090 – PRE5
p = 0.628
CAL0003109 – RPN6
p = 0.474
CAL0003137 – IMA1
p = 0.169
log10 normalised abundance
control
salt stressed

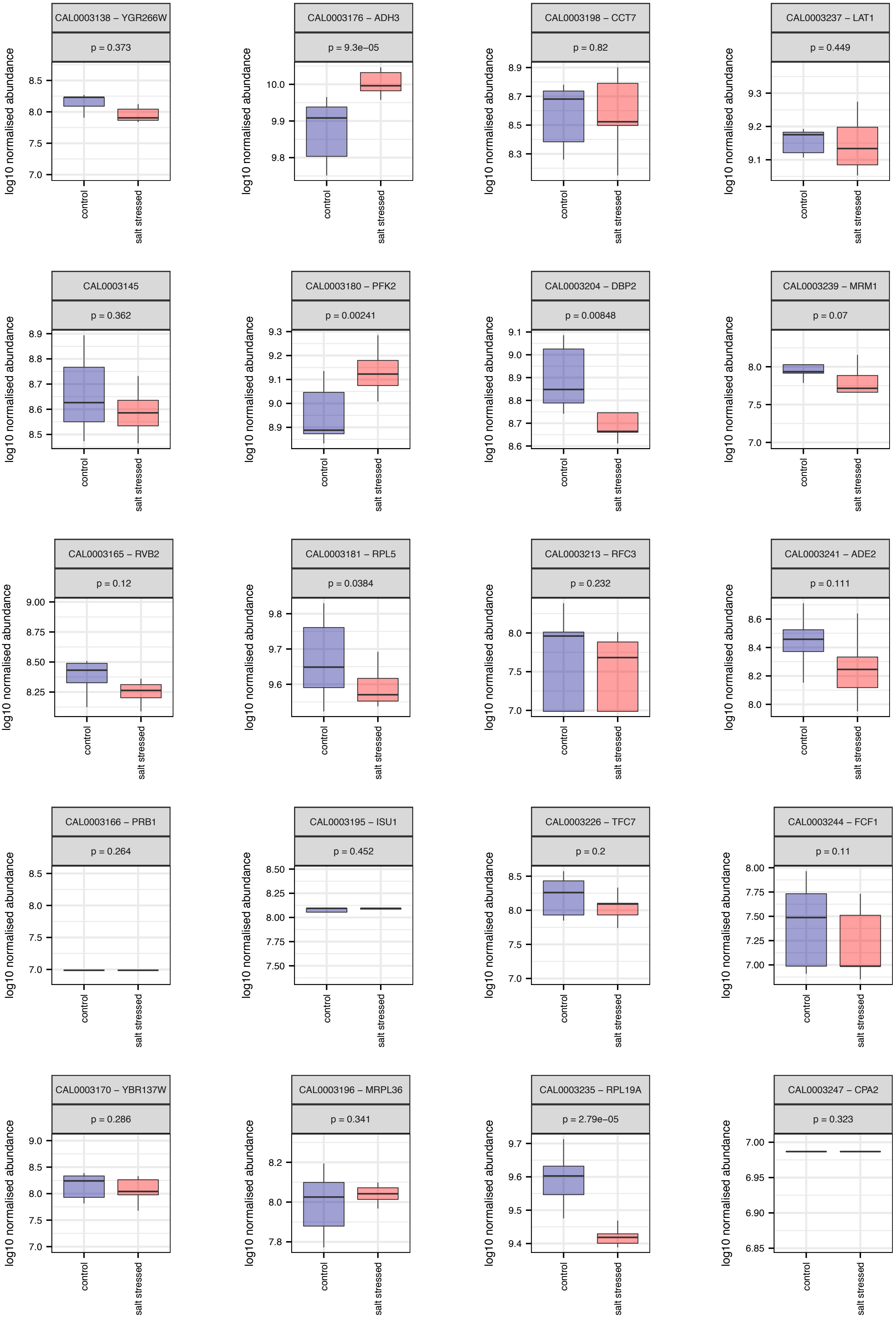
CAL0003138 – YGR266W
p = 0.373
CAL0003176 – ADH3
p = 9.3e−05
CAL0003198 – CCT7
p = 0.82
CAL0003237 – LAT1
p = 0.449
CAL0003145
p = 0.362
CAL0003180 – PFK2
p = 0.00241
CAL0003204 – DBP2
p = 0.00848
CAL0003239 – MRM1
p = 0.07
CAL0003165 – RVB2
p = 0.12
CAL0003181 – RPL5
p = 0.0384
CAL0003213 – RFC3
p = 0.232
CAL0003241 – ADE2
p = 0.111
CAL0003166 – PRB1
p = 0.264
CAL0003195 – ISU1
p = 0.452
CAL0003226 – TFC7
p = 0.2
CAL0003244 – FCF1
p = 0.11
CAL0003170 – YBR137W
p = 0.286
CAL0003196 – MRPL36
p = 0.341
CAL0003235 – RPL19A
p = 2.79e−05
CAL0003247 – CPA2
p = 0.323
log10 normalised abundance
control
salt stressed

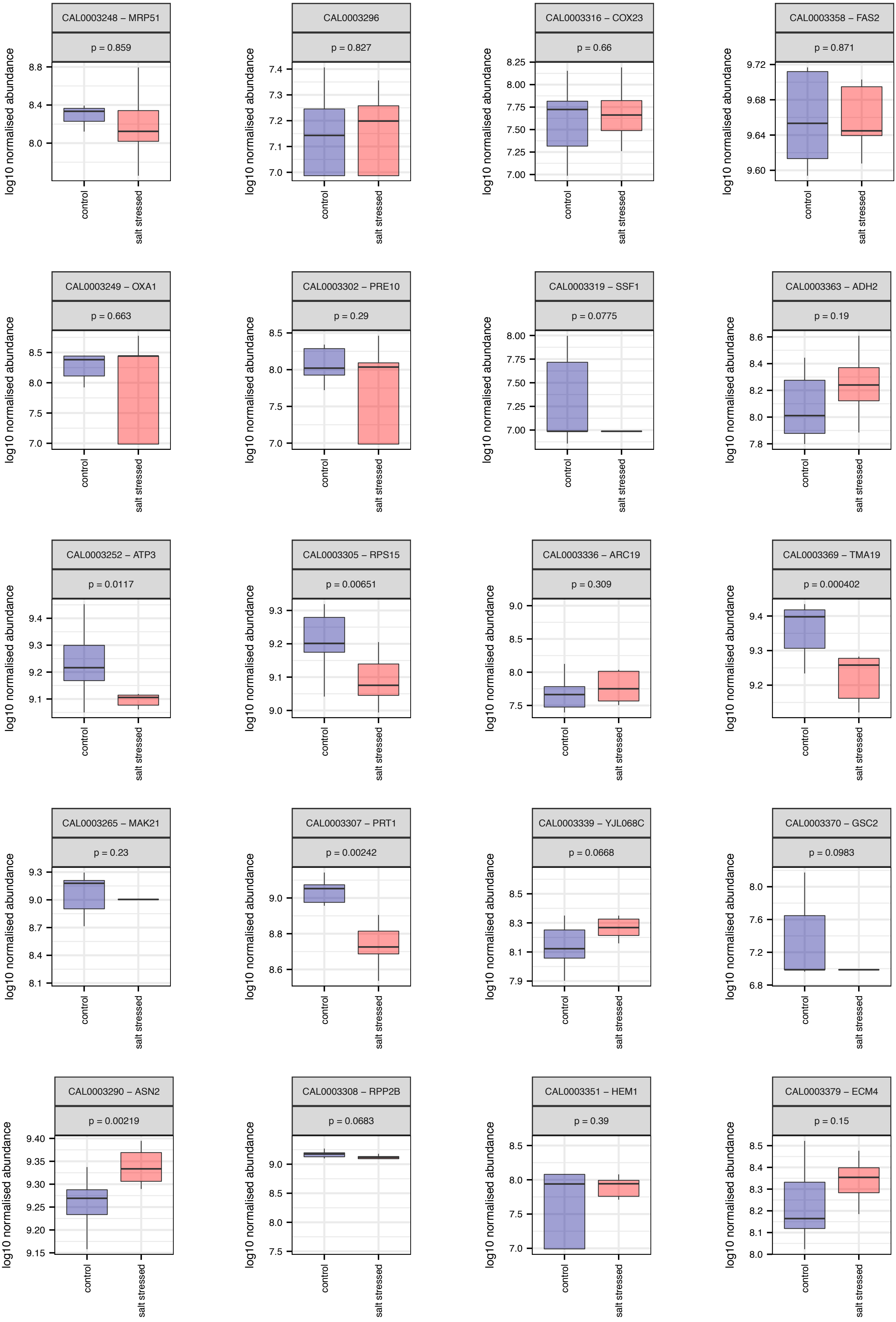
CAL0003248 – MRP51
p = 0.859
CAL0003296
p = 0.827
CAL0003316 – COX23
p = 0.66
CAL0003358 – FAS2
p = 0.871
CAL0003249 – OXA1
p = 0.663
CAL0003302 – PRE10
p = 0.29
CAL0003319 – SSF1
p = 0.0775
CAL0003363 – ADH2
p = 0.19
CAL0003252 – ATP3
p = 0.0117
CAL0003305 – RPS15
p = 0.00651
CAL0003336 – ARC19
p = 0.309
CAL0003369 – TMA19
p = 0.000402
CAL0003265 – MAK21
p = 0.23
CAL0003307 – PRT1
p = 0.00242
CAL0003339 – YJL068C
p = 0.0668
CAL0003370 – GSC2
p = 0.0983
CAL0003290 – ASN2
p = 0.00219
CAL0003308 – RPP2B
p = 0.0683
CAL0003351 – HEM1
p = 0.39
CAL0003379 – ECM4
p = 0.15
log10 normalised abundance
control
salt stressed

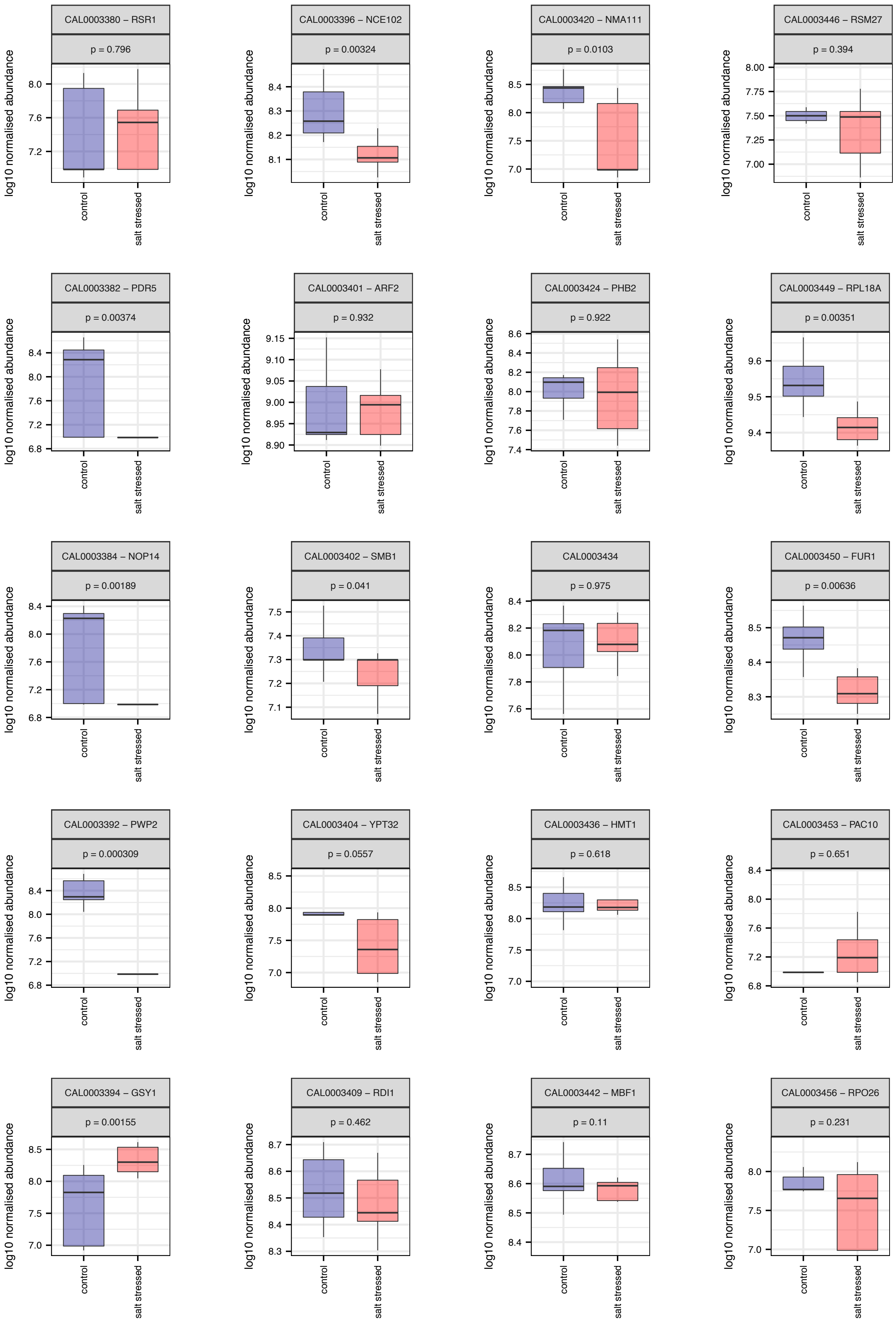
CAL0003380 – RSR1
p = 0.796
CAL0003396 – NCE102
p = 0.00324
CAL0003420 – NMA111
p = 0.0103
CAL0003446 – RSM27
p = 0.394
CAL0003382 – PDR5
p = 0.00374
CAL0003401 – ARF2
p = 0.932
CAL0003424 – PHB2
p = 0.922
CAL0003449 – RPL18A
p = 0.00351
CAL0003384 – NOP14
p = 0.00189
CAL0003402 – SMB1
p = 0.041
CAL0003434
p = 0.975
CAL0003450 – FUR1
p = 0.00636
CAL0003392 – PWP2
p = 0.000309
CAL0003404 – YPT32
p = 0.0557
CAL0003436 – HMT1
p = 0.618
CAL0003453 – PAC10
p = 0.651
CAL0003394 – GSY1
p = 0.00155
CAL0003409 – RDI1
p = 0.462
CAL0003442 – MBF1
p = 0.11
CAL0003456 – RPO26
p = 0.231
log10 normalised abundance
control
salt stressed

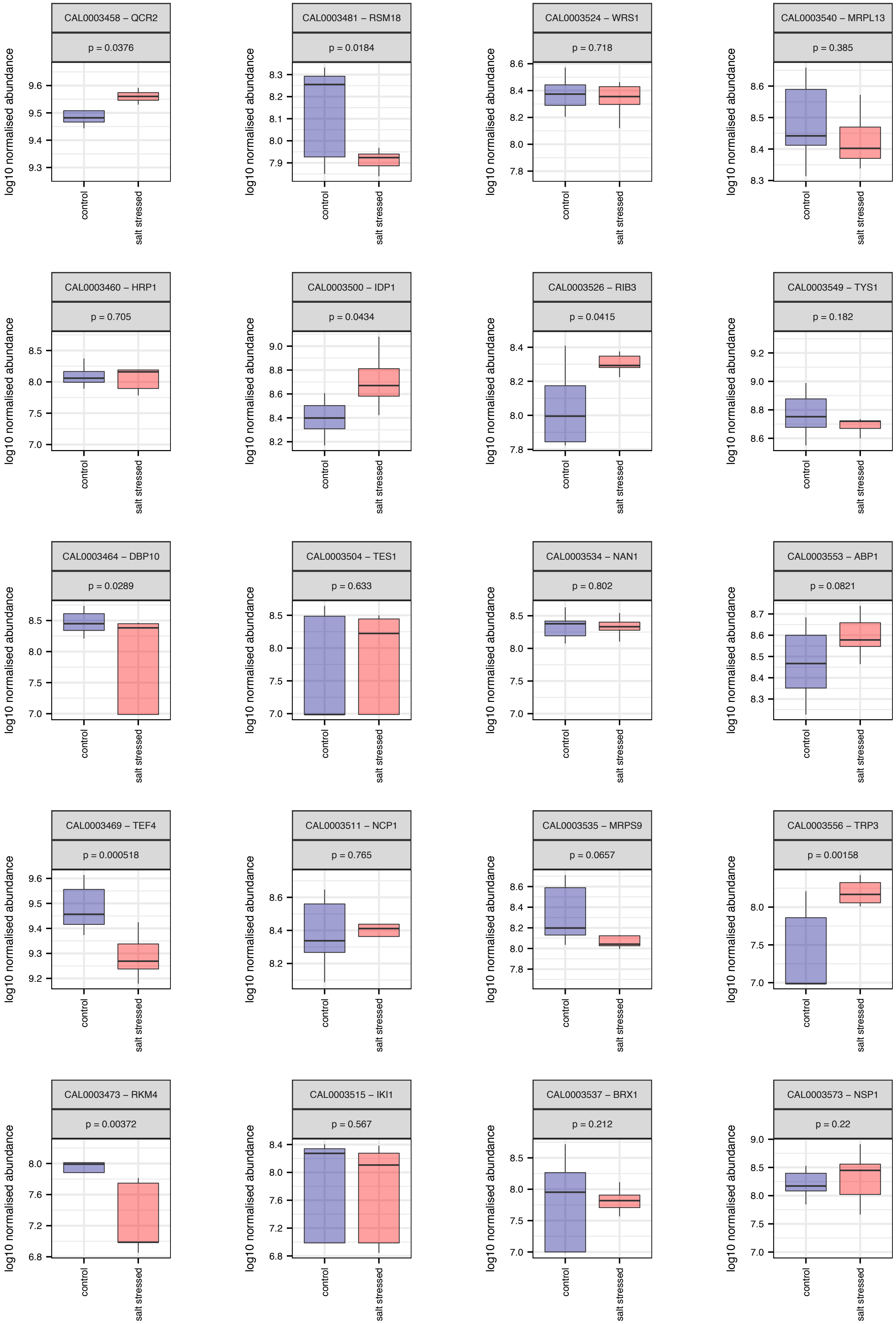
CAL0003458 – QCR2
p = 0.0376
CAL0003481 – RSM18
p = 0.0184
CAL0003524 – WRS1
p = 0.718
CAL0003540 – MRPL13
p = 0.385
CAL0003460 – HRP1
p = 0.705
CAL0003500 – IDP1
p = 0.0434
CAL0003526 – RIB3
p = 0.0415
CAL0003549 – TYS1
p = 0.182
CAL0003464 – DBP10
p = 0.0289
CAL0003504 – TES1
p = 0.633
CAL0003534 – NAN1
p = 0.802
CAL0003553 – ABP1
p = 0.0821
CAL0003469 – TEF4
p = 0.000518
CAL0003511 – NCP1
p = 0.765
CAL0003535 – MRPS9
p = 0.0657
CAL0003556 – TRP3
p = 0.00158
CAL0003473 – RKM4
p = 0.00372
CAL0003515 – IKI1
p = 0.567
CAL0003537 – BRX1
p = 0.212
CAL0003573 – NSP1
p = 0.22
log10 normalised abundance
control
salt stressed

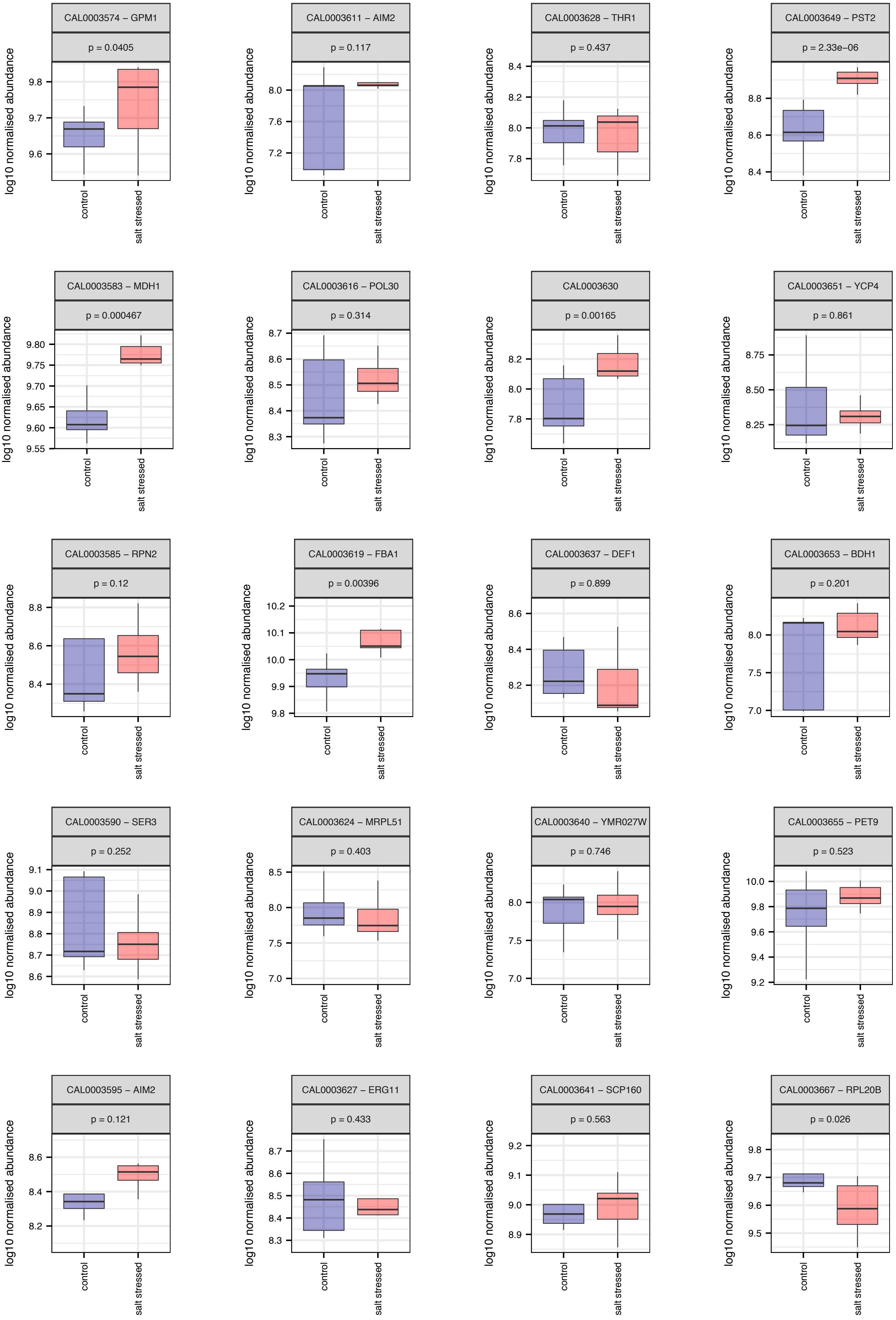
CAL0003574 – GPM1
p = 0.0405
CAL0003611 – AIM2
p = 0.117
CAL0003628 – THR1
p = 0.437
CAL0003649 – PST2
p = 2.33e–06
CAL0003583 – MDH1
p = 0.000467
CAL0003616 – POL30
p = 0.314
CAL0003630
p = 0.00165
CAL0003651 – YCP4
p = 0.861
CAL0003585 – RPN2
p = 0.12
CAL0003619 – FBA1
p = 0.00396
CAL0003637 – DEF1
p = 0.899
CAL0003653 – BDH1
p = 0.201
CAL0003590 – SER3
p = 0.252
CAL0003624 – MRPL51
p = 0.403
CAL0003640 – YMR027W
p = 0.746
CAL0003655 – PET9
p = 0.523
CAL0003595 – AIM2
p = 0.121
CAL0003627 – ERG11
p = 0.433
CAL0003641 – SCP160
p = 0.563
CAL0003667 – RPL20B
p = 0.026
log10 normalised abundance
control
salt stressed

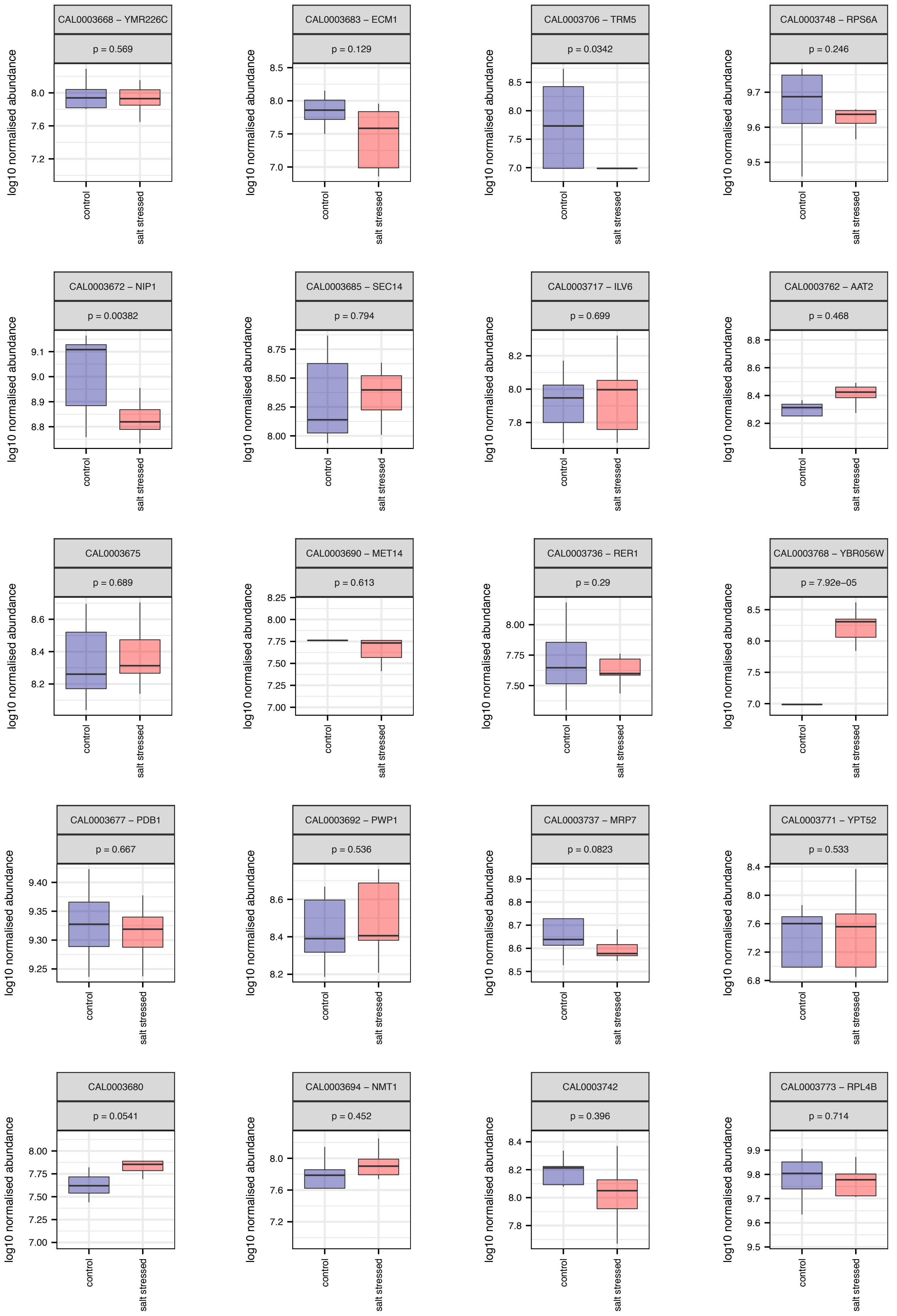
CAL0003668 – YMR226C
p = 0.569
8.0
7.6
7.2
CAL0003683 – ECM1
p = 0.129
8.5
8.0
7.5
7.0
CAL0003706 – TRM5
p = 0.0342
8.5
8.0
7.5
7.0
CAL0003748 – RPS6A
p = 0.246
9.7
9.6
9.5
CAL0003672 – NIP1
p = 0.00382
9.1
9.0
8.9
8.8
CAL0003685 – SEC14
p = 0.794
8.75
8.50
8.25
8.00
CAL0003717 – ILV6
p = 0.699
8.2
8.0
7.8
CAL0003762 – AAT2
p = 0.468
8.8
8.6
8.4
8.2
CAL0003675
p = 0.689
8.6
8.4
8.2
CAL0003690 – MET14
p = 0.613
8.25
8.00
7.75
7.50
7.25
7.00
CAL0003736 – RER1
p = 0.29
8.00
7.75
7.50
CAL0003768 – YBR056W
p = 7.92e−05
8.5
8.0
7.5
7.0
CAL0003677 – PDB1
p = 0.667
9.40
9.35
9.30
9.25
CAL0003692 – PWP1
p = 0.536
8.6
8.4
8.2
CAL0003737 – MRP7
p = 0.0823
8.9
8.8
8.7
8.6
8.5
CAL0003771 – YPT52
p = 0.533
8.4
8.0
7.6
7.2
6.8
CAL0003680
p = 0.0541
8.00
7.75
7.50
7.25
7.00
CAL0003694 – NMT1
p = 0.452
8.0
7.6
7.2
CAL0003742
p = 0.396
8.4
8.2
8.0
7.8
CAL0003773 – RPL4B
p = 0.714
9.9
9.8
9.7
9.6
9.5
log10 normalised abundance
control
salt stressed

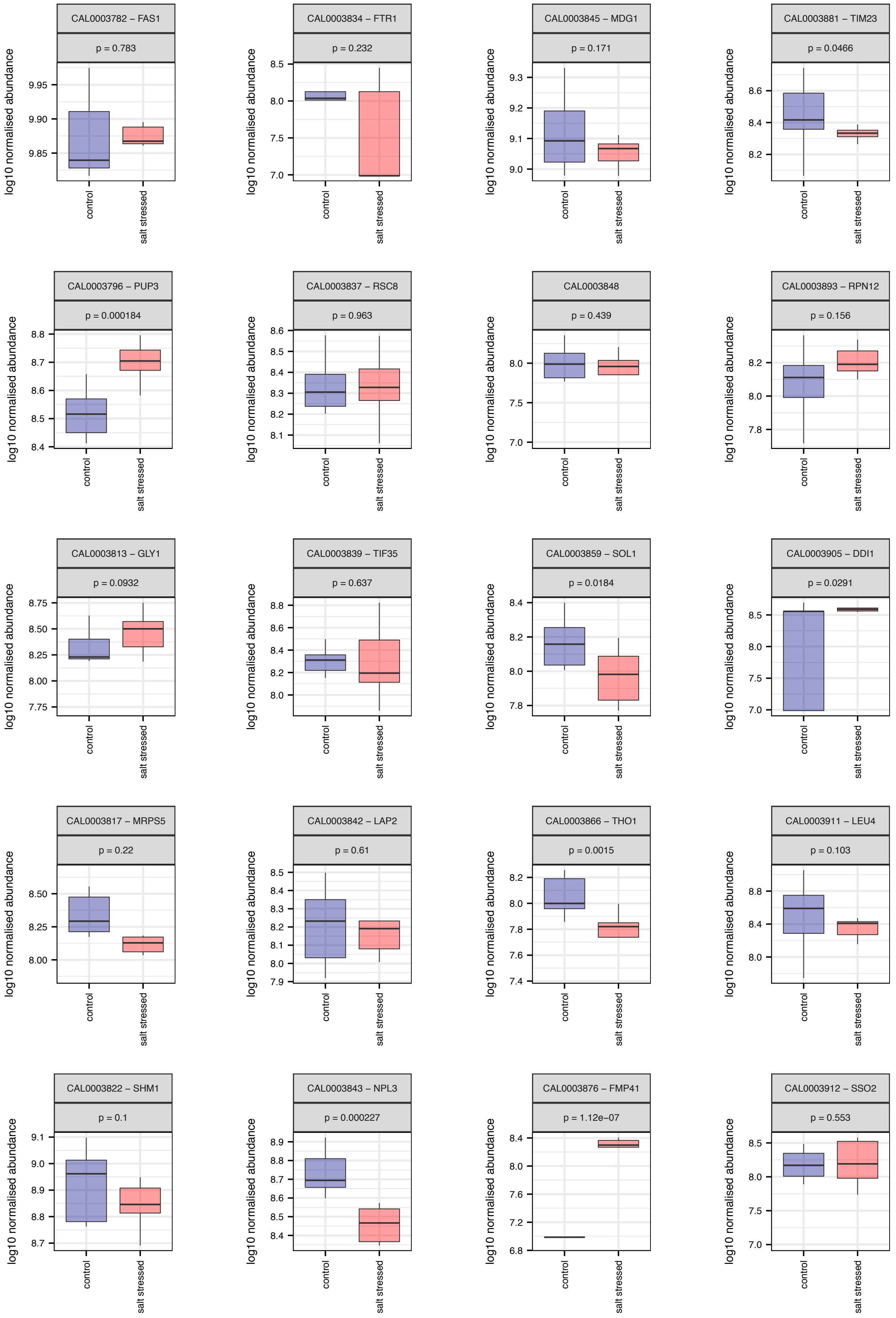
CAL0003782 – FAS1
p = 0.783
CAL0003834 – FTR1
p = 0.232
CAL0003845 – MDG1
p = 0.171
CAL0003881 – TIM23
p = 0.0466
CAL0003796 – PUP3
p = 0.000184
CAL0003837 – RSC8
p = 0.963
CAL0003848
p = 0.439
CAL0003893 – RPN12
p = 0.156
CAL0003813 – GLY1
p = 0.0932
CAL0003839 – TIF35
p = 0.637
CAL0003859 – SOL1
p = 0.0184
CAL0003905 – DDI1
p = 0.0291
CAL0003817 – MRPS5
p = 0.22
CAL0003842 – LAP2
p = 0.61
CAL0003866 – THO1
p = 0.0015
CAL0003911 – LEU4
p = 0.103
CAL0003822 – SHM1
p = 0.1
CAL0003843 – NPL3
p = 0.000227
CAL0003876 – FMP41
p = 1.12e–07
CAL0003912 – SSO2
p = 0.553
log10 normalised abundance
control
salt stressed

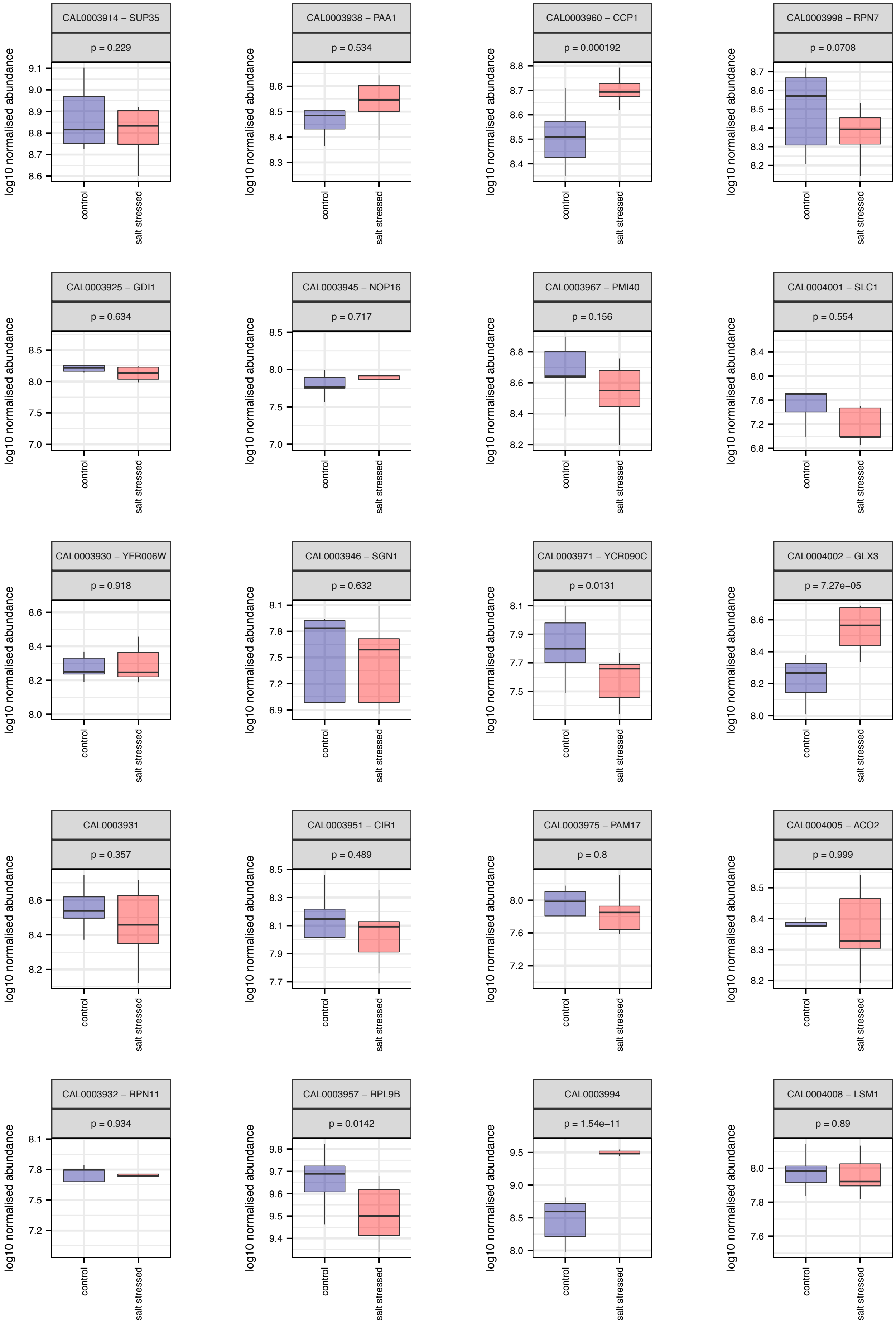
CAL0003914 – SUP35
p = 0.229
CAL0003938 – PAA1
p = 0.534
CAL0003960 – CCP1
p = 0.000192
CAL0003998 – RPN7
p = 0.0708
CAL0003925 – GDI1
p = 0.634
CAL0003945 – NOP16
p = 0.717
CAL0003967 – PMI40
p = 0.156
CAL0004001 – SLC1
p = 0.554
CAL0003930 – YFR006W
p = 0.918
CAL0003946 – SGN1
p = 0.632
CAL0003971 – YCR090C
p = 0.0131
CAL0004002 – GLX3
p = 7.27e−05
CAL0003931
p = 0.357
CAL0003951 – CIR1
p = 0.489
CAL0003975 – PAM17
p = 0.8
CAL0004005 – ACO2
p = 0.999
CAL0003932 – RPN11
p = 0.934
CAL0003957 – RPL9B
p = 0.0142
CAL0003994
p = 1.54e−11
CAL0004008 – LSM1
p = 0.89
log10 normalised abundance
control
salt stressed

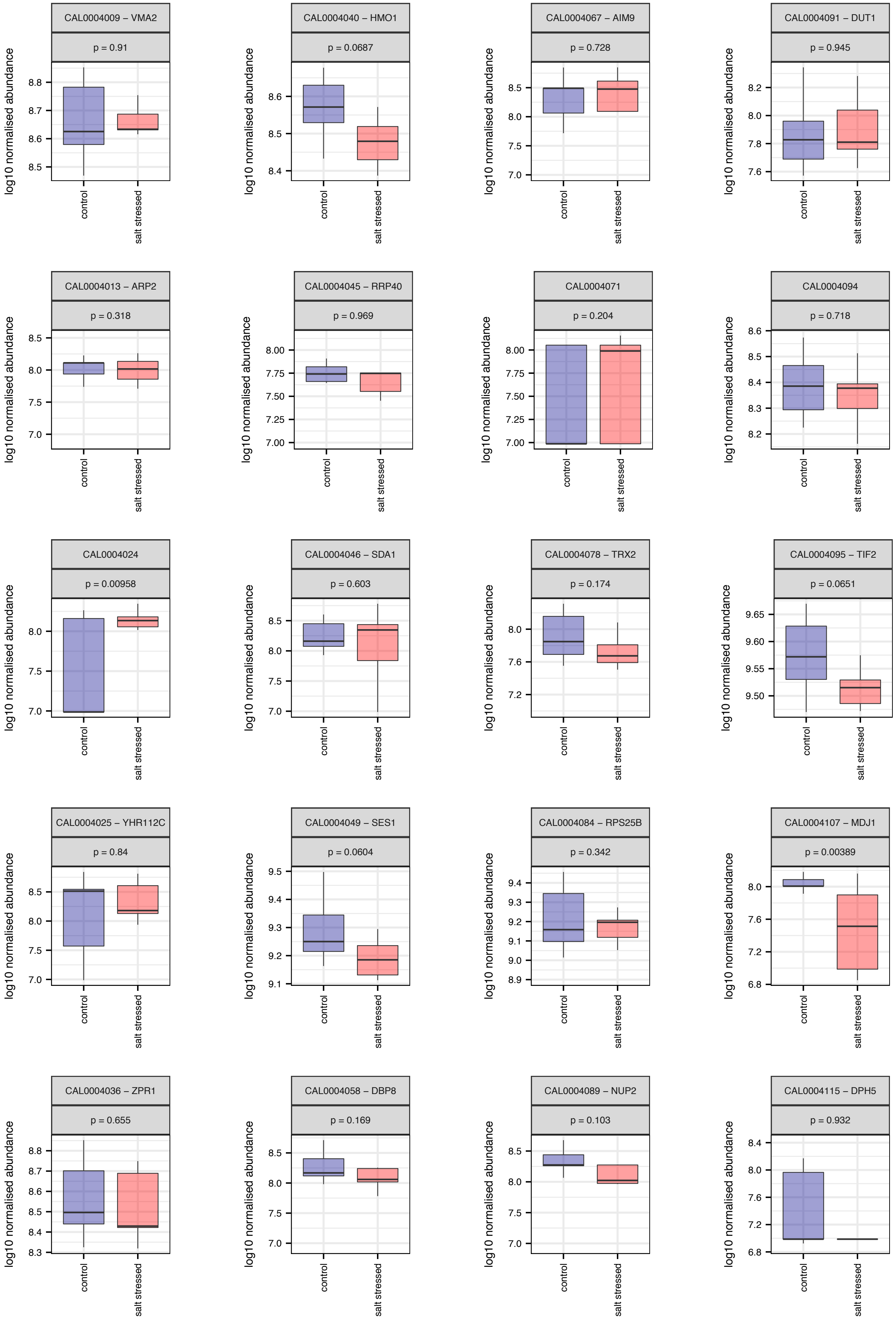
CAL0004009 – VMA2
p = 0.91
CAL0004040 – HMO1
p = 0.0687
CAL0004067 – AIM9
p = 0.728
CAL0004091 – DUT1
p = 0.945
CAL0004013 – ARP2
p = 0.318
CAL0004045 – RRP40
p = 0.969
CAL0004071
p = 0.204
CAL0004094
p = 0.718
CAL0004024
p = 0.00958
CAL0004046 – SDA1
p = 0.603
CAL0004078 – TRX2
p = 0.174
CAL0004095 – TIF2
p = 0.0651
CAL0004025 – YHR112C
p = 0.84
CAL0004049 – SES1
p = 0.0604
CAL0004084 – RPS25B
p = 0.342
CAL0004107 – MDJ1
p = 0.00389
CAL0004036 – ZPR1
p = 0.655
CAL0004058 – DBP8
p = 0.169
CAL0004089 – NUP2
p = 0.103
CAL0004115 – DPH5
p = 0.932
log10 normalised abundance
control
salt stressed

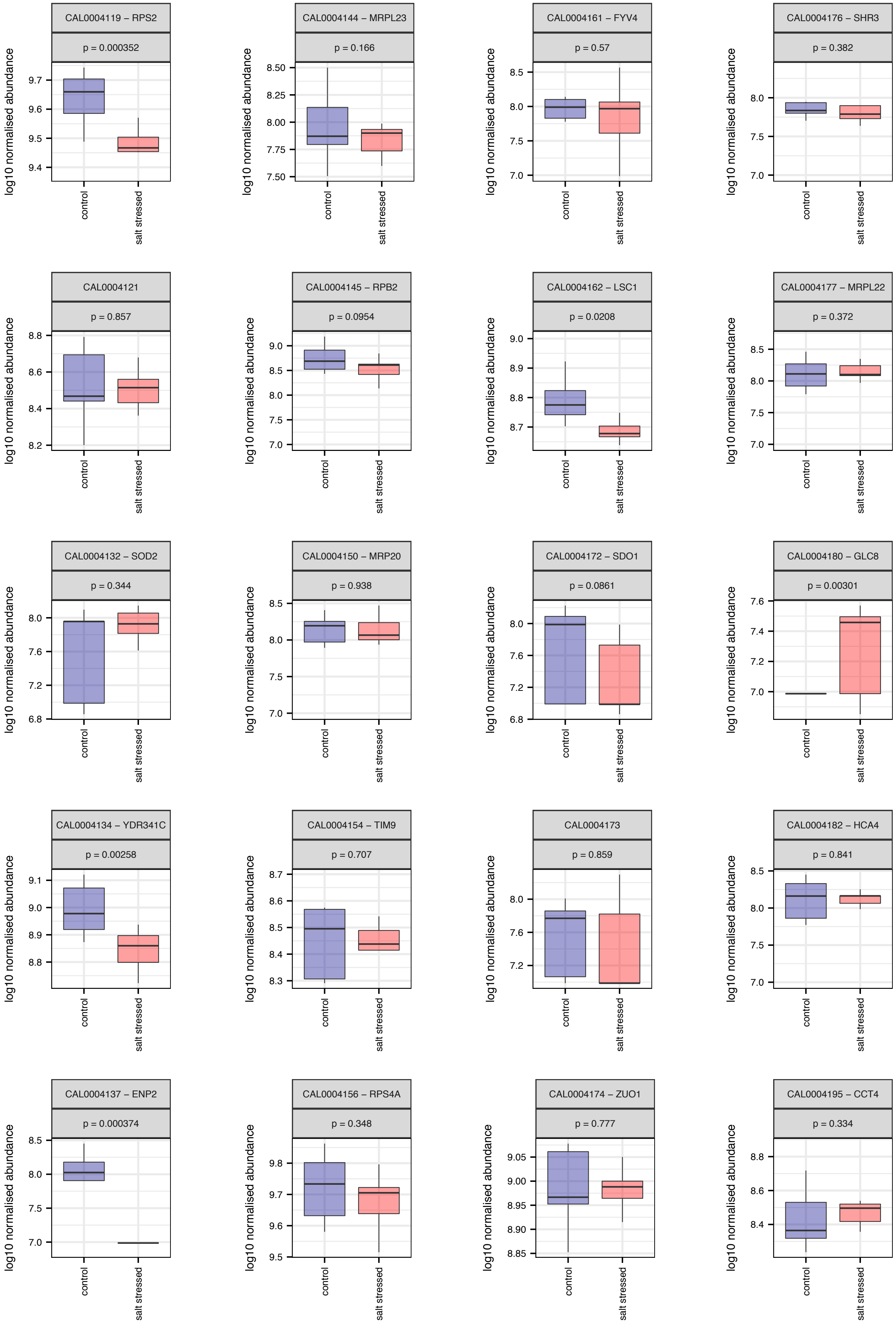
CAL0004119 – RPS2
p = 0.000352
CAL0004144 – MRPL23
p = 0.166
CAL0004161 – FYV4
p = 0.57
CAL0004176 – SHR3
p = 0.382
CAL0004121
p = 0.857
CAL0004145 – RPB2
p = 0.0954
CAL0004162 – LSC1
p = 0.0208
CAL0004177 – MRPL22
p = 0.372
CAL0004132 – SOD2
p = 0.344
CAL0004150 – MRP20
p = 0.938
CAL0004172 – SDO1
p = 0.0861
CAL0004180 – GLC8
p = 0.00301
CAL0004134 – YDR341C
p = 0.00258
CAL0004154 – TIM9
p = 0.707
CAL0004173
p = 0.859
CAL0004182 – HCA4
p = 0.841
CAL0004137 – ENP2
p = 0.000374
CAL0004156 – RPS4A
p = 0.348
CAL0004174 – ZUO1
p = 0.777
CAL0004195 – CCT4
p = 0.334
log10 normalised abundance
control
salt stressed

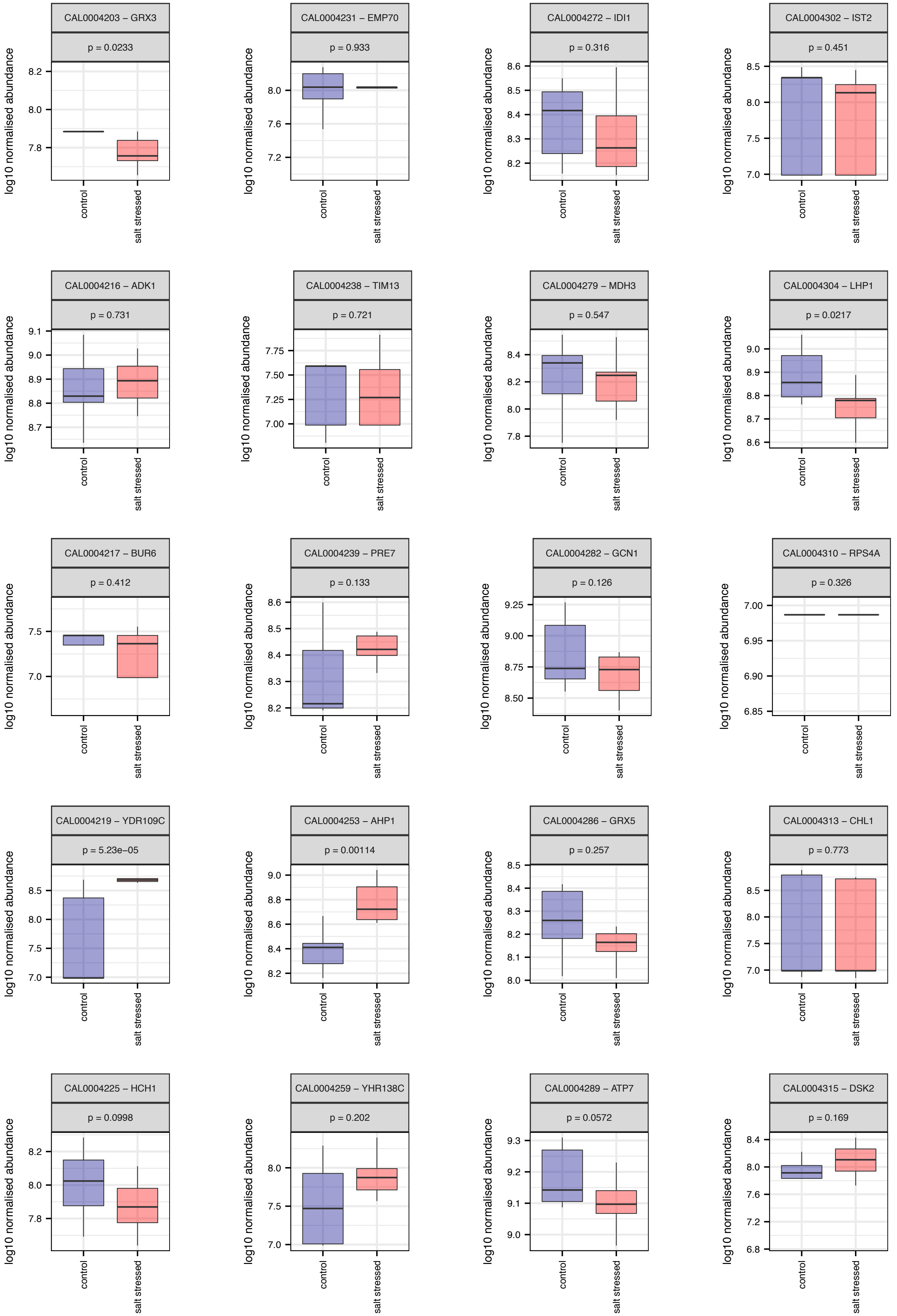
CAL0004203 – GRX3
p = 0.0233
CAL0004231 – EMP70
p = 0.933
CAL0004272 – IDI1
p = 0.316
CAL0004302 – IST2
p = 0.451
CAL0004216 – ADK1
p = 0.731
CAL0004238 – TIM13
p = 0.721
CAL0004279 – MDH3
p = 0.547
CAL0004304 – LHP1
p = 0.0217
CAL0004217 – BUR6
p = 0.412
CAL0004239 – PRE7
p = 0.133
CAL0004282 – GCN1
p = 0.126
CAL0004310 – RPS4A
p = 0.326
CAL0004219 – YDR109C
p = 5.23e–05
CAL0004253 – AHP1
p = 0.00114
CAL0004286 – GRX5
p = 0.257
CAL0004313 – CHL1
p = 0.773
CAL0004225 – HCH1
p = 0.0998
CAL0004259 – YHR138C
p = 0.202
CAL0004289 – ATP7
p = 0.0572
CAL0004315 – DSK2
p = 0.169
log10 normalised abundance
control
salt stressed

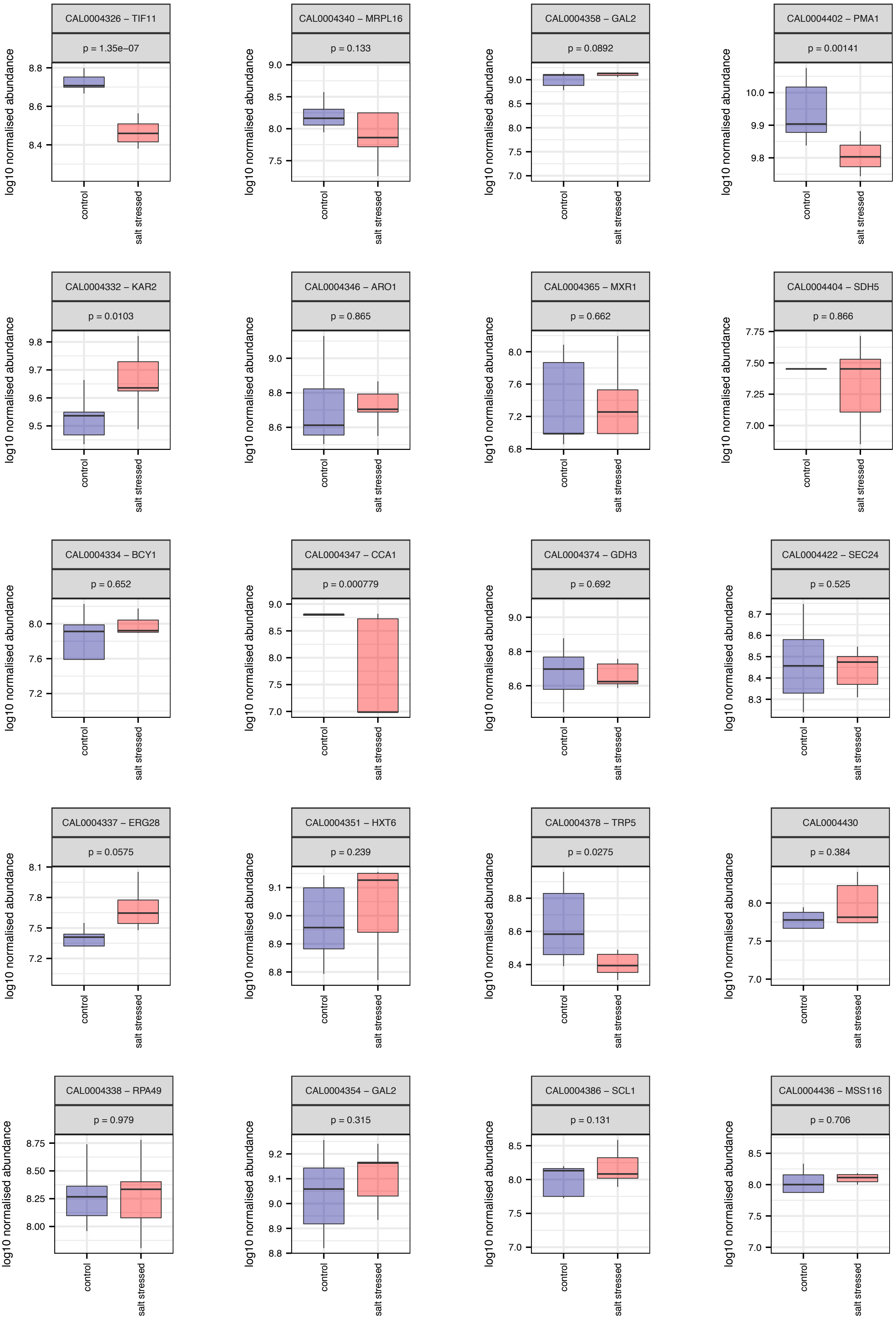
CAL0004326 – TIF11
p = 1.35e–07
CAL0004340 – MRPL16
p = 0.133
CAL0004358 – GAL2
p = 0.0892
CAL0004402 – PMA1
p = 0.00141
CAL0004332 – KAR2
p = 0.0103
CAL0004346 – ARO1
p = 0.865
CAL0004365 – MXR1
p = 0.662
CAL0004404 – SDH5
p = 0.866
CAL0004334 – BCY1
p = 0.652
CAL0004347 – CCA1
p = 0.000779
CAL0004374 – GDH3
p = 0.692
CAL0004422 – SEC24
p = 0.525
CAL0004337 – ERG28
p = 0.0575
CAL0004351 – HXT6
p = 0.239
CAL0004378 – TRP5
p = 0.0275
CAL0004430
p = 0.384
CAL0004338 – RPA49
p = 0.979
CAL0004354 – GAL2
p = 0.315
CAL0004386 – SCL1
p = 0.131
CAL0004436 – MSS116
p = 0.706
log10 normalised abundance
control
salt stressed

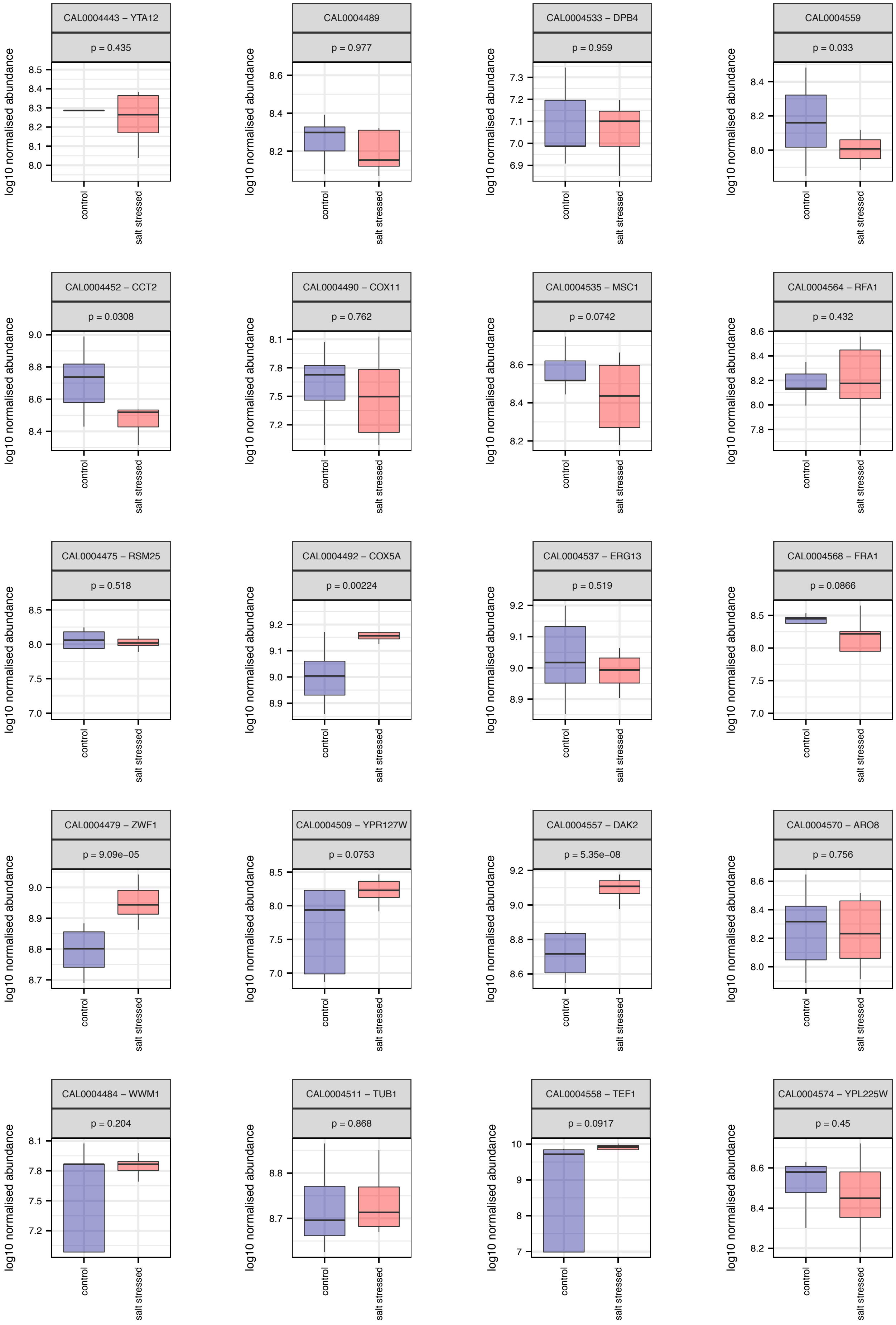
CAL0004443 – YTA12
p = 0.435
CAL0004489
p = 0.977
CAL0004533 – DPB4
p = 0.959
CAL0004559
p = 0.033
CAL0004452 – CCT2
p = 0.0308
CAL0004490 – COX11
p = 0.762
CAL0004535 – MSC1
p = 0.0742
CAL0004564 – RFA1
p = 0.432
CAL0004475 – RSM25
p = 0.518
CAL0004492 – COX5A
p = 0.00224
CAL0004537 – ERG13
p = 0.519
CAL0004568 – FRA1
p = 0.0866
CAL0004479 – ZWF1
p = 9.09e–05
CAL0004509 – YPR127W
p = 0.0753
CAL0004557 – DAK2
p = 5.35e–08
CAL0004570 – ARO8
p = 0.756
CAL0004484 – WWM1
p = 0.204
CAL0004511 – TUB1
p = 0.868
CAL0004558 – TEF1
p = 0.0917
CAL0004574 – YPL225W
p = 0.45
log10 normalised abundance
control
salt stressed

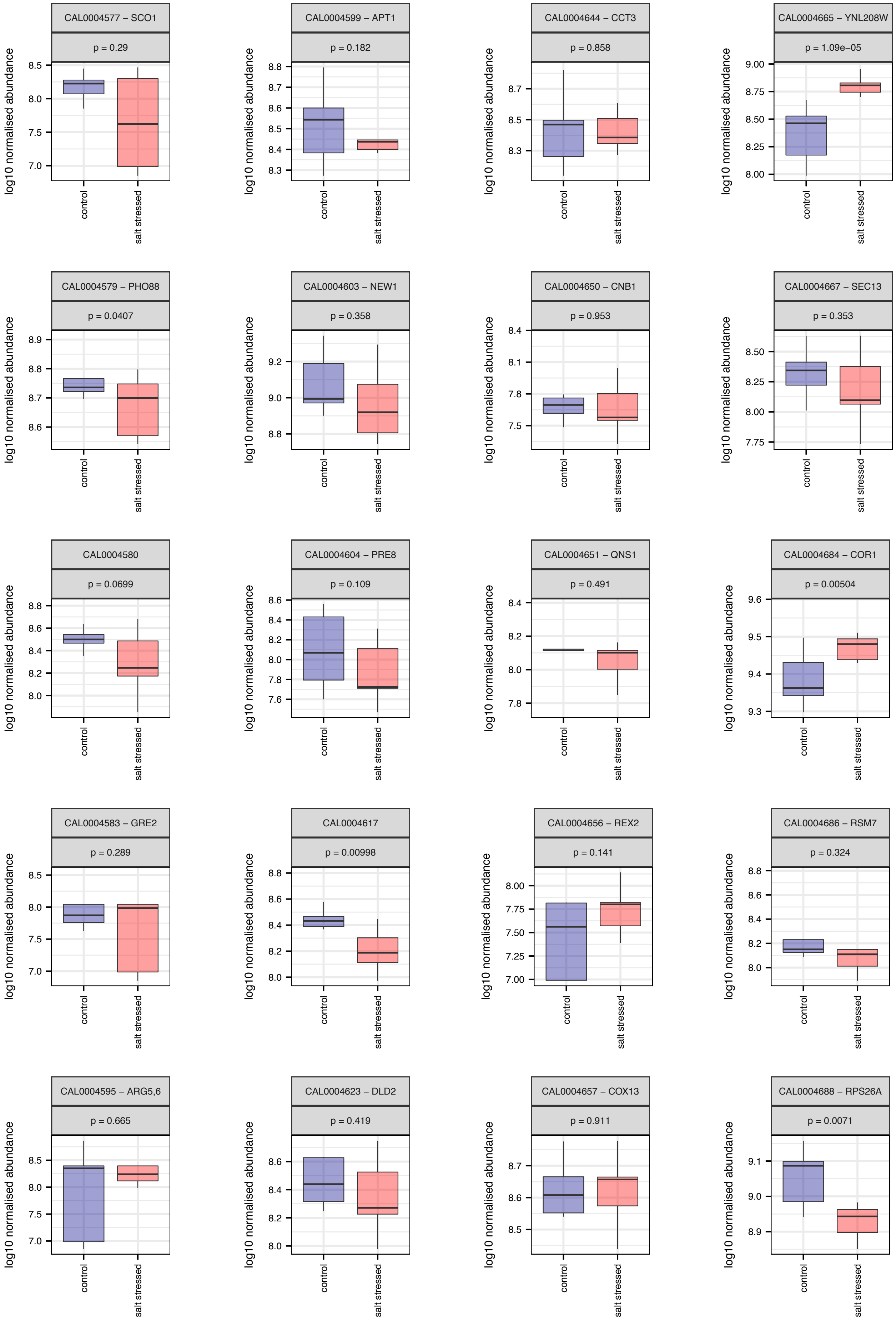
CAL0004577 – SCO1
p = 0.29
CAL0004599 – APT1
p = 0.182
CAL0004644 – CCT3
p = 0.858
CAL0004665 – YNL208W
p = 1.09e−05
CAL0004579 – PHO88
p = 0.0407
CAL0004603 – NEW1
p = 0.358
CAL0004650 – CNB1
p = 0.953
CAL0004667 – SEC13
p = 0.353
CAL0004580
p = 0.0699
CAL0004604 – PRE8
p = 0.109
CAL0004651 – QNS1
p = 0.491
CAL0004684 – COR1
p = 0.00504
CAL0004583 – GRE2
p = 0.289
CAL0004617
p = 0.00998
CAL0004656 – REX2
p = 0.141
CAL0004686 – RSM7
p = 0.324
CAL0004595 – ARG5,6
p = 0.665
CAL0004623 – DLD2
p = 0.419
CAL0004657 – COX13
p = 0.911
CAL0004688 – RPS26A
p = 0.0071
log10 normalised abundance
control
salt stressed

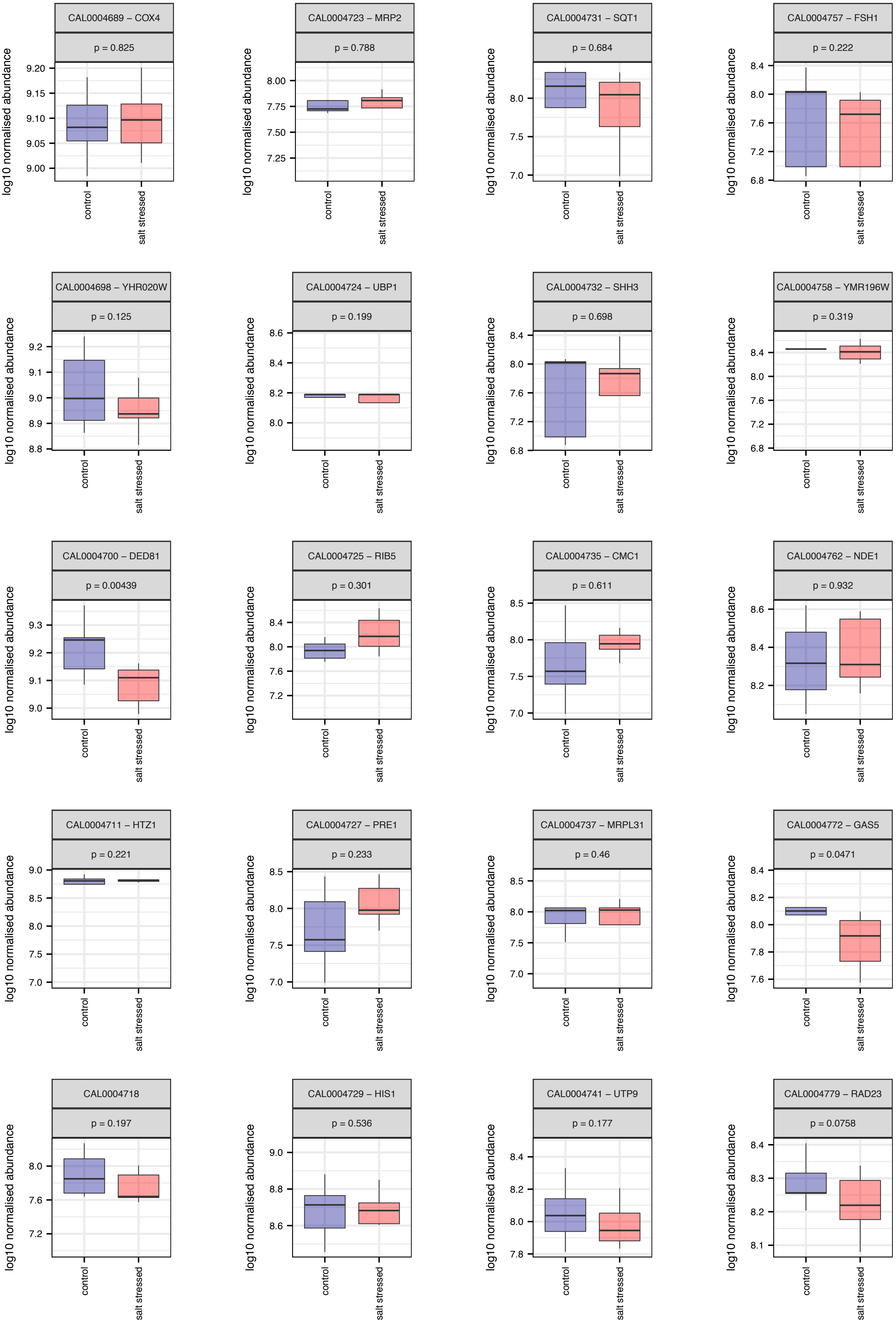
CAL0004689 – COX4
p = 0.825
CAL0004723 – MRP2
p = 0.788
CAL0004731 – SQT1
p = 0.684
CAL0004757 – FSH1
p = 0.222
CAL0004698 – YHR020W
p = 0.125
CAL0004724 – UBP1
p = 0.199
CAL0004732 – SHH3
p = 0.698
CAL0004758 – YMR196W
p = 0.319
CAL0004700 – DED81
p = 0.00439
CAL0004725 – RIB5
p = 0.301
CAL0004735 – CMC1
p = 0.611
CAL0004762 – NDE1
p = 0.932
CAL0004711 – HTZ1
p = 0.221
CAL0004727 – PRE1
p = 0.233
CAL0004737 – MRPL31
p = 0.46
CAL0004772 – GAS5
p = 0.0471
CAL0004718
p = 0.197
CAL0004729 – HIS1
p = 0.536
CAL0004741 – UTP9
p = 0.177
CAL0004779 – RAD23
p = 0.0758
log10 normalised abundance
control
salt stressed

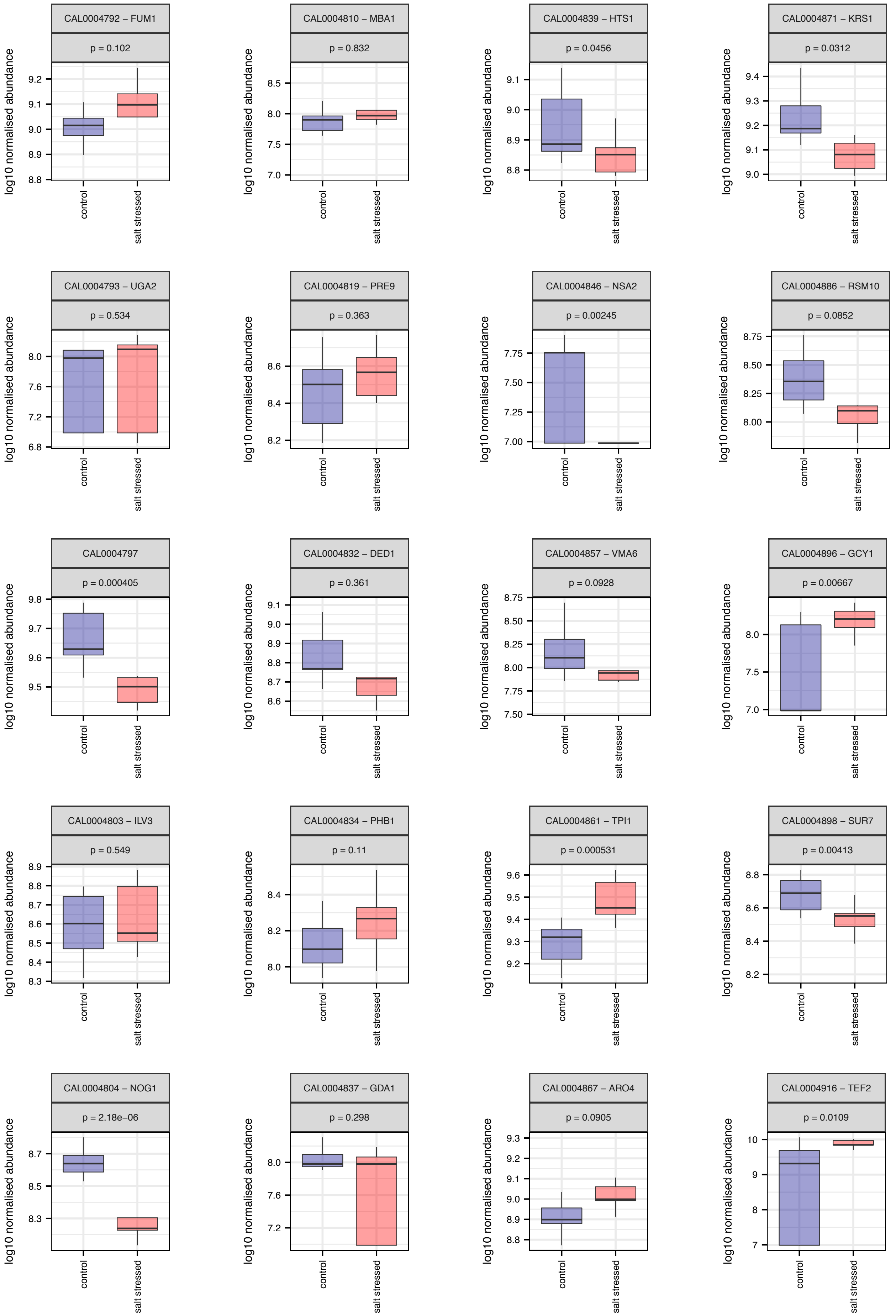
CAL0004792 – FUM1
p = 0.102
CAL0004810 – MBA1
p = 0.832
CAL0004839 – HTS1
p = 0.0456
CAL0004871 – KRS1
p = 0.0312
CAL0004793 – UGA2
p = 0.534
CAL0004819 – PRE9
p = 0.363
CAL0004846 – NSA2
p = 0.00245
CAL0004886 – RSM10
p = 0.0852
CAL0004797
p = 0.000405
CAL0004832 – DED1
p = 0.361
CAL0004857 – VMA6
p = 0.0928
CAL0004896 – GCY1
p = 0.00667
CAL0004803 – ILV3
p = 0.549
CAL0004834 – PHB1
p = 0.11
CAL0004861 – TPI1
p = 0.000531
CAL0004898 – SUR7
p = 0.00413
CAL0004804 – NOG1
p = 2.18e–06
CAL0004837 – GDA1
p = 0.298
CAL0004867 – ARO4
p = 0.0905
CAL0004916 – TEF2
p = 0.0109
log10 normalised abundance
control
salt stressed

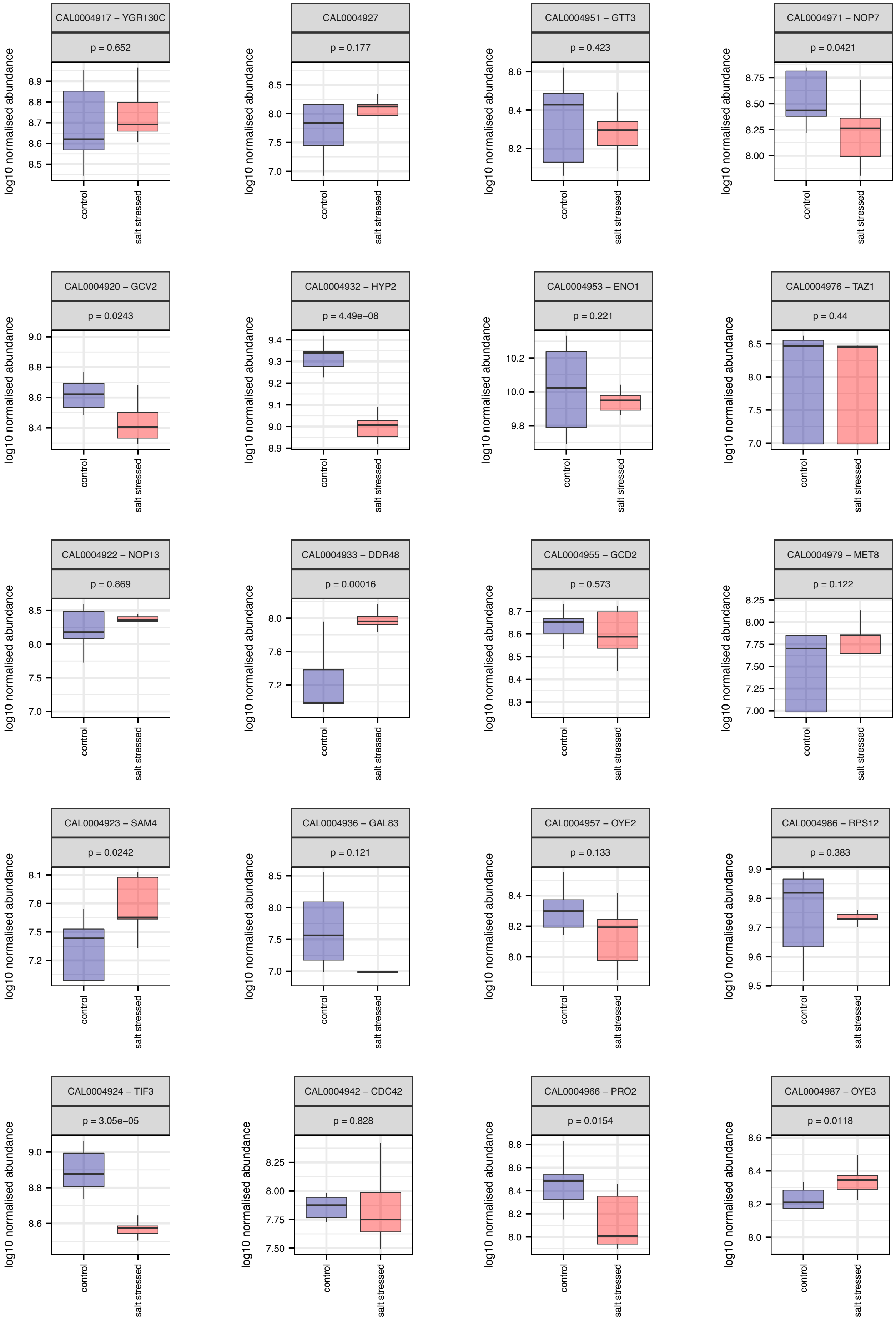
CAL0004917 – YGR130C
p = 0.652
CAL0004927
p = 0.177
CAL0004951 – GTT3
p = 0.423
CAL0004971 – NOP7
p = 0.0421
CAL0004920 – GCV2
p = 0.0243
CAL0004932 – HYP2
p = 4.49e–08
CAL0004953 – ENO1
p = 0.221
CAL0004976 – TAZ1
p = 0.44
CAL0004922 – NOP13
p = 0.869
CAL0004933 – DDR48
p = 0.00016
CAL0004955 – GCD2
p = 0.573
CAL0004979 – MET8
p = 0.122
CAL0004923 – SAM4
p = 0.0242
CAL0004936 – GAL83
p = 0.121
CAL0004957 – OYE2
p = 0.133
CAL0004986 – RPS12
p = 0.383
CAL0004924 – TIF3
p = 3.05e–05
CAL0004942 – CDC42
p = 0.828
CAL0004966 – PRO2
p = 0.0154
CAL0004987 – OYE3
p = 0.0118
log10 normalised abundance
control
salt stressed

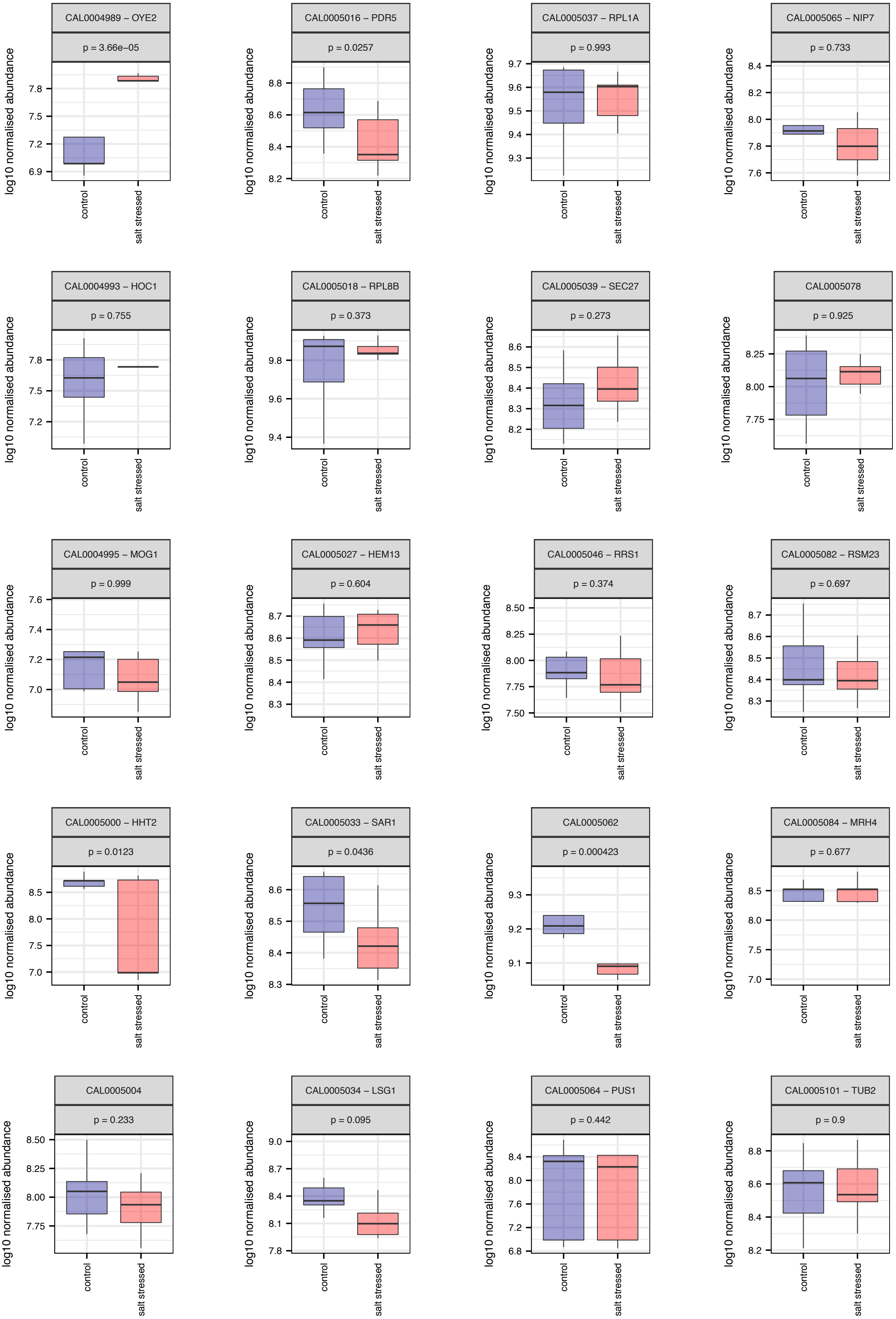
CAL0004989 – OYE2
p = 3.66e–05
CAL0005016 – PDR5
p = 0.0257
CAL0005037 – RPL1A
p = 0.993
CAL0005065 – NIP7
p = 0.733
CAL0004993 – HOC1
p = 0.755
CAL0005018 – RPL8B
p = 0.373
CAL0005039 – SEC27
p = 0.273
CAL0005078
p = 0.925
CAL0004995 – MOG1
p = 0.999
CAL0005027 – HEM13
p = 0.604
CAL0005046 – RRS1
p = 0.374
CAL0005082 – RSM23
p = 0.697
CAL0005000 – HHT2
p = 0.0123
CAL0005033 – SAR1
p = 0.0436
CAL0005062
p = 0.000423
CAL0005084 – MRH4
p = 0.677
CAL0005004
p = 0.233
CAL0005034 – LSG1
p = 0.095
CAL0005064 – PUS1
p = 0.442
CAL0005101 – TUB2
p = 0.9
log10 normalised abundance
control
salt stressed

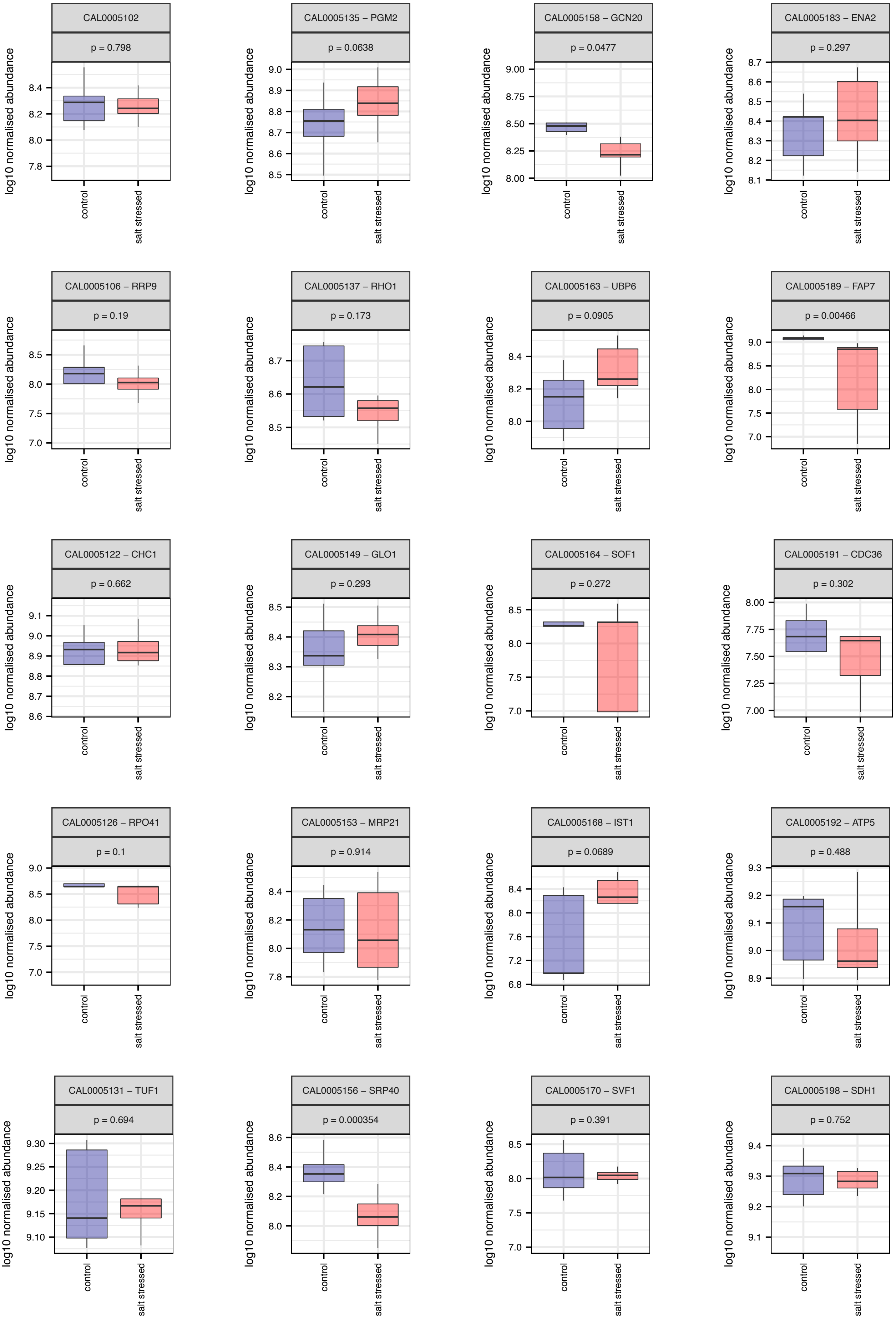
CAL0005102
p = 0.798
CAL0005135 – PGM2
p = 0.0638
CAL0005158 – GCN20
p = 0.0477
CAL0005183 – ENA2
p = 0.297
CAL0005106 – RRP9
p = 0.19
CAL0005137 – RHO1
p = 0.173
CAL0005163 – UBP6
p = 0.0905
CAL0005189 – FAP7
p = 0.00466
CAL0005122 – CHC1
p = 0.662
CAL0005149 – GLO1
p = 0.293
CAL0005164 – SOF1
p = 0.272
CAL0005191 – CDC36
p = 0.302
CAL0005126 – RPO41
p = 0.1
CAL0005153 – MRP21
p = 0.914
CAL0005168 – IST1
p = 0.0689
CAL0005192 – ATP5
p = 0.488
CAL0005131 – TUF1
p = 0.694
CAL0005156 – SRP40
p = 0.000354
CAL0005170 – SVF1
p = 0.391
CAL0005198 – SDH1
p = 0.752
log10 normalised abundance
control
salt stressed

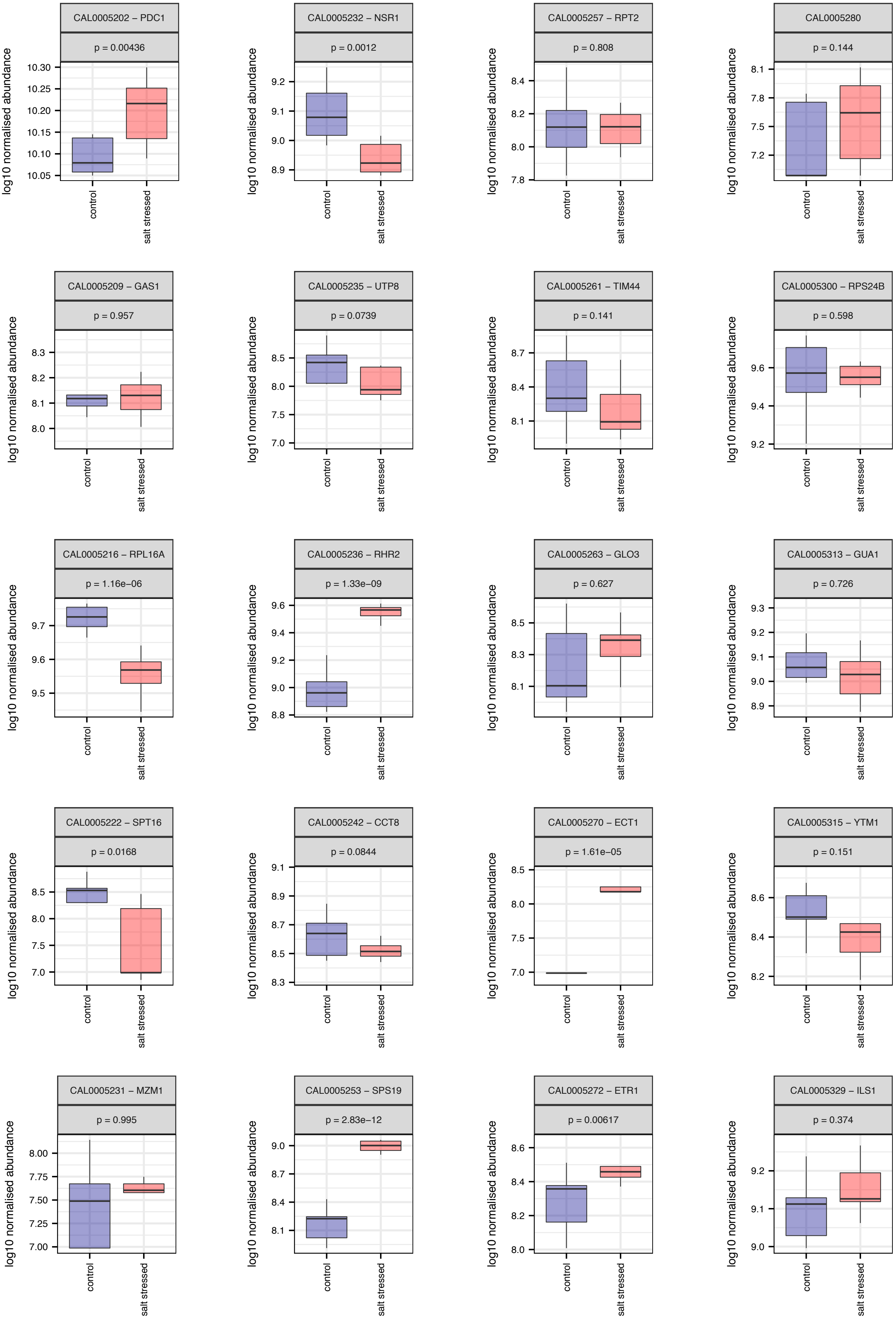
CAL0005202 – PDC1
p = 0.00436
CAL0005232 – NSR1
p = 0.0012
CAL0005257 – RPT2
p = 0.808
CAL0005280
p = 0.144
CAL0005209 – GAS1
p = 0.957
CAL0005235 – UTP8
p = 0.0739
CAL0005261 – TIM44
p = 0.141
CAL0005300 – RPS24B
p = 0.598
CAL0005216 – RPL16A
p = 1.16e–06
CAL0005236 – RHR2
p = 1.33e–09
CAL0005263 – GLO3
p = 0.627
CAL0005313 – GUA1
p = 0.726
CAL0005222 – SPT16
p = 0.0168
CAL0005242 – CCT8
p = 0.0844
CAL0005270 – ECT1
p = 1.61e–05
CAL0005315 – YTM1
p = 0.151
CAL0005231 – MZM1
p = 0.995
CAL0005253 – SPS19
p = 2.83e–12
CAL0005272 – ETR1
p = 0.00617
CAL0005329 – ILS1
p = 0.374
log10 normalised abundance
control
salt stressed

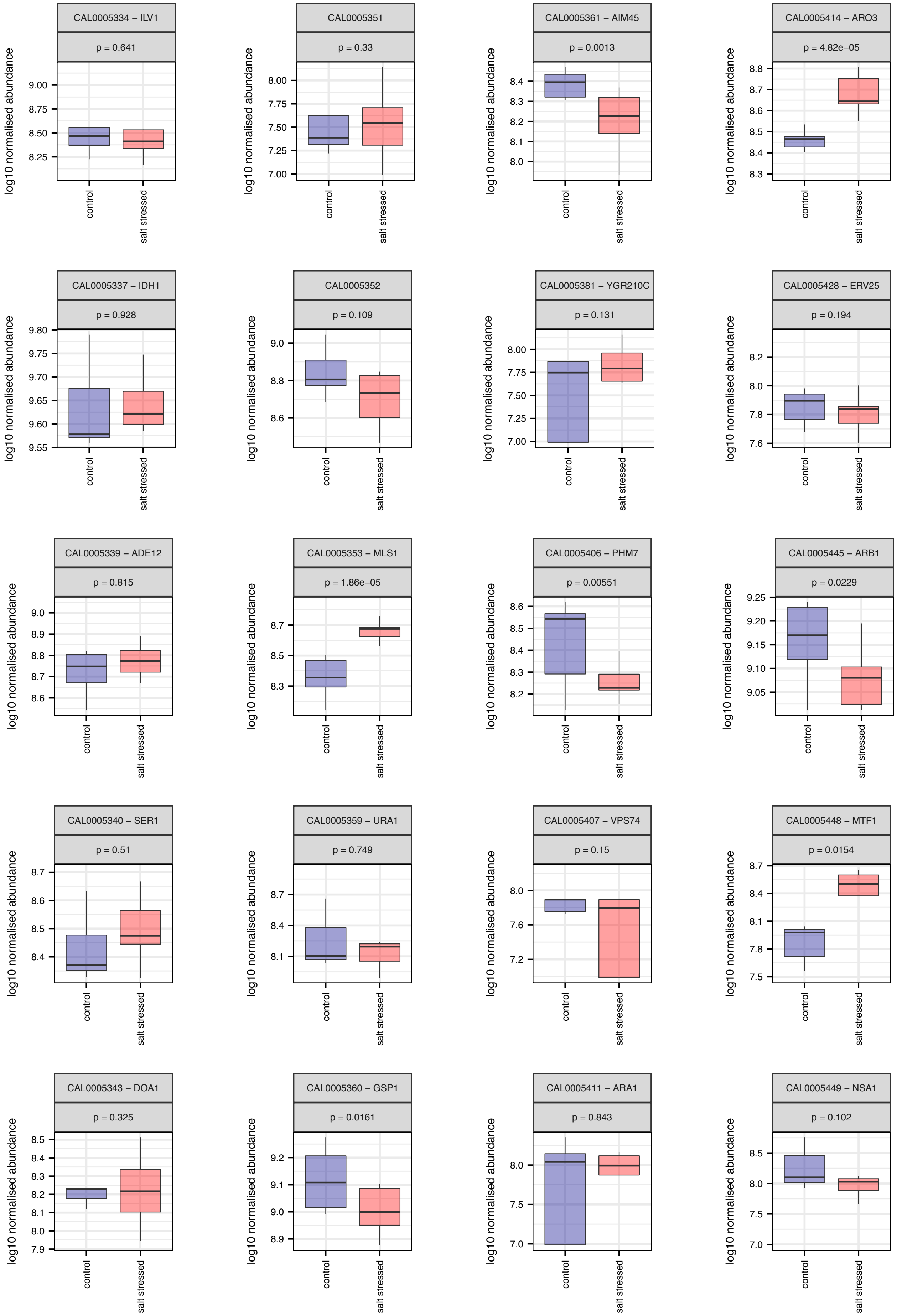
CAL0005334 – ILV1
p = 0.641
CAL0005351
p = 0.33
CAL0005361 – AIM45
p = 0.0013
CAL0005414 – ARO3
p = 4.82e–05
CAL0005337 – IDH1
p = 0.928
CAL0005352
p = 0.109
CAL0005381 – YGR210C
p = 0.131
CAL0005428 – ERV25
p = 0.194
CAL0005339 – ADE12
p = 0.815
CAL0005353 – MLS1
p = 1.86e–05
CAL0005406 – PHM7
p = 0.00551
CAL0005445 – ARB1
p = 0.0229
CAL0005340 – SER1
p = 0.51
CAL0005359 – URA1
p = 0.749
CAL0005407 – VPS74
p = 0.15
CAL0005448 – MTF1
p = 0.0154
CAL0005343 – DOA1
p = 0.325
CAL0005360 – GSP1
p = 0.0161
CAL0005411 – ARA1
p = 0.843
CAL0005449 – NSA1
p = 0.102
log10 normalised abundance
control
salt stressed

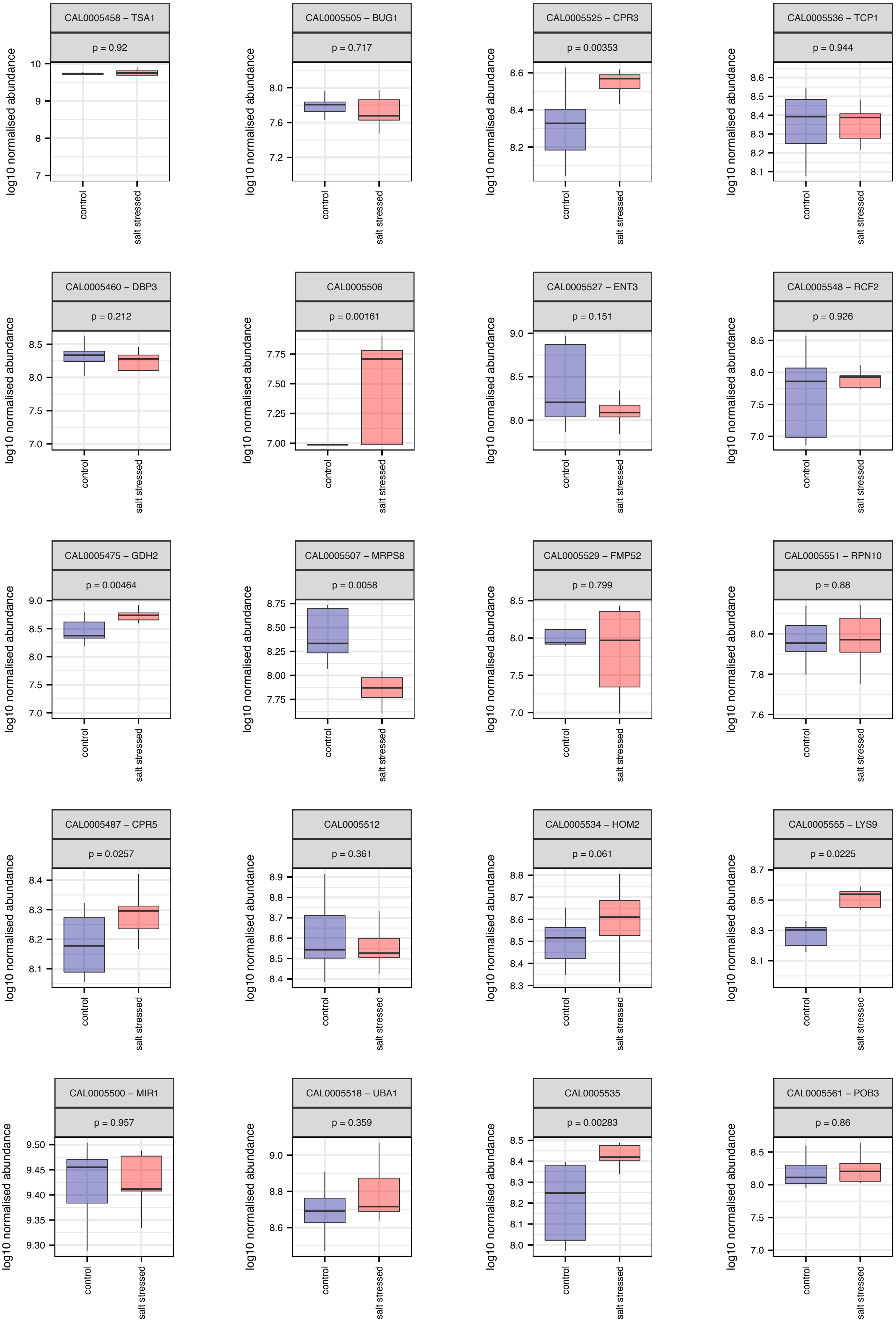
CAL0005458 – TSA1
p = 0.92
CAL0005505 – BUG1
p = 0.717
CAL0005525 – CPR3
p = 0.00353
CAL0005536 – TCP1
p = 0.944
CAL0005460 – DBP3
p = 0.212
CAL0005506
p = 0.00161
CAL0005527 – ENT3
p = 0.151
CAL0005548 – RCF2
p = 0.926
CAL0005475 – GDH2
p = 0.00464
CAL0005507 – MRPS8
p = 0.0058
CAL0005529 – FMP52
p = 0.799
CAL0005551 – RPN10
p = 0.88
CAL0005487 – CPR5
p = 0.0257
CAL0005512
p = 0.361
CAL0005534 – HOM2
p = 0.061
CAL0005555 – LYS9
p = 0.0225
CAL0005500 – MIR1
p = 0.957
CAL0005518 – UBA1
p = 0.359
CAL0005535
p = 0.00283
CAL0005561 – POB3
p = 0.86
log10 normalised abundance
control
salt stressed

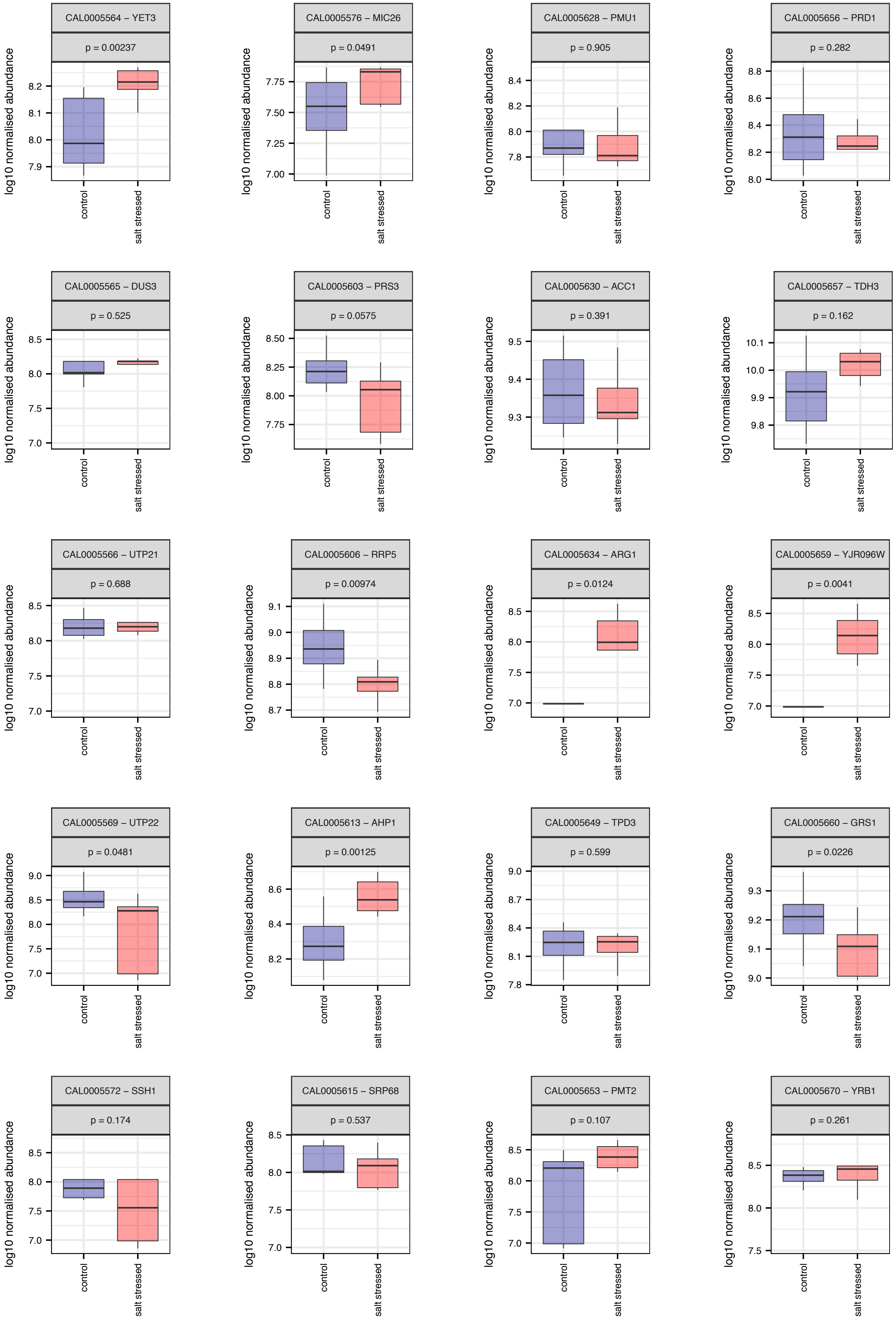
CAL0005564 – YET3
p = 0.00237
CAL0005576 – MIC26
p = 0.0491
CAL0005628 – PMU1
p = 0.905
CAL0005656 – PRD1
p = 0.282
CAL0005565 – DUS3
p = 0.525
CAL0005603 – PRS3
p = 0.0575
CAL0005630 – ACC1
p = 0.391
CAL0005657 – TDH3
p = 0.162
CAL0005566 – UTP21
p = 0.688
CAL0005606 – RRP5
p = 0.00974
CAL0005634 – ARG1
p = 0.0124
CAL0005659 – YJR096W
p = 0.0041
CAL0005569 – UTP22
p = 0.0481
CAL0005613 – AHP1
p = 0.00125
CAL0005649 – TPD3
p = 0.599
CAL0005660 – GRS1
p = 0.0226
CAL0005572 – SSH1
p = 0.174
CAL0005615 – SRP68
p = 0.537
CAL0005653 – PMT2
p = 0.107
CAL0005670 – YRB1
p = 0.261
log10 normalised abundance
control
salt stressed

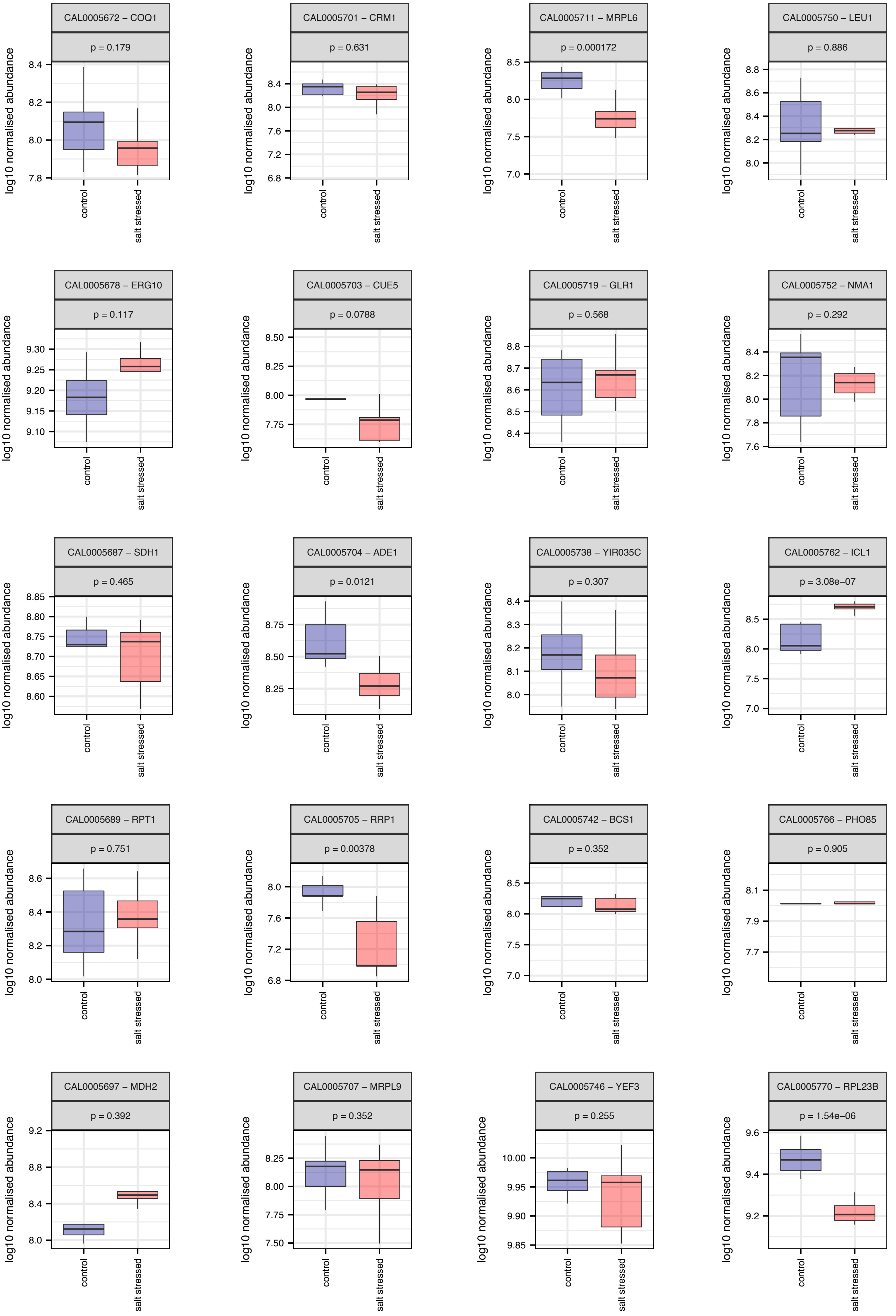
CAL0005672 – COQ1
p = 0.179
CAL0005701 – CRM1
p = 0.631
CAL0005711 – MRPL6
p = 0.000172
CAL0005750 – LEU1
p = 0.886
CAL0005678 – ERG10
p = 0.117
CAL0005703 – CUE5
p = 0.0788
CAL0005719 – GLR1
p = 0.568
CAL0005752 – NMA1
p = 0.292
CAL0005687 – SDH1
p = 0.465
CAL0005704 – ADE1
p = 0.0121
CAL0005738 – YIR035C
p = 0.307
CAL0005762 – ICL1
p = 3.08e−07
CAL0005689 – RPT1
p = 0.751
CAL0005705 – RRP1
p = 0.00378
CAL0005742 – BCS1
p = 0.352
CAL0005766 – PHO85
p = 0.905
CAL0005697 – MDH2
p = 0.392
CAL0005707 – MRPL9
p = 0.352
CAL0005746 – YEF3
p = 0.255
CAL0005770 – RPL23B
p = 1.54e−06
log10 normalised abundance
control
salt stressed

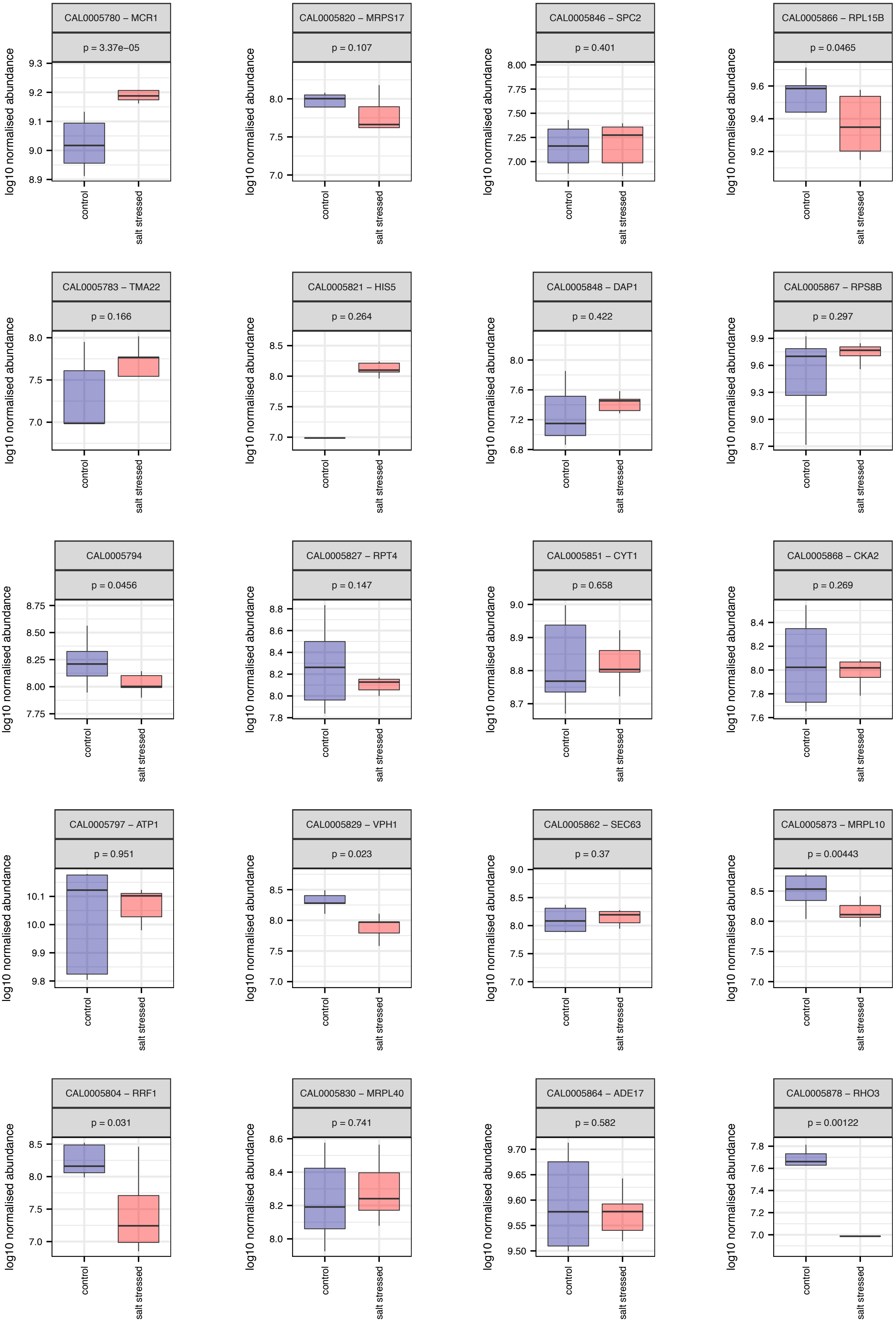
CAL0005780 – MCR1
p = 3.37e–05
CAL0005820 – MRPS17
p = 0.107
CAL0005846 – SPC2
p = 0.401
CAL0005866 – RPL15B
p = 0.0465
CAL0005783 – TMA22
p = 0.166
CAL0005821 – HIS5
p = 0.264
CAL0005848 – DAP1
p = 0.422
CAL0005867 – RPS8B
p = 0.297
CAL0005794
p = 0.0456
CAL0005827 – RPT4
p = 0.147
CAL0005851 – CYT1
p = 0.658
CAL0005868 – CKA2
p = 0.269
CAL0005797 – ATP1
p = 0.951
CAL0005829 – VPH1
p = 0.023
CAL0005862 – SEC63
p = 0.37
CAL0005873 – MRPL10
p = 0.00443
CAL0005804 – RRF1
p = 0.031
CAL0005830 – MRPL40
p = 0.741
CAL0005864 – ADE17
p = 0.582
CAL0005878 – RHO3
p = 0.00122
log10 normalised abundance
control
salt stressed

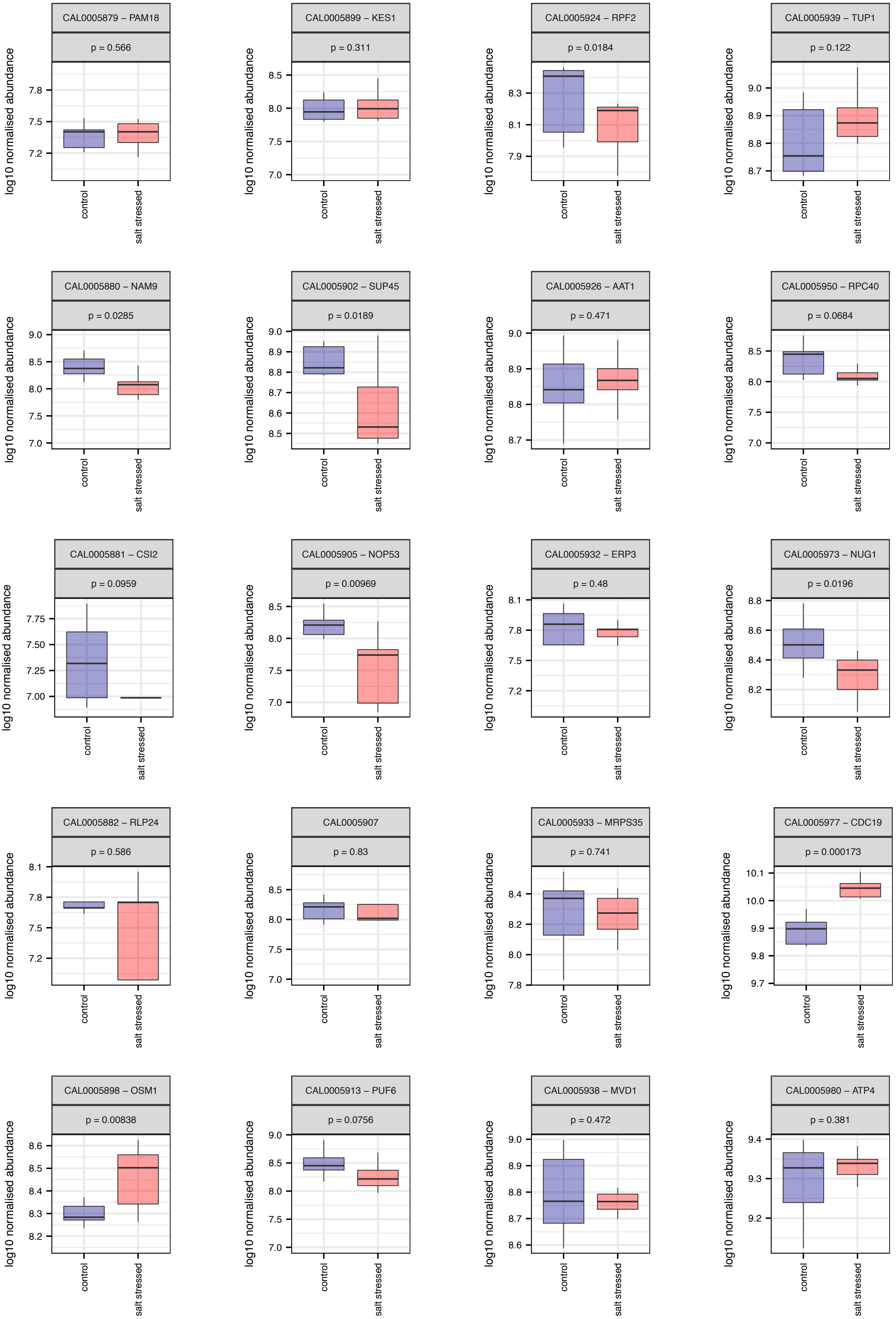
CAL0005879 – PAM18
p = 0.566
CAL0005899 – KES1
p = 0.311
CAL0005924 – RPF2
p = 0.0184
CAL0005939 – TUP1
p = 0.122
CAL0005880 – NAM9
p = 0.0285
CAL0005902 – SUP45
p = 0.0189
CAL0005926 – AAT1
p = 0.471
CAL0005950 – RPC40
p = 0.0684
CAL0005881 – CSI2
p = 0.0959
CAL0005905 – NOP53
p = 0.00969
CAL0005932 – ERP3
p = 0.48
CAL0005973 – NUG1
p = 0.0196
CAL0005882 – RLP24
p = 0.586
CAL0005907
p = 0.83
CAL0005933 – MRPS35
p = 0.741
CAL0005977 – CDC19
p = 0.000173
CAL0005898 – OSM1
p = 0.00838
CAL0005913 – PUF6
p = 0.0756
CAL0005938 – MVD1
p = 0.472
CAL0005980 – ATP4
p = 0.381
log10 normalised abundance
control
salt stressed

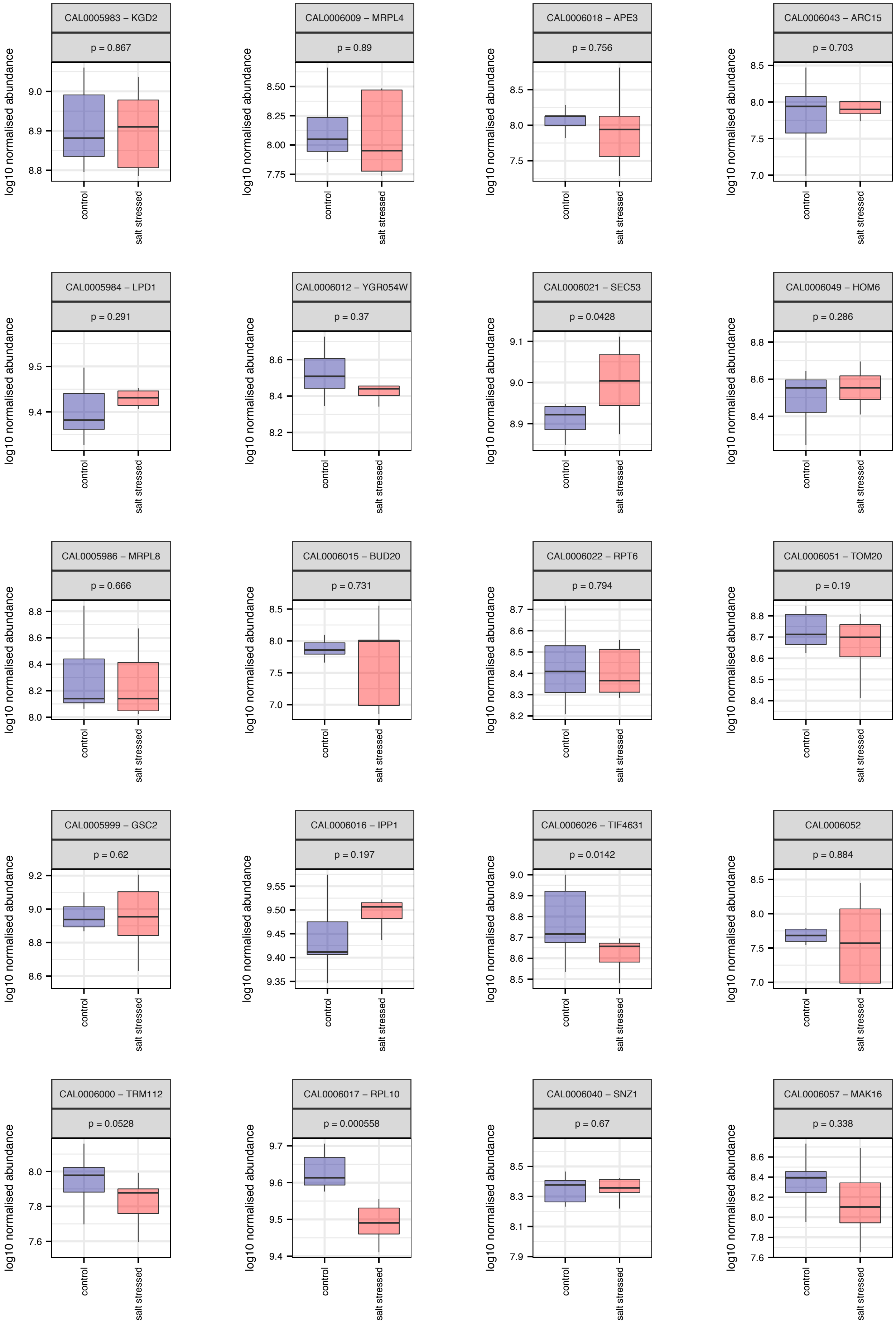
CAL0005983 – KGD2
p = 0.867
CAL0006009 – MRPL4
p = 0.89
CAL0006018 – APE3
p = 0.756
CAL0006043 – ARC15
p = 0.703
CAL0005984 – LPD1
p = 0.291
CAL0006012 – YGR054W
p = 0.37
CAL0006021 – SEC53
p = 0.0428
CAL0006049 – HOM6
p = 0.286
CAL0005986 – MRPL8
p = 0.666
CAL0006015 – BUD20
p = 0.731
CAL0006022 – RPT6
p = 0.794
CAL0006051 – TOM20
p = 0.19
CAL0005999 – GSC2
p = 0.62
CAL0006016 – IPP1
p = 0.197
CAL0006026 – TIF4631
p = 0.0142
CAL0006052
p = 0.884
CAL0006000 – TRM112
p = 0.0528
CAL0006017 – RPL10
p = 0.000558
CAL0006040 – SNZ1
p = 0.67
CAL0006057 – MAK16
p = 0.338
log10 normalised abundance
control
salt stressed

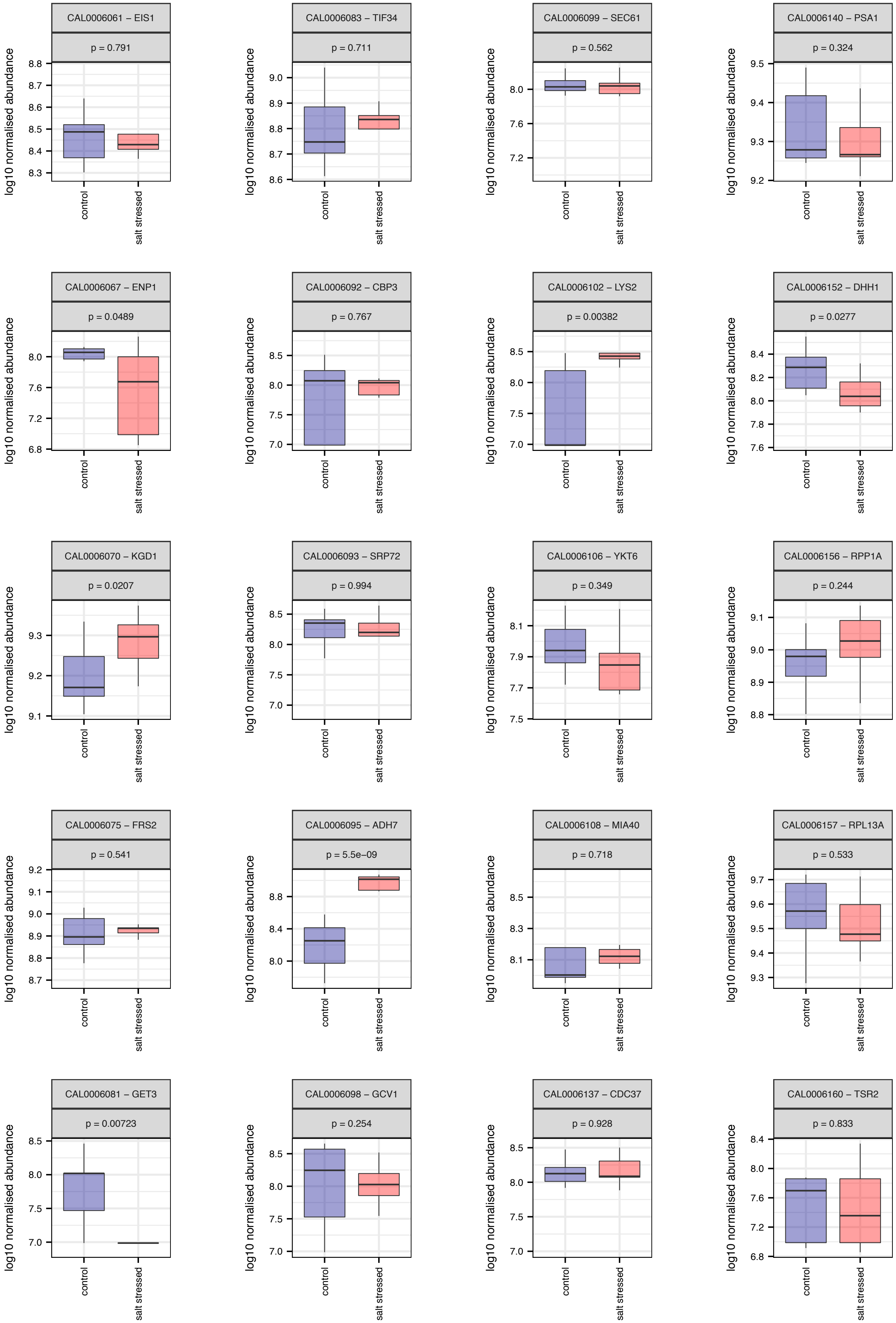
CAL0006061 – EIS1
p = 0.791
CAL0006083 – TIF34
p = 0.711
CAL0006099 – SEC61
p = 0.562
CAL0006140 – PSA1
p = 0.324
CAL0006067 – ENP1
p = 0.0489
CAL0006092 – CBP3
p = 0.767
CAL0006102 – LYS2
p = 0.00382
CAL0006152 – DHH1
p = 0.0277
CAL0006070 – KGD1
p = 0.0207
CAL0006093 – SRP72
p = 0.994
CAL0006106 – YKT6
p = 0.349
CAL0006156 – RPP1A
p = 0.244
CAL0006075 – FRS2
p = 0.541
CAL0006095 – ADH7
p = 5.5e–09
CAL0006108 – MIA40
p = 0.718
CAL0006157 – RPL13A
p = 0.533
CAL0006081 – GET3
p = 0.00723
CAL0006098 – GCV1
p = 0.254
CAL0006137 – CDC37
p = 0.928
CAL0006160 – TSR2
p = 0.833
log10 normalised abundance
control
salt stressed

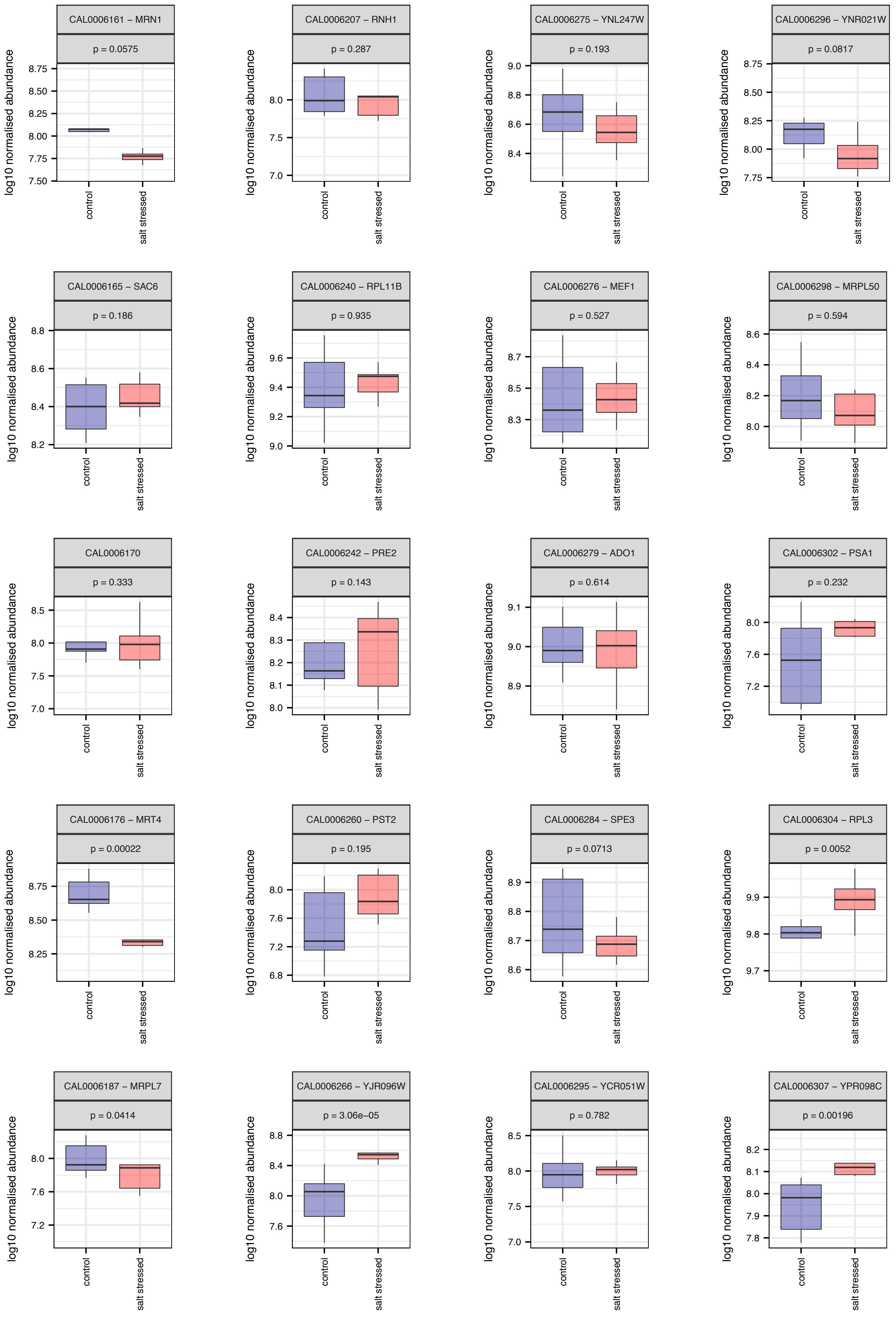
CAL0006161 – MRN1
p = 0.0575
CAL0006207 – RNH1
p = 0.287
CAL0006275 – YNL247W
p = 0.193
CAL0006296 – YNR021W
p = 0.0817
CAL0006165 – SAC6
p = 0.186
CAL0006240 – RPL11B
p = 0.935
CAL0006276 – MEF1
p = 0.527
CAL0006298 – MRPL50
p = 0.594
CAL0006170
p = 0.333
CAL0006242 – PRE2
p = 0.143
CAL0006279 – ADO1
p = 0.614
CAL0006302 – PSA1
p = 0.232
CAL0006176 – MRT4
p = 0.00022
CAL0006260 – PST2
p = 0.195
CAL0006284 – SPE3
p = 0.0713
CAL0006304 – RPL3
p = 0.0052
CAL0006187 – MRPL7
p = 0.0414
CAL0006266 – YJR096W
p = 3.06e−05
CAL0006295 – YCR051W
p = 0.782
CAL0006307 – YPR098C
p = 0.00196
log10 normalised abundance
control
salt stressed

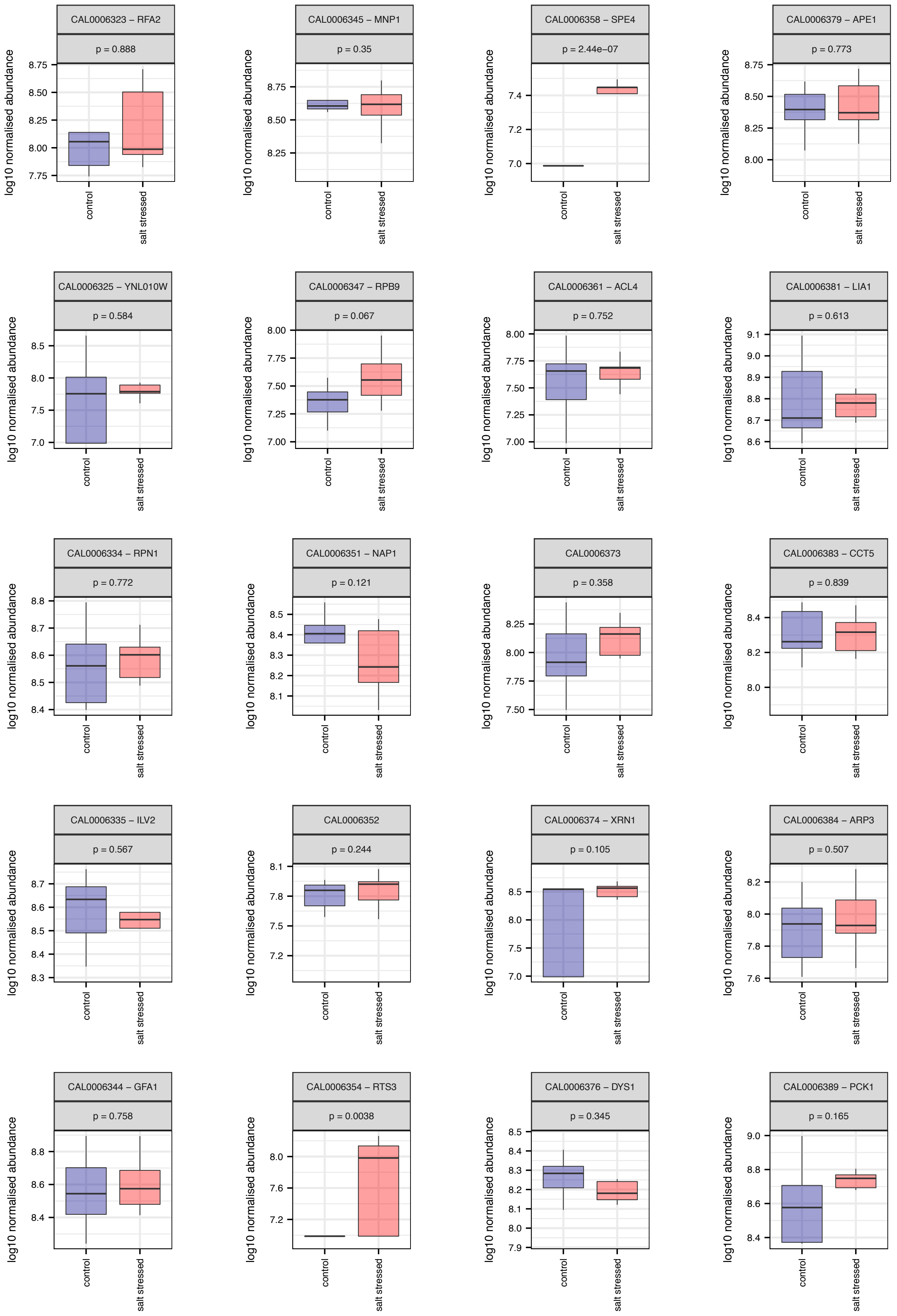
CAL0006323 – RFA2
p = 0.888
CAL0006345 – MNP1
p = 0.35
CAL0006358 – SPE4
p = 2.44e–07
CAL0006379 – APE1
p = 0.773
CAL0006325 – YNL010W
p = 0.584
CAL0006347 – RPB9
p = 0.067
CAL0006361 – ACL4
p = 0.752
CAL0006381 – LIA1
p = 0.613
CAL0006334 – RPN1
p = 0.772
CAL0006351 – NAP1
p = 0.121
CAL0006373
p = 0.358
CAL0006383 – CCT5
p = 0.839
CAL0006335 – ILV2
p = 0.567
CAL0006352
p = 0.244
CAL0006374 – XRN1
p = 0.105
CAL0006384 – ARP3
p = 0.507
CAL0006344 – GFA1
p = 0.758
CAL0006354 – RTS3
p = 0.0038
CAL0006376 – DYS1
p = 0.345
CAL0006389 – PCK1
p = 0.165
log10 normalised abundance
control
salt stressed

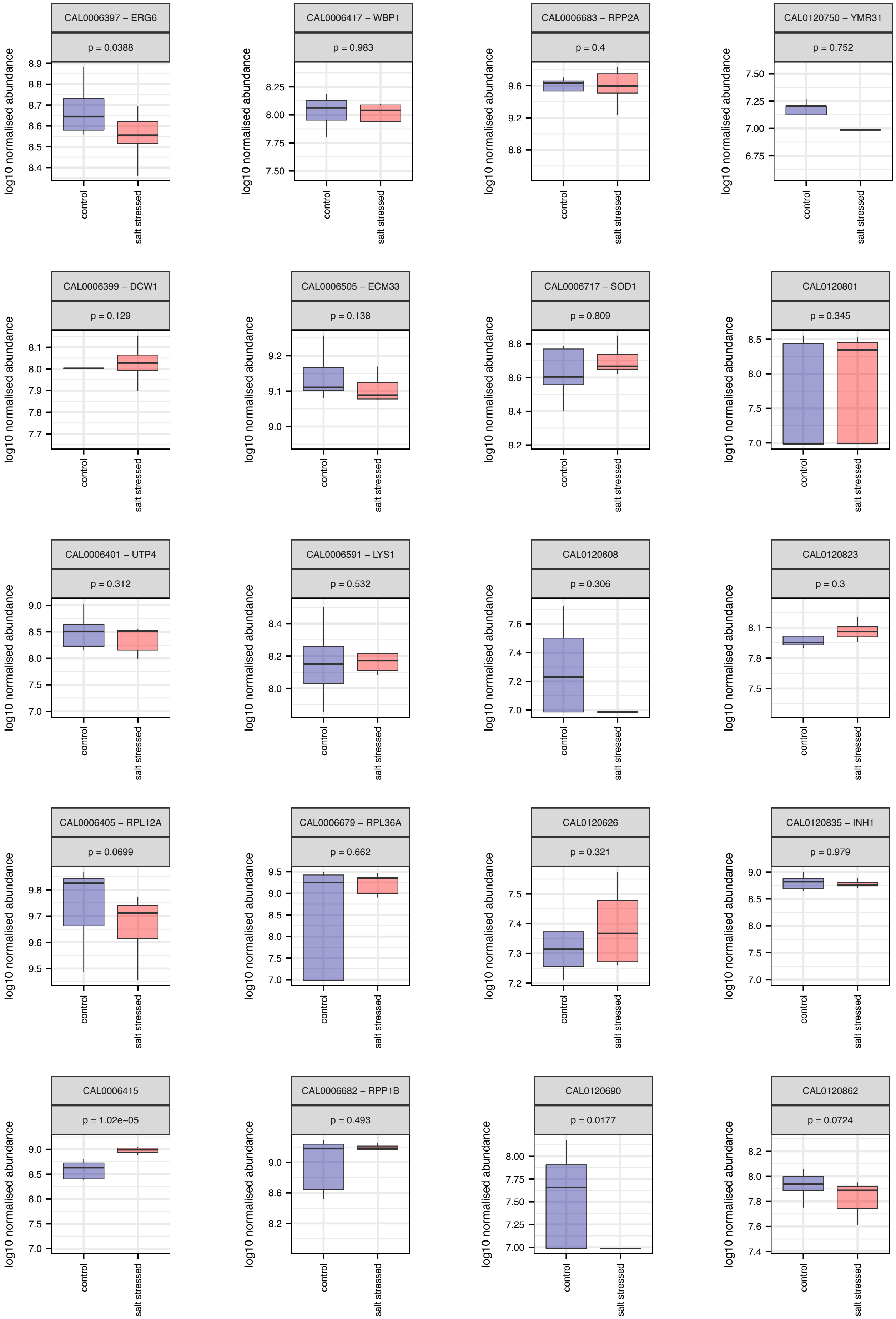
CAL0006397 – ERG6
p = 0.0388
CAL0006417 – WBP1
p = 0.983
CAL0006683 – RPP2A
p = 0.4
CAL0120750 – YMR31
p = 0.752
CAL0006399 – DCW1
p = 0.129
CAL0006505 – ECM33
p = 0.138
CAL0006717 – SOD1
p = 0.809
CAL0120801
p = 0.345
CAL0006401 – UTP4
p = 0.312
CAL0006591 – LYS1
p = 0.532
CAL0120608
p = 0.306
CAL0120823
p = 0.3
CAL0006405 – RPL12A
p = 0.0699
CAL0006679 – RPL36A
p = 0.662
CAL0120626
p = 0.321
CAL0120835 – INH1
p = 0.979
CAL0006415
p = 1.02e–05
CAL0006682 – RPP1B
p = 0.493
CAL0120690
p = 0.0177
CAL0120862
p = 0.0724
log10 normalised abundance
control
salt stressed

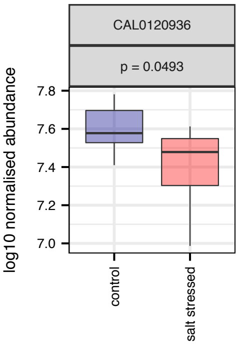
CAL0120936
p = 0.0493
log10 normalised abundance
7.8
7.6
7.4
7.2
7.0
control
salt stressed

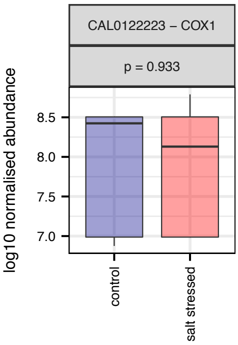
CAL0122223 – COX1
p = 0.933
log10 normalised abundance
8.5
8.0
7.5
7.0
control
salt stressed
